# The Effect of Sulfur Vacancy Distribution on Charge Transport across MoS_2_ Monolayers: A Quantum Mechanical Study

**DOI:** 10.1021/acsmaterialsau.4c00171

**Published:** 2025-06-06

**Authors:** Hanna Kuperman Benedik, Naomi Rom, Maytal Caspary Toroker

**Affiliations:** Department of Materials Science and Engineering, The Nancy and Stephen Grand Technion Energy Program, The Resnick Sustainability Center for Catalysis, 26747 Technion-Israel Institute of Technology, Haifa 3200003, Israel

**Keywords:** MoS_2_ monolayers, sulfur vacancies, defect distribution, electronic conductivity, density functional theory, nonequilibrium Green’s function, design of experiments

## Abstract

Molybdenum disulfide (MoS_2_) monolayers are two-dimensional materials belonging to a family of materials called transition metal dichalcogenides which have been widely studied as potential semiconductors for next-generation ingredients in transistor technology. Electronic devices’ performance is largely influenced by defects, and in the case of MoS_2_, the most dominant defects are sulfur vacancies. The correlation between charge transport across MoS_2_ and sulfur vacancies is complex and not trivial, and it is still unclear how the distribution of vacancies influences electronic conductivity. In this study, MoS_2_ monolayers with various sulfur vacancies concentrations and distributions were examined using density functional theory for electronic structure properties, tight-binding (TB) theory to construct the TB Hamiltonian, nonequilibrium Green’s function formalism for transmission function calculations, and Landauer–Büttiker formalism for calculating charge transport. In addition, we employed design of experiments analysis to identify important structural features influencing the calculated current and to fit an empirical model to the results. We found that higher vacancy concentrations lead to a significant increase in electron permeability, with the best results occurring when sulfur vacancies were arranged in lines with alternating presence across both layers. The ability to predict charge transport across MoS_2_ monolayers based on sulfur vacancy distribution can assist in the design of functional materials with desired properties, aiming to selectively apply structural defects.

## Graphical abstract

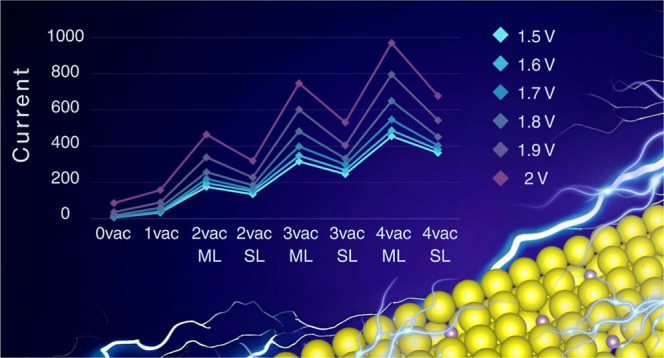

## Introduction

There is a constant drive in the field of microelectronics to develop innovative materials for producing devices, allowing better performance, high reliability, miniaturization, cost-effectiveness, and reduction of the negative impact on the environment by using more efficient and eco-friendly material ingredients. Two-dimensional materials, such as molybdenum disulfide (MoS_2_), are considered to be the next-generation ingredients for electronic applications, like single-layer transistors, photodetectors, sensors, biocompatible devices, and many more.^1–7^ MoS_2_ is a semiconductor material with unique physical and chemical properties, including relatively high carrier mobility, high transparency, flexibility, good mechanical properties, and large surface area, and also unlike its bulk form, the MoS_2_ monolayer has a direct band gap.^8–13^

MoS_2_ belongs to a family of materials called transition metal dichalcogenides (TMDs), materials which are usually composed of three atomic planes: one plane of a transition metal atom sandwiched between two layers of chalcogenides.^14,15^ The most common intrinsic defects among the TMD materials are chalcogen vacancies,^16,17^ and in the case of MoS_2_, it is sulfur vacancies.

The role of the atomic defects in MoS_2_ had been investigated for years, where recent studies show that sulfur vacancies play a critical role in determining electrical performance, by significantly altering the electronic structure of MoS_2_ and reduction of the band gap energy with introducing new electronic states within the band gap.^18,19^ Findings from various studies suggest that introducing new electronic states into the band gap creates alternative pathways for electron movement, enhancing the charge transport properties of MoS_2_. This is achieved through the formation of the n-type doping effect, which increases the concentration of free electrons available for conduction. Functioning as electron reservoirs, the sulfur vacancies enable the accumulation and efficient transport of electrons, further contributing to improved electrical properties.^20–25^ While several studies suggest a positive correlation between electron charge transport and sulfur vacancy concentration, this relationship is not always consistent, and a definitive consensus remains uncertain. According to some published literature, sulfur vacancies can cause both n- and p-type behavior of MoS_2_,^26,27^ while others report that sulfur vacancies contribute to a reduction in electronic current, suggesting that the newly formed midgap states in the original band gap are mostly charge traps,^28,29^ highlighting their dual role in the implications for the conductivity of MoS_2_.

While a correlation between the concentration of sulfur vacancies and electric current has been researched before, the exact nature of this relationship and how it is connected to the spatial distribution of sulfur vacancies remains unclear. This study aims to investigate, for the first time, how both the concentration and spatial distribution of sulfur vacancies in MoS_2_ monolayers affect their electric conduction properties. We focused on examining the impact of the vacancies’ relative arrangement and their presence in either one or both sulfur atomic layers of the MoS_2_ monolayers. Understanding the nature of defects and how their concentration and distribution affect the rate of electron transport through the structure can contribute to developing methods to rationally design electronic materials.

## Methodology

In this work, we investigated the impact of sulfur vacancy concentration and arrangement on the electric current. The methodologies employed in this study were performed in sequence as follows. We started with building a pool of MoS_2_ monolayer structures with various configurations and concentrations of sulfur vacancies. We performed density functional theory (DFT) to calculate their band structures (BS) and other basic properties. The BS were fitted to tight-binding (TB) Hamiltonians, and these fitted Hamiltonians were subsequently used as an input for the current calculation following Landauer–Büttiker formalism. With the electronic structure data at hand, we classified the results according to distinctive features of the structures: vacancy concentration and vacancy arrangement.

### Constructing MoS_2_ Structures

A diversity of different MoS_2_ monolayer structures were generated using VESTA^30^ and CrystalMaker^31^ 3D visualization software. This involved creating models of various sulfur vacancy concentrations and configurations. The MoS_2_ structures are 5 × 5 × 1 supercells, see Figure 1, enabling the formation of various vacancy distributions, with zero to four sulfur vacancies at variable arrangements, where in some cases, the vacancies were placed on a single atomic sulfur plane and in other cases on both. All the models contain 25 Mo and 46–50 sulfur atoms. The 5 × 5 × 1 supercell size for MoS_2_ was chosen because it is a reasonable compromise between the ability to model various sulfur vacancy configurations and concentrations considering the material’s periodicity, while keeping the computationally demanding DFT calculations at a reasonable cost. This study focuses on sulfur vacancy concentrations zero to eight percent, which are in accordance to previously reported experimental vacancy concentrations of up to 15–20%.^32–36^ To eliminate interaction between MoS_2_ monolayers, we added approximately 12 angstrom vacuum space above the monolayers. Overall, we conducted calculations on 45 different atomic structures. The Supporting Information contains all the basic data of the models, including schemes of all structures and the POSCAR files, see “Table S1, Figure S1, and Optimized Structures” chapter.

**Figure 1:**
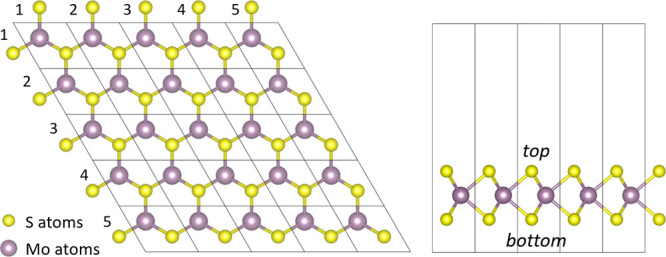
Schematic representation of the MoS_2_ 5 × 5 × 1 supercell divided into primitive cells, from two different view directions, showing a plane of molybdenum atoms (purple) sandwiched between two planes of sulfur atoms (yellow). Generated using VESTA.^30^

To distinguish between the structures, we used the following nomenclature method. The supercells were divided into cells, where each cell represents one primitive unit cell of MoS_2_. The coordinates of each cell in the supercell are introduced by the column and the row indices i and j. MoS_2_ structures have two layers of S atoms and one layer of Mo atoms between them, so S vacancies can occupy top or bottom layers. Therefore, each vacancy gets an index that consists of the vacancy layer and cell coordinates (top/bottom *i* × *j*). For example, see Figure 1, if the vacancy is on the top layer, in the middle of the cell, the index will be top 3 × 3.

The influence of the sulfur vacancies on the electric properties of MoS_2_ structures was analyzed with respect to the following three factors: A. Vacancy concentration. B. Vacancy layer occupancy, which is whether the vacancies are on one atomic layer only or on both. C. Vacancy planar arrangement, specifically distinguishing between line, cluster, or “other” arrangements. As seen in Figure 2a–c, lines and clusters are arrangements with neighboring vacancies, that is, defects which are chemically connected to shared Mo atom or atoms (in the same or the opposite atomic layer).

**Figure 2:**
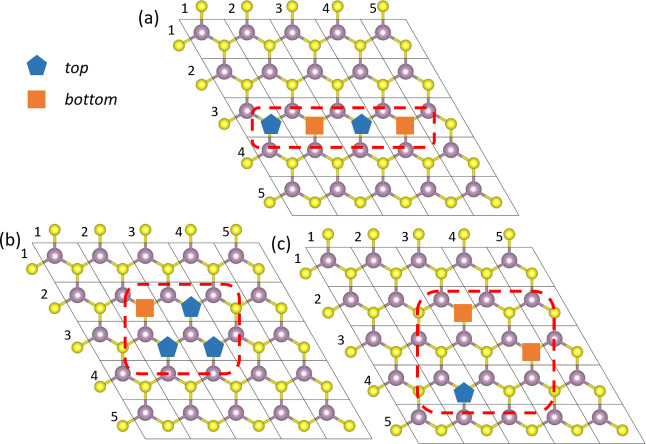
Examples of different vacancy planar arrangement: (a) line defect arrangement with neighboring vacancies alternately placed on top and bottom sulfur layers, (b) cluster defect arrangement with three neighboring vacancies placed on top sulfur layer and one vacancy placed on the bottom, and (c) other arrangement that cannot be classified as line or cluster.

### DFT Calculations

Spin-polarized DFT calculations were performed using the Vienna ab initio simulation package (VASP),^37,38^ version 5.4.4, based on the generalized gradient approximation (GGA) functional of Perdew–Burke–Ernzerhof (PBE),^39^ which is widely used to evaluate the electronic properties of MoS_2_ structures^40–42^ at low computational cost. The core electrons of Mo 1s^2^2s^2^2p^6^3s^2^3p^6^4s^2^3d^10^ and S 1s^2^2s^2^2p^6^ were described using the projector-augmented wave (PAW) method.^43^ The considered valence electrons of Mo are 4p65s^2^4d^4^ (12 electrons) and of S are 3s^2^3p^4^ (6 electrons). The plane wave energy cutoff was set to 400 eV, and the *k*-grid density for the atomic structure relaxations and self-consistent field calculations were determined by KSPACING = 1, centered at the Γ-point. The cutoff energy and the *k*-grid density were determined through benchmark testing. We found that increasing the cutoff energy to values higher than 400 eV and reducing KSPACING below 1 do not significantly affect the total energy (changes are smaller than 1 meV/atom).

The ionic relaxation was performed until the forces on all ions were smaller than 0.01 eV/Å. The employed threshold for electronic minimization was 1 × 10^–6^ eV. For the BS calculations, we used *k*-points that describe the high symmetry lines along the first Brillouin zone (BZ), produced by SeeK-path,^44,45^ see KPOINTS in the Supporting Information, Figure S2. The calculated charge densities were used to obtain the total energy of a system, charge distribution, density of states (DOS), electronic BS, local electron potentials, and vacancy formation energy.

### Charge Transport Calculation

The electron charge transport of MoS_2_ monolayers with sulfur vacancies was calculated using a multistep computational approach, combining TB theory for the construction of a TB Hamiltonian fitted to the DFT BS, the nonequilibrium Green’s function (NEGF) formalism for transmission function calculations, and the Landauer–Büttiker formalism to compute the electron charge transport versus bias.^46–49^ These calculation procedures were performed using the python code that was implemented within our research group and was described and published by Elbaz et al.^48^ Below we describe the methods employed for charge transport calculations in this work. Details about the main Python scripts and workflow used to calculate electron charge transport can be found in the Supporting Information section titled “Workflow for Charge Transport Calculation”.

The system used in this research to calculate electron charge transport consists of two semi-infinite electrodes connected to a central scattering region, as shown in Figure 3a. Both the scattering region and the electrodes are composed of the same MoS_2_ supercells with various defect arrangements, whose electronic structures were first established through DFT calculations done in this work. The scattering region itself consists of four layers of MoS_2_ supercells layered side by side. The choice of four supercells represents a pragmatic trade-off: balancing the need to simulate a large enough region for reliable long-range charge transport affected by variations in sulfur vacancy configurations, while also maintaining reasonable computational costs. The distance we use was found sufficient for calculating current through various ceramic materials.^48^ Since electrons in semiconductors like MoS_2_ tend to scatter over short distances,^50,51^ a scattering region of this length captures enough scattering events and allows electrons to travel far enough to experience the material’s periodicity.^47^

**Figure 3:**
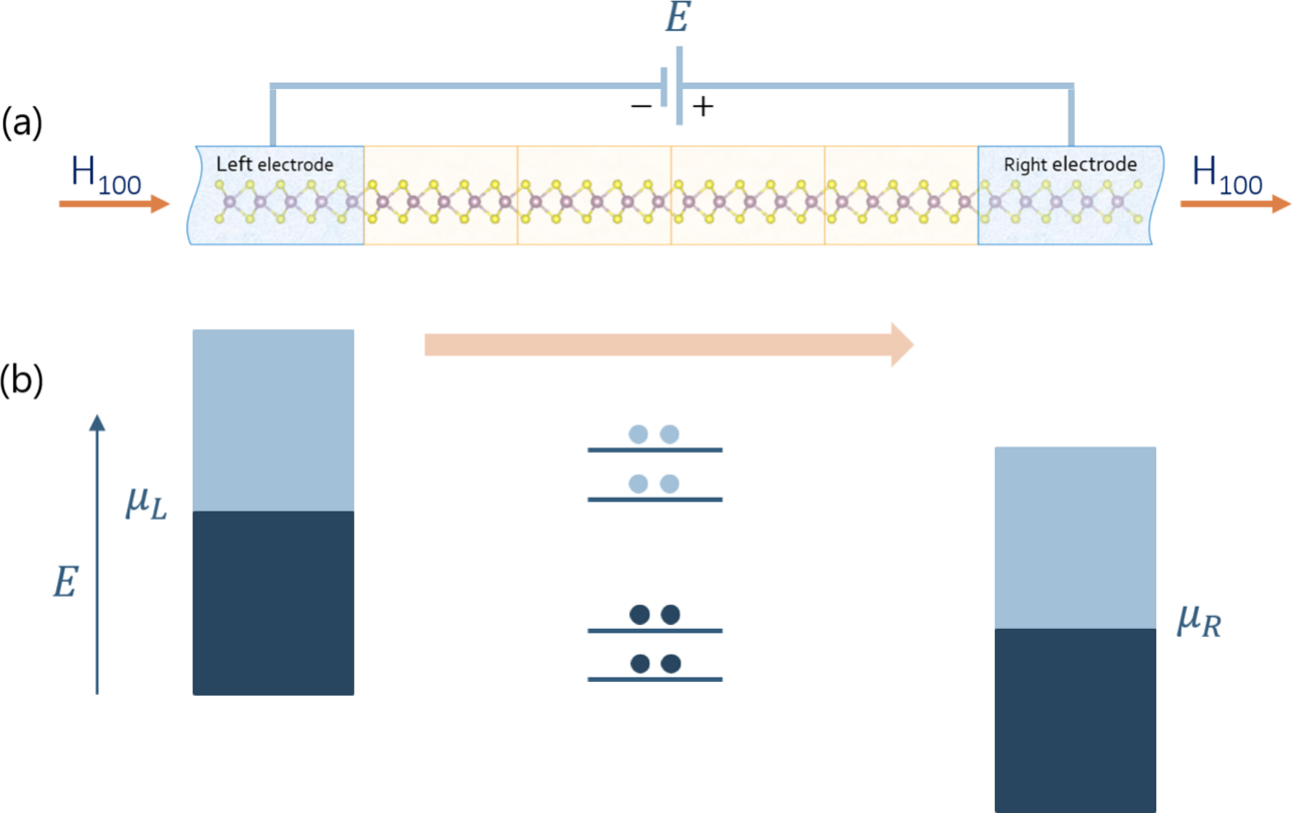
One-dimensional charge transport: (a) schematic sketch of the tested system consists of the scattering region and two electrodes. The scattering region is four 5 × 5 × 1 supercells layered together side by side in the [100] direction. (b) BS representation in the left and right contacts.

### TB Hamiltonians Construction

The initial step in the charge transport calculation process involved fitting the DFT BSs into TB Hamiltonians, which are useful for effectively interpolating the electronic structures of solids. TB Hamiltonians describe the system energy in real space with a manageable number of parameters, leading to a significant reduction in the computational cost of the following NEGF calculations.^49,50,52–58^ The TB Hamiltonian as a function of the k vector is expressed by eq 1.

1 $$H_{TB}\left(k\right)=\sum_{R}e^{ik\cdot R}H_{R}$$

Here, *R* represents the real-space lattice vector, and *H*_*R*_ expresses the corresponding real-space TB Hamiltonian matrices. The set of lattice vectors in this TB approach limits the interaction range to the nearby sites. This study focuses on one-dimensional charge transport and considers only the lattice vectors *R* = (0,0,0) and (1,0,0). *H*_00_ represents the real-space TB Hamiltonian matrix for the unit cell, while *H*_100_ corresponds to the Hamiltonian matrix that couples with adjacent unit cells along the (1,0,0) direction.

The construction of the TB Hamiltonians involved a fitting procedure to reproduce the electronic BS obtained from the DFT calculations. In this study, a machine learning approach is implemented to adjust the *H*_*R*_ matrices iteratively until the computed BSs align with the BSs from the DFT calculations. This method, unlike traditional empirical fitting or first-principles methods, does not directly employ atomic orbitals or projections. Here, the basis functions are implicitly defined by the parametrization of the TB Hamiltonian matrices and the number of fitted bands, rather than by an explicit set of atomic orbitals.

The energy range within a specific window of interest for charge transport calculations was limited by the number of bands from the DFT BS included in the TB Hamiltonian fitting process. Specifically, we included 100 bands below and 100 bands above the valence band maximum (VBM) from the DFT data since this amount was sufficient to capture the rise in the current resulting from states appearing in the projected DOSs plot.

The TB fitting process starts with the creation of an initial TB Hamiltonian matrix where the size of the matrix is determined by the number of fitted bands. The elements of this initial matrix are normally distributed random values.^48^ The TB BS was determined from the eigenvalues, denoted as ε_*n*,*k*_^*TB*^, which represent the energy of the *n*-th band at a specific *k*-vector, as shown in eq 2.

2 $$H_{k}^{TB}\psi_{n,k}^{TB}=\varepsilon_{n,k}^{TB}\psi_{n,k}^{TB}$$

The TB BSs were fitted to the DFT BSs by minimizing the mean-squared error loss (MSE) between the TB and DFT eigenvalues using a back-propagation algorithm. The MSE is defined by eq 3.

3 $$MSE=\frac{1}{N}\frac{1}{N_{k}}\sum_{i=1}^{N}\sum_{k}{\left(\varepsilon_{i,k}^{TB}-\varepsilon_{i,k}^{DFT}\right)}^{2}$$

The MSE was calculated across all bands (*N*) and *k*-points (*N*_*k*_). The elements in the TB Hamiltonian matrices (*H* ^*R*^) were iteratively updated using the Adam gradient descent algorithm^48,59^ to minimize the MSE according to eq 4, where *l* defines a single iteration cycle of the learning process and α represents the learning rate.

4 $$H_{l+1}^{R}=H_{l}^{R}-\alpha \frac{\partial MSE}{\partial H_{l}^{R}}$$

See the Supporting Information section titled “Workflow for Charge Transport Calculation” for more details about the machine learning and back-propagation parameters used to fit the real-space TB Hamiltonians.

Since our system is composed of principal layers, each representing a single unit cell of the MoS_2_ supercell, we can employ the TB Hamiltonians, which describe the electronic structure of the supercells, to define the block matrix of a single layer as *H*_00_. Given that the electrodes and the scattering region are composed of the same blocks, we assume that *H*_layer_ = *H*_00_ = *H*_*R*_ = *H*_*L*_. The coupling matrices between adjacent layers are defined by the block matrix *H*_layer coupling_ = *H*_100_ = *H*_*R*,*R*_ = *H*_*L*,*L*_.

Following, the Hamiltonian for the scattering region is expressed as

5 $$H_{scattering}=\left(\begin{array}{llll} H_{00} & H_{100} & 0 & 0\\ H_{100} & H_{00} & H_{100} & 0\\ 0 & H_{100} & H_{00} & H_{100}\\ 0 & 0 & H_{100} & H_{00} \end{array}\right)$$

and the full Hamiltonian representing the total system can be formulated as follows

6 $$H=\left(\begin{array}{lllll} \ddots & \ddots & 0 & 0 & 0\\ \ddots & H_{L} & H_{L,L} & 0 & 0\\ 0 & H_{L,L}^{†} & H_{scattering} & H_{R,R} & 0\\ 0 & 0 & H_{R,R}^{†} & H_{R} & \ddots \\ 0 & 0 & 0 & \ddots & \ddots \end{array}\right)$$

After the TB Hamiltonians are defined, they are used as inputs for quantum transport calculations.

### Quantum Transport Calculations

The following step in the process was to employ the NEGF by calculating the transmission function (using the TB Hamiltonian) and Landauer–Büttiker formalism to perform quantum transport calculations as described below.

The Landauer–Büttiker formalism is commonly used to understand the conduction properties of materials placed in a scattering region between two electrodes that act as a source and drain of electrons. The right electrode has a chemical potential μ_*R*_ and the left μ_*L*_, while the potential formed between the electrodes, within the scattering region, is the bias that is determined by the difference in chemical potentials between the left and right electrodes and is given by μ_*L*_ – μ_*R*_, see Figure 3b.

This approach assumes coherent electron transport, where electrons move through the scattering region without undergoing inelastic scattering events (e.g., electron–phonon interactions).^47,50,51,60^ In MoS_2_ monolayers, the typical room-temperature electron mobility ranges from 20 to 200 cm^2^/(V s), leading to an estimated mean free path of electrons, before inelastic scattering happens, in the range of nanometer scale.^14,61–63^ Given that the distance between the electrodes in our setup is approximately 6 nm (four unit cells), we assume coherent electron transport. For larger systems or those with increased energy dissipation, decoherence models beyond the Landauer–Büttiker formalism may be required.

While the simplest form of the Landauer–Büttiker formula is limited to low bias, it can be extended to finite biases when combined with advanced methods such as NEGFs. NEGF provides the transmission function necessary for the Landauer–Büttiker formalism at finite bias, as shown below, allowing for the calculation of full current–voltage characteristics beyond the low-bias regime.^47,60,64,65^

The calculation of the electric current under an applied bias from one electrode to another through the scattering region is expressed by the Landauer–Büttiker formula, given by eq 7

7 $$I=\frac{2e}{h}\int_{-\infty }^{+\infty }T\left(E\right)\left[f\left(E-\mu_{L}\right)-f\left(E-\mu_{R}\right)\right]dE$$

where *T*(*E*) is the transmission function of an electron from one electrode to another through the scattering region and *f*(*E* – μ) is the Fermi–Dirac distribution, expressing the probability of an electron to have energy *E*. The Fermi–Dirac distribution is given in eq 8.

8 $$f\left(E-\mu \right)=\frac{1}{\exp\left(\left(E-\mu \right)/k_{B}T\right)+1}$$

where *k*_B_ is Boltzmann’s constant, *T* is the temperature, taken as 300 K, and μ is the chemical potential. Only the electron states between μ_*L*_ and μ_*R*_ (μ_*L*_ > *E* > μ_*R*_) contribute to the current flow from the left electrode to the right.

The transmission function *T*(*E*) is computed using the NEGF formalism and is presented by eq 9.

9 $$T\left(E\right)=trace\left[{\mathrm{\Gamma ̂}}_{R}\left(E\right){Ĝ}_{S}\left(E\right){\mathrm{\Gamma ̂}}_{L}\left(E\right){Ĝ}_{S}^{†}\left(E\right)\right]$$

where ${Ĝ}_{S}\left(E\right)$ is Green’s function term of the central scattering region and ${\mathrm{\Gamma ̂}}_{R/L}\left(E\right)$ is the broadening matrix. Equation 10 describes the Green’s function of the scattering region ${Ĝ}_{S}\left(E\right)$ .^47^

10 $${Ĝ}_{S}\left(E\right)={\left[EI-H_{scattering}-\Sigma_{L}\left(E\right)-\Sigma_{R}\left(E\right)\right]}^{-1}$$

where *E* is the energy, *I* is the identity matrix, *H*_scattering_ is the TB Hamiltonian of the scattering region, and Σ_*R*/*L*_(*E*) is the self-energy matrix of the connected electrodes.

The broadening matrices, ${\mathrm{\Gamma ̂}}_{R/L}\left(E\right)$ , represent the coupling between the electrodes and the scattering region at a specific energy *E* and are calculated using eq 11.^47,66^

11 $${\mathrm{\Gamma ̂}}_{R/L}\left(E\right)=i\left(\Sigma_{R/L}\left(E\right)-\Sigma_{R/L}^{†}\left(E\right)\right)$$

where Σ_*R*/*L*_ represent the self-energy matrices of the electrodes, right (Σ_*R*_) and left (Σ_*L*_), and Σ_*R*/*L*_^†^ is its conjugate transpose. These matrices accounting for how the semi-infinite electrodes influence the scattering region.^60,67^ The self-energy matrices are determined by eq 12.

12 $$\Sigma_{R/L}=H_{R/L,R/L}^{†}g_{R/L}H_{R/L,R/L}$$

where *H*_*R*/*L*,*R*/*L*_ represents the TB Hamiltonian matrices coupling the scattering region to the electrodes (*H*_*L*,*L*_ to the left electrode and *H*_*R*,*R*_ to the right) and g_*R*/*L*_ is the surface Green’s function of the electrodes (g_*L*_ of the left electrode and g_*R*_ of the right).

The surface Green’s functions (g_*L*_ and g_*R*_) are calculated using a recursive algorithm. This method iteratively determines the surface Green’s functions and self-energies by increasing the number of electrode layers until convergence is reached. The recursive formula for the g_*R*/*L*_ calculation is given by expression [Disp-formula eq13].

13 $$g_{R/L}^{\left(n+1\right)}={\left[H_{R/L}-H_{R/L,R/L}^{†}g_{R/L}^{\left(n\right)}H_{R/L,R/L}\right]}^{-1}$$

The iterative procedure begins with an initial guess of g_*R*/*L*_^(*n*)^ = *H*_*R*/*L*,*R*/*L*_ and continues until the following criterion is fulfilled

14 $${\left(g_{R/L}^{\left(n-1\right)}-g_{R/L}^{\left(n\right)}\right)}_{ij}^{2}\leq \delta^{2}$$

The δ parameter serves as the stopping criterion for the recursive algorithm.^48,49^

### Design of Experiments and Data Analysis

We used the well-established design of experiments (DOE) method^68^ to analyze how the charge transport across MoS_2_ monolayers in the various studied structures is affected by changing the following independent factors: the applied bias, sulfur vacancy concentration, layer occupancy, and vacancy planar arrangement. DOE is a powerful statistical method that allows the empirical examination of the relationships between multiple predefined inputs (variables/features/factors) and measured/calculated outputs (responses/results) that encompass the system behavior and dependencies in a region of interest, using relatively few time-consuming calculations.

A commonly used approach to check the sensitivity of responses to various factors is to carry out “one at a time” experiments, where in each experiment, all factors except one are kept at fixed values (levels). However, changing only one variable at a time may lead to missing important interactions among the examined factors. The advantage of the DOE method over “one at the time” analysis of the factors is that it enables the examination of interactions between the factors and the responses. An interaction between two factors refers to a case where the influence of one factor on the response (here, current) depends on the level of the other one. A full factorial (FF) design analysis was employed with the factors and the levels (continuous or categorical factors values) summarized in Table 1.

**Table 1:** Factors and Levels for Design of Experiments Analysis

factors	levels
bias [V]	1.5, 1.6, 1.7, 1.8, 1.9, 2
defects (vacancies) concentration [%]	0%, 2%, 4%, 6%, 8%
layer occupancy	one layer, two layers
defects (vacancies) planar arrangement	line, cluster

The full factorial 5 × 2 × 2 design matrix can be found in the Supporting Information, Table S6, including the MoS_2_ structures implemented within the model. This design was used to investigate the effects of defects (vacancies) concentration (five levels), layer occupancy (two levels), and defects (vacancies) planar arrangement (two levels) on the electric current. For each pattern in the design, the current was calculated for six bias values between 1.5 and 2 V.

Some structures with lower defect concentrations were used several times to describe the initial stages of continuous development toward higher defect concentrations. For example, pristine structure and structure with one vacancy are included four times each to describe 0% and 2% of defect concentration in line/cluster plane arrangements and single/mixed layer occupancies. In the case of 4% concentration of defects (two missing S atoms), we included in the model the same structures twice to describe line and cluster planes arrangements with two neighbor vacancies: one structure describes both line and cluster arrangements with vacancies on a single layer and two other structures describe both line and cluster arrangements where vacancies are on both sulfur atomic layers.

Statistical analysis was performed using JMP Pro 18 software.^69^ To fit the FF design model, we employed the standard least squares method due to its robustness and ability to handle both continuous and categorical factors. Analysis of Variance (ANOVA) was performed to determine the statistical significance of the factors and the interactions between them. A significance level of α = 0.05 was used for all statistical tests. The response variable used in the FF design is the square root of the calculated current. The square root was used to prevent negative values.

### Vacancy Formation Energy Calculation

Sulfur vacancy formation energies were calculated for all structures containing S vacancies using the following eq 15.^70–73^

15 $$E_{formation}=E_{total}^{\left({Mo}_{25}S_{75-n}\right)}-E_{total}^{\left({Mo}_{25}S_{75}\right)}+n\mu_{S}$$

where $E_{total}^{\left({Mo}_{25}S_{75-n}\right)}$ is the total energy of the 5 × 5 × 1 supercell which contains n sulfur vacancies, $E_{total}^{\left({Mo}_{25}S_{75}\right)}$ is the total energy of the pristine MoS_2_ supercell, and *n*μ_S_ is the chemical potential of the isolated sulfur atom (μ_S_) multiplied by the amount of sulfur vacancies (*n*). The sulfur chemical potential, μ_S_, was determined by the DFT-calculated energy of the S_8_ molecule under vacuum divided by eight, the number of atoms in the molecule. The POSCAR file of the S_8_ molecule is provided in the Supporting Information, under the Optimized Structures section. The average vacancy formation energy per structure is its total formation energy divided by the number of vacancies: ${\frac{1}{n}E}_{formation}$ .

### Ionization Potential Calculation

The ionization potential (IP), which is the minimum energy needed to remove an electron from a material to the vacuum outside the structure surface, was calculated per structure using the common definition in eq 16.

16 $$IP=E_{vacuum}-E_{loel}$$

where *E*_vacuum_ is the vacuum potential, the electrostatic potential away from the surface, and *E*_loel_ is the last occupied energy level. The vacuum potentials were extracted from the potential projection in the *z* direction, the maximum value, and the last occupied energy levels from the self-consistent electronic structure calculations.

## Results and Discussion

Our research examined how the sulfur vacancy arrangement affects the charge mobility in MoS_2_ monolayers. The following section presents first the current calculation results of the monolayers. Next, we present statistical analysis results of the calculated data and subsequently the electronic and geometrical properties of the examined defected MoS_2_ monolayers.

### Current Calculations

First, the current calculations for different MoS_2_ monolayers with various sulfur vacancy concentrations were examined. The results presented in Figure 4 indicate that the current increases with bias and defects concentration. These observations strengthen the previous reports demonstrating the contribution of sulfur vacancies to the electric current in MoS_2_.^21,22,74^ Although a positive correlation between vacancy concentration and the current was observed, the significant variability in the calculated results suggests that additional factors contribute to the overall electric charge transport. In the Supporting Information, Figure S8 presents side-by-side BS, DOS, *T*(*E*), and *IV* plots for three different structures: one with zero vacancies, one with a single vacancy, and one with two vacancies.

**Figure 4:**
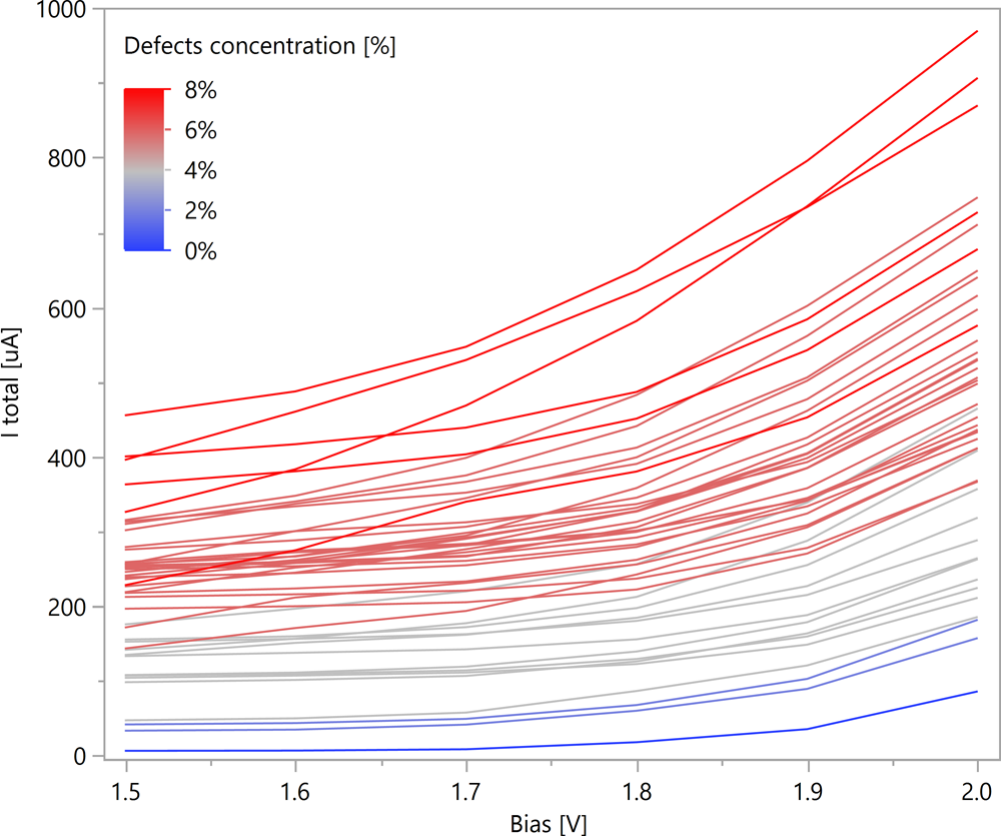
Current vs bias plots at *T* = 300 K of MoS_2_ 5 × 5 × 1 structures with different concentrations and arrangements of sulfur vacancies. The results of *I*–*V* calculations for all structures are provided in the Supporting Information, Table S5, and Figure S9.

To reveal the influence of the three additional factors (defects concentration, layer occupancy, and defects planar arrangement) on the charge transport, we use the variability chart, presented in Figure 5, for graphical representation of the variation in the electric current. The ranges of total current are grouped by defects concentration, arrangement, and applied bias. This division into groups and subgroups helps us see previously unseen effects. For instance, the chart clearly shows that for cluster and line defects arrangements, the current is higher when the vacancies are located on both sulfur atomic layers rather than on one layer only. A similar, although less pronounced, effect is observed for structures with two neighbor vacancies, suggesting that the effect is stronger at higher vacancies concentrations since an introduction of an additional sulfur vacancy to the two-neighbor-defects structure will lead to the formation of line or cluster arrangements.

**Figure 5:**
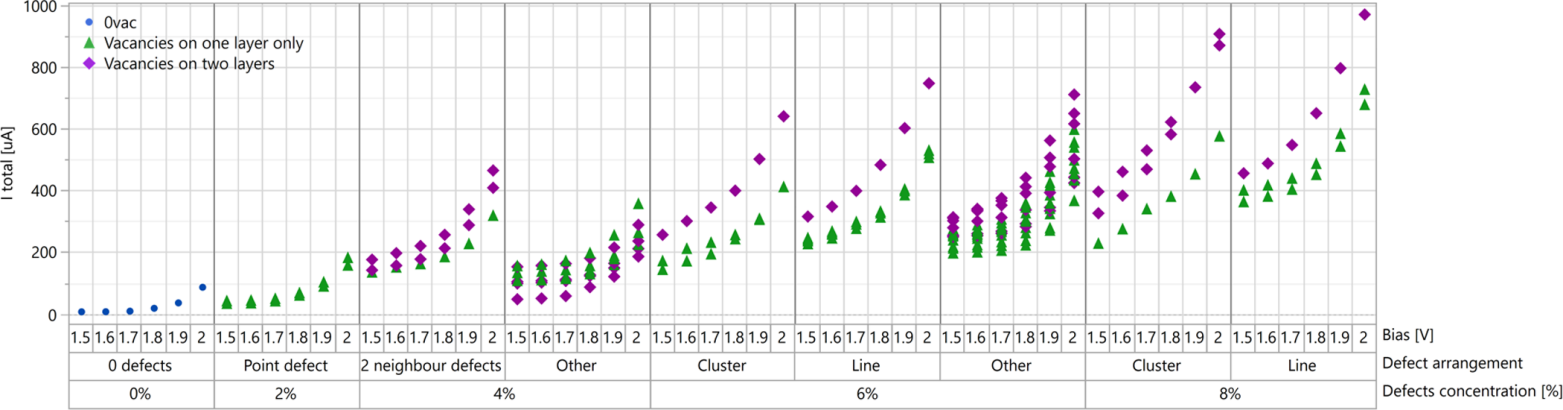
Variability chart presenting the range of total current grouped in the following hierarchy: first by defects concentration, followed by defects arrangement, and then by the applied bias. Green triangles and purple rhombuses represent structures with vacancies in one or both sulfur layers, respectively. Blue dots represent pristine MoS_2_.

We learn from the variability chart that the impact of layer occupancy, which is spreading vacancies on both sulfur layers of MoS_2_ monolayers compared to one layer only, on the electric current is more significant when the sulfur vacancies appear in close proximity to each other, as demonstrated for the case of 4% (two neighbor defects compared to “other”). When vacancies are dispersed over a larger distance, the contribution of the layer occupancy effect is less pronounced. Conversely, increased vacancy concentration results in a higher density of defects within the monolayers, so they affect each other, which makes the layer occupancy effect stronger as we can see by comparing the following groups in Figure 5: “2 neighbor defects” with “line” and “cluster” at higher defect concentration and “other” at 4% with “other” at 6% concentrations.

Furthermore, a tendency toward higher current was observed for line vacancy arrangements compared to cluster arrangements, particularly for larger vacancies concentration.

### Statistical Analysis

To gain deeper insight into the factors influencing the current, we employed a DOE approach, as described in the Methodology section. We found that the primary factors which distinctly influence the current are vacancy concentration, applied bias, and layer occupancy as summarized in Table 2. These three parameters are the key factors listed at the top of the effect summary list with the highest Logworth values. The layer occupancy, which indicates the spreading of vacancies either in both sulfur layers or in a single one, appears to have a surprisingly large effect on the current. Defects concentration factor appears multiple times, both as a main effect and in higher order effects. When the defect concentration appears alone, it represents the individual impact of this factor on the current. When combined with other factors, it represents interactions, the combined effect of defect concentration, and the other factors on the current. Additionally, based on the *P* Value, defect planar arrangement in lines compared to clusters defects present a significant effect.

**Table 2:**
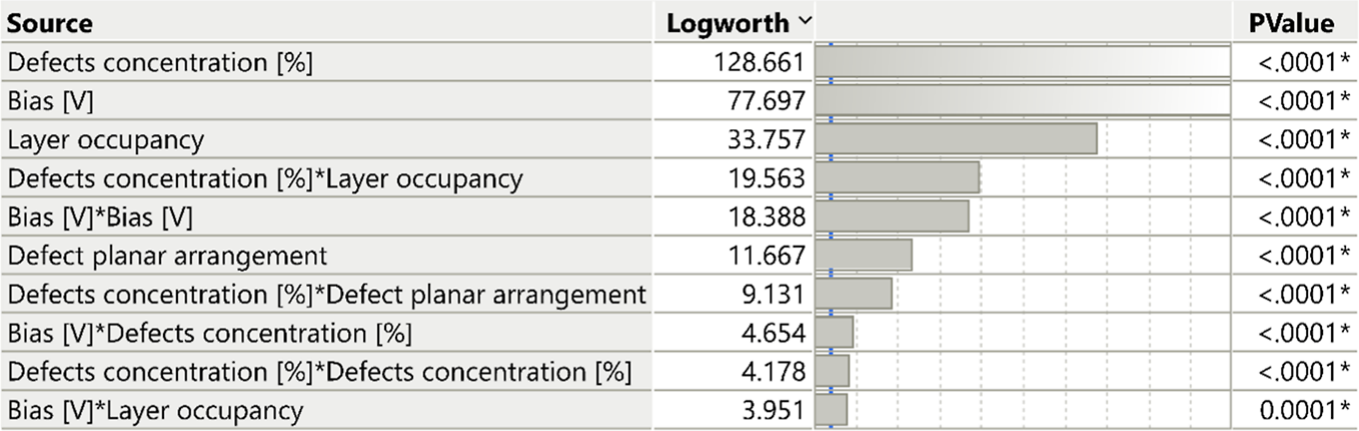
FF DOE Main Effect and Interaction Results Summary for the Electric Current through MoS_2_ Monolayers as a Function of the Four Studied Factors (See Table 1)­^a^

a Logworth is defined as −log_10_(*P* value), a higher Logworth value suggests a more significant impact.

The most significant interactions were observed between vacancy concentration and layer occupancy, defect planar arrangement, and bias. We also observe an interaction between the bias and layer occupancy. The interaction suggests that the effect of one factor on the current is dependent on the other ones. The two interactions, between vacancy concentration and layer occupancy, and between vacancy concentration and defects planar arrangement, are reasonable since at low vacancies concentrations (0% and 2%), the effect of layer occupancy does not exist and because the defects planar arrangements are forming substantially with an increase in their concentration. An additional discussion regarding the interactions in the studied structures is presented below. There are also significant second-order effects of the bias and defect concentration, which indicates that the relationships between the current and these factors are parabolic in the studied range. These dependencies are present in the current calculations shown in Figure 5 and are included in the fitted model, as expected.

The quality of the fitted FF design model based on a comparison of the predicted electric current to the calculated results for the various structures is presented in Figure 6. *R* ^2^ = 0.98 indicates a good fit to the calculated current data, and the *P*-value <0.0001 is small enough to indicate highly convincing significance, rejecting the null hypothesis of chance. In Figure 7, the FF design model current prediction values are compared to the calculated ones. A very good agreement between the model’s prediction and the calculated current values is obtained in the studied range of all factors: bias, defect concentration, defect arrangement, and layer occupancy.

**Figure 6:**
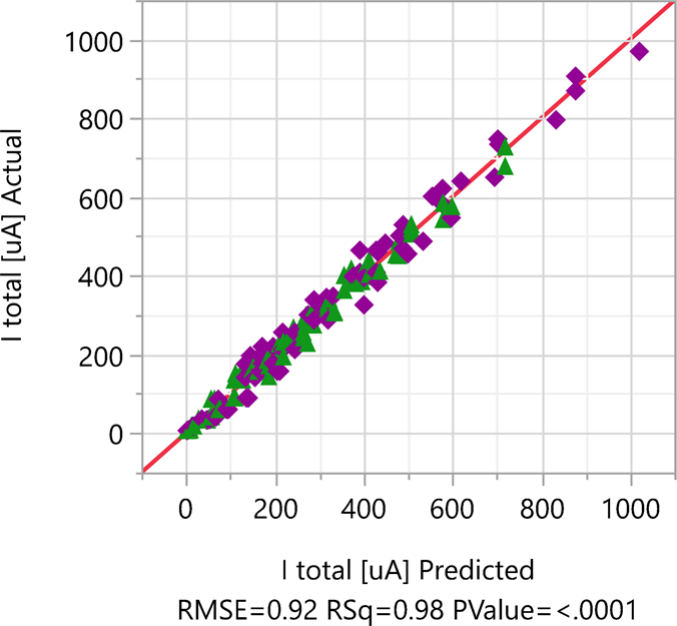
Calculated current versus FF DOE design predicted current plot presents the quality of the fitted model.

**Figure 7:**
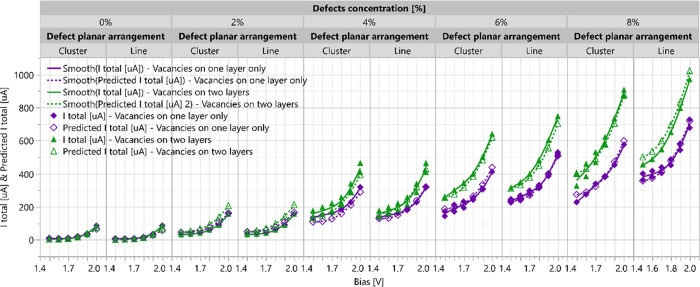
Comparison between the DOE-fitted FF model and the calculated current results. This chart illustrates the range of predicted and calculated current values grouped in the following hierarchy: first by defects concentration, followed by defects arrangement, and then by the applied bias. Green triangles and purple rhombuses represent structures with vacancies in one or both sulfur layers, respectively. The solid lines represent the fitted smooth curve of the calculated current, while the dashed lines represent the fitted smooth curve of the predicted current.

See the predicted and calculated current values versus the applied bias in Figure S10 of the Supporting Information.

Prediction profiler is a commonly used tool in DOE analysis for learning how the effect (level) of one factor changes the effect of other factors on the response according to the fitted model. The prediction profiler of the current versus the four factors is shown in Figure 8. Please refer to the Supporting Information for an interactive report produced by JMP software, which allows the adjustment of factor values in the prediction profiler and observation of the corresponding changes in the total current.

**Figure 8:**
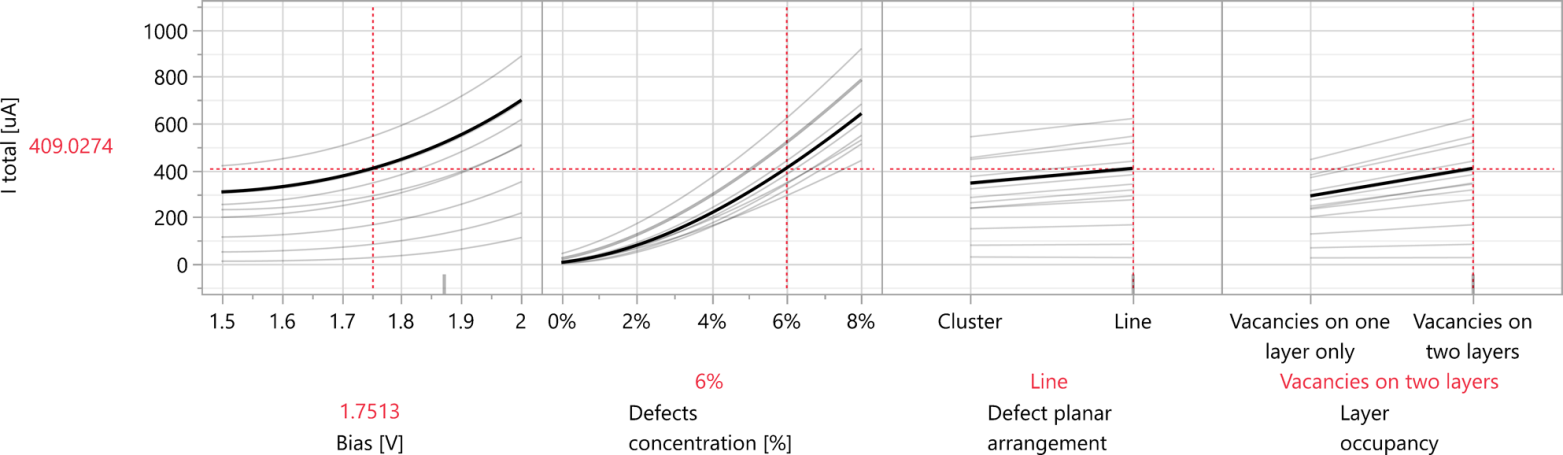
Prediction profiler of the current versus the four factors: the applied bias, defects concentration, defect planar arrangement, and layer occupancy. The bold black lines are the calculated current curves per factor, obtained at constant values of the other factors, indicated in red. The light gray lines represent interaction curves determined by combinations with the other factors.

The results confirm the expected positive correlation between the bias and electric current. Furthermore, the current–bias curve exhibits steeper growth as the concentration of vacancies increases. Notably, the vacancy concentration has the highest influence on the current, leading to a significant increase in current as the vacancy concentration is elevated. As seen in the variability chart before, there is a higher current for line over cluster defects and for distribution of the vacancies within both sulfur layers. Also, there is an interaction between defect concentrations and both defect planar arrangement and layers occupancy since the growth of the current versus defect planar arrangement and the layer occupancy increases at higher defect concentrations (see more details on the interaction profile plots in Figure S11 of the Supporting Information). Based on the prediction plot, the maximum current is observed for line defects when sulfur vacancies are present on both atomic layers at elevated concentrations and bias voltages.

### Electronic and Geometrical Properties

To more clearly elucidate the impact of the layer occupancy factor, we conduct a side-by-side comparison of line and cluster vacancy arrangements. Figure 9a,b presents the current calculations, ionization potential, band gap energies, and Mo–S bond length distributions of MoS_2_ monolayer structures with an increasing concentration of defects, when structures with defects on one layer and on both (mixed) layers are shown alternately side by side. As we look at the same vacancy arrangements and varied layer occupancy, the results reveal that the current notably increases when the vacancies are scattered between the layers. An opposite trend, which also reinforces the improvement in electric conduction, was observed for the ionization potential, the band gap energy, and the average Mo–S bond length; all three of them decrease once the vacancies are located on both layers compared to a single layer. Furthermore, we observed a decrease not only in the average Mo–S bond length but also in the distribution of the bond’s length when the vacancies are located on both layers. Please find in the Supporting Information, in Figure S6, box plot showing the variability and central tendency of Mo–S bond length for various MoS_2_ structures.

**Figure 9:**
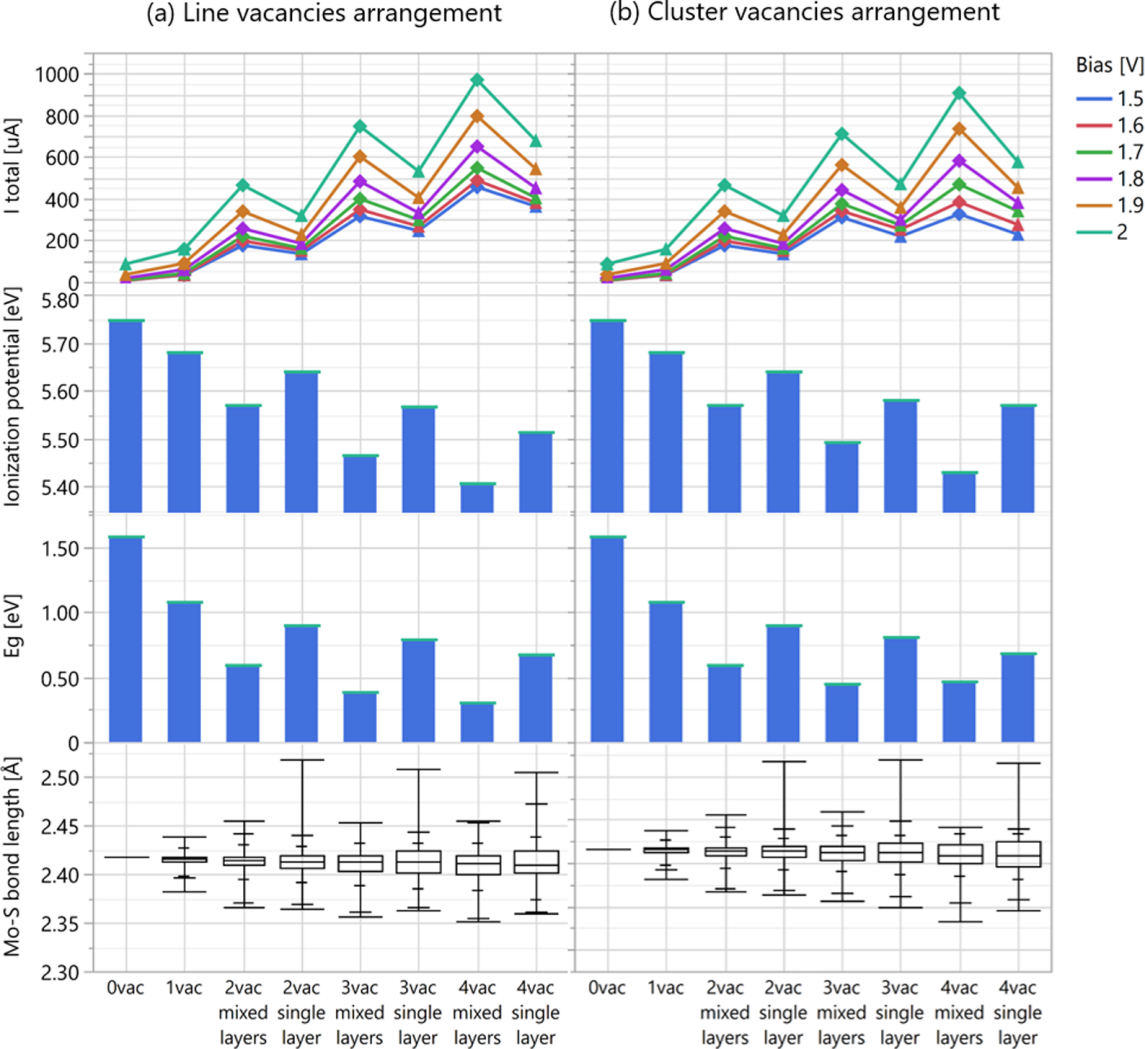
Plots (a) and (b) present the current calculations, ionization potentials, band gap energies, and Mo–S bond length distributions of MoS_2_ monolayer structures with line and cluster defect arrangements, respectively. The horizontal axis represents increasing sulfur vacancies, alternating between double (mixed) and single layers occupancy.

Upon separate examination of mixed and single-layer occupancy for structures with line and cluster vacancy arrangement, we observe the following trends with increasing sulfur vacancies concentration: increasing current and decreasing ionization potential, band gap energy, and average Mo–S bond length. Hence, decreases in ionization potential, band gap energy, and bond length parameters are concomitant with an increase in the electric current. Please find in the Supporting Information, Table S2, the summary of DFT-calculated energies (total, band gap, and vacancy formation) for various MoS_2_ monolayers, and in Figure S3, a comparison of band gap energies, ionization potential, and mean Mo–S bond length as a function of vacancies concentration is presented for defects with vacancies located on a single sulfur layer versus those with vacancies on both layers.

The observed decrease in ionization potential can be attributed to the increase in surface dipole moment, which is positively correlated with an increase in sulfur vacancy concentration in MoS_2_ monolayers. There is an established relationship between ionization potential and surface charge, when more positive charges lead to lower ionization potential.^75–78^ The absence of sulfur atoms results in a local reduction of charge, which, in turn, leads to a positively charged surface and a decrease in ionization potential. Please find in the Supporting Information, Figure S7, the charge difference images of structures with same sulfur vacancy concentrations and different vacancy arrangements and see that the surface charge distribution can vary for samples with the same vacancy concentration depending on their location, which can significantly influence the ionization potential value.

Following the trends observed for the average Mo–S bond length, we further analyzed the geometric structures of the monolayers. Interestingly, when vacancies appeared on a single atomic layer, nearby sulfur atoms bend toward the vacancies and distort the planar structure of all three atomic layers. When the vacancies appear on both layers, the structure remains relatively planar; Figure 10a,b presents examples of the top view of six MoS_2_ monolayers with different vacancy concentrations and arrangements. The Mo–S bonds are colored according to their length compared to the mean Mo–S bond length in the pristine structure. The colors of the bonds visually show that their lengths are more diverse when the vacancies are located on the same layer due to the stronger distortion created. The Supporting Information contains tables summarizing data for all calculated structures: Table S3 the data of Mo–S bond lengths and Table S4 the data of structure parameters.

**Figure 10:**
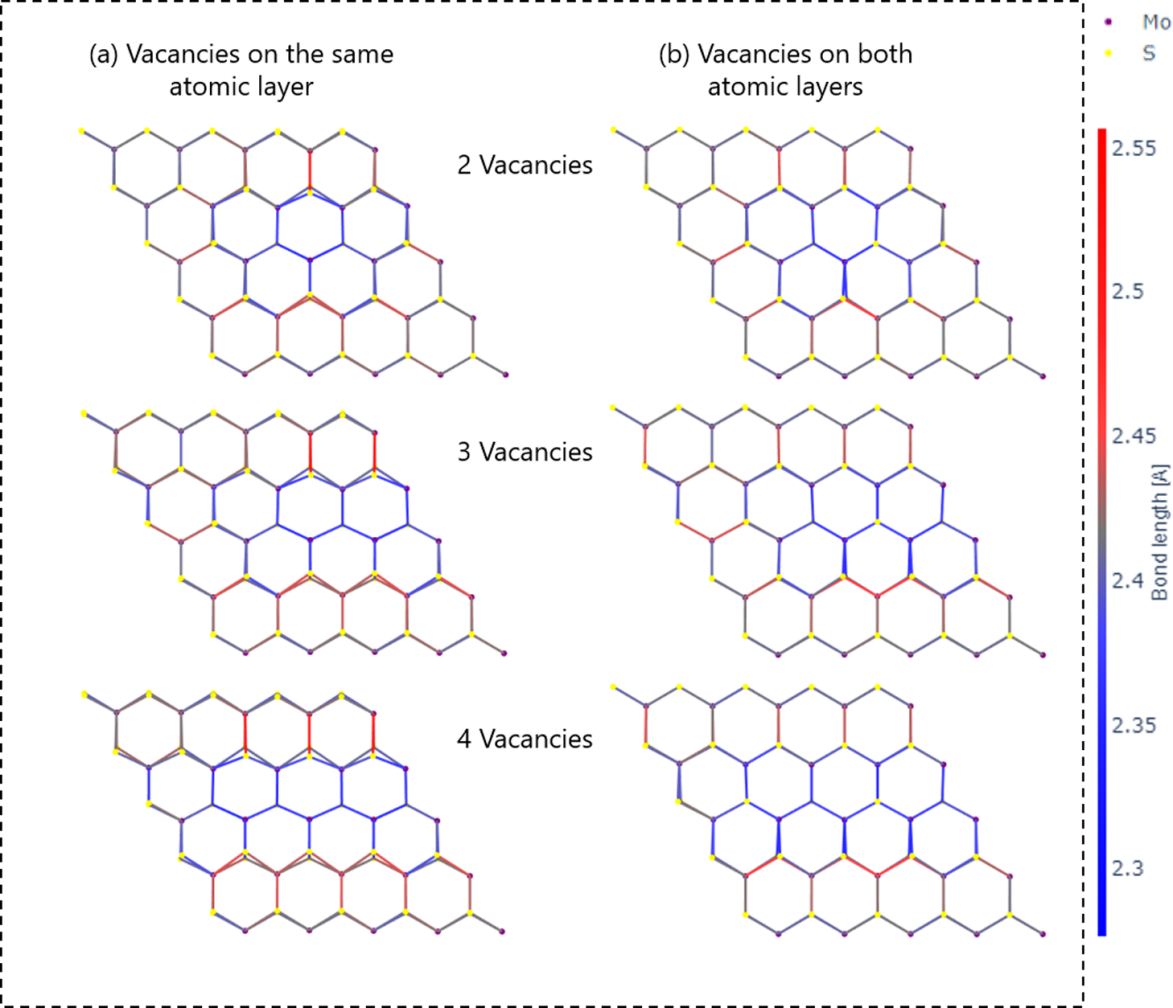
The top view of MoS_2_ monolayers with different length line vacancy arrangements. The Mo–S bonds are colored according to their length relative to the mean Mo–S bond length in the pristine structure.

The deformation of the MoS_2_ monolayer structure leads to a deviation from the planarity of the atomic layer of molybdenum atoms. Examples of the atomic layer distortions as a function of layer occupancy are shown in Figure 11a–d, presenting four different MoS_2_ monolayers with four vacancies at different layer occupancies, either with a line or cluster arrangement. An improvement in the planar deformity is seen in both line and cluster cases, (b) compared to (a) and (d) compared to (c).

**Figure 11:**
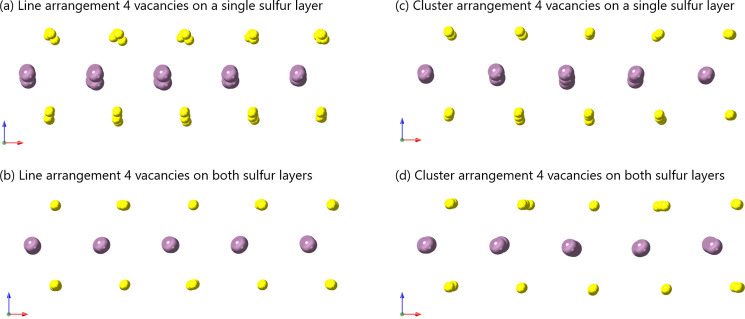
MoS_2_ monolayers with four vacancies, (a) line arranged vacancies on the same layer, (b) line arranged vacancies placed on top and bottom layers alternately, (c) cluster-arranged vacancies on the same layer, (d) cluster-arranged vacancies placed on the top and bottom layers alternately, where the view direction is [010].

The DFT calculations reveal, as we see in Figure 12a–d, that the electronic structure is significantly affected not only by the vacancies’ concentration but also by the vacancies’ positions in relation to each other. Vacancies in the same concentration induce additional electronic midgap states within the band gap at different energies, depending on their occupation sites in the structure. Most states close to the valence and conduction bands belong to the d orbitals of Mo atoms, and the majority of the newly generated states in the original band gap, resulting from the sulfur vacancies present, also have dominating d orbitals of Mo atoms, although there is a considerable presence of states related to the p orbitals of sulfur atoms. This study may be extended in the future to include calculations using the GW approximation, which could improve the accuracy of the BS estimation. For total DOS plots of all models, see Figure S5 in the Supporting Information.

**Figure 12:**
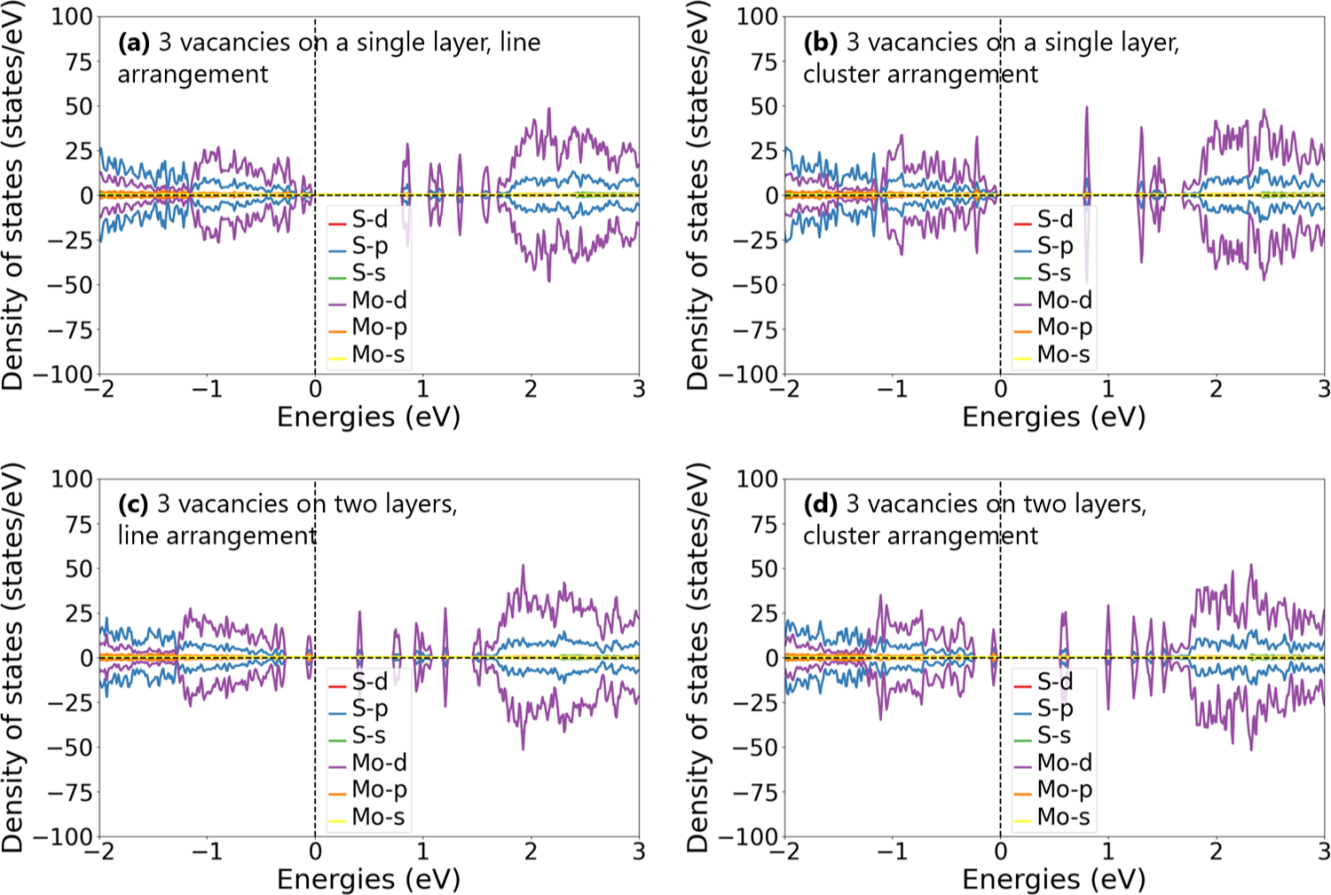
Orbital-projected DOS plots for MoS_2_ monolayers with three sulfur vacancies: (a) line arrangement on a single layer, (b) line arrangement on two layers, (c) cluster arrangement on a single layer, and (d) cluster arrangement on two layers.

We examined the average vacancy formation energy for line and cluster defects (Figure 13). A decrease in average vacancy formation energy was observed with increasing line defect length when the vacancies were located on a single atomic layer, corroborating previous findings and experimental evidence of thermally activated sulfur vacancies diffusion to create ultralong line defects.^79–83^ In contrast, the required energy to construct a line defect of vacancies located on both layers intermittently is substantially greater than that needed for a line defect of an equivalent length of vacancies located on the same layer. The lower vacancy formation energies for line defects on one layer are consistent with the longer chemical bonds associated with the lower electronic conductivity seen for this defect configuration. The average formation energy of cluster defects rises with an increase in the number of vacancies; namely, this configuration formation is less energetically favorable, suggesting that cluster vacancy arrangements are less likely to form. For the average vacancy formation energies of all calculated structures, see Supporting Information Table S2 and Figure S4.

**Figure 13:**
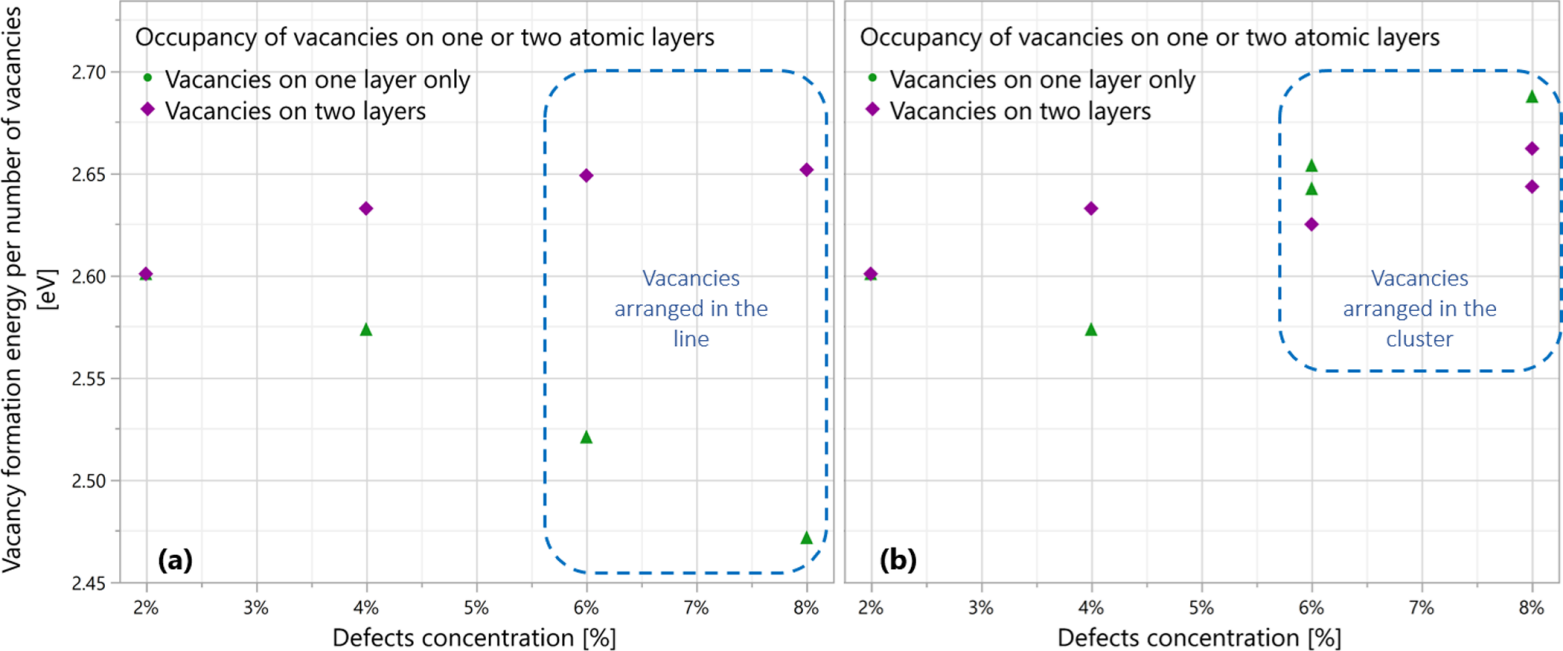
Average vacancy formation energies versus vacancy concentration. (a) Presents a line defect formation and (b) cluster defect formation. The vacancy formation energies were calculated using eq 5, while the chemical potential parameter was calculated to be −4.1146 eV.

## Conclusion

In summary, we investigated the effect of sulfur vacancy distribution on charge transport across MoS_2_ monolayers. The methods we used in this work are (1) spin-polarized DFT, a widely used approach for characterizing material properties, which provide the electronic structure data; (2) the TB theory to construct the TB Hamiltonian; (3) NEGF formalism for transmission function calculations, (4) Landauer–Büttiker formalism for charge transport calculations, and (5) DOE method for the analysis of the charge transport dependency on the applied bias, sulfur vacancy concentration, layer occupancy, and vacancy planar arrangement.

DFT calculations revealed that the electronic structure of defected MoS_2_ monolayers is significantly influenced by the presence of sulfur vacancies. The presence of sulfur vacancies generated midgap states in the band gap, with different intensities and energies, depending on the vacancy’s concentrations and relative positions. In all structures, most states close to the valence and conduction bands as well as the majority of the newly generated states belong to the d orbitals of Mo atoms, although there is also not negligible presence of states that belong to the p orbitals of sulfur atoms. The calculated electronic structures were subsequently used for electric current, ionization potential, and band gap energy calculations.

Previous studies have shown that sulfur vacancies enhance the electric current. Our analysis confirms this finding and further reveals a significant positive correlation between the concentration of sulfur vacancies and the magnitude of the current. We observed a difference in current between line and cluster vacancy arrangements, with linear arrangements exhibiting higher values. The distribution of sulfur vacancies across both atomic layers notably enhances the current in both arrangements. This effect was most pronounced for closely spaced vacancies and diminished with increasing intervacancy distance due to the limited influence range of the vacancies.

Analysis of calculated ionization potential, band gap energies, and average Mo–S bond length across all structures revealed a clear correlation with the concentration of sulfur vacancies. As the concentration of sulfur vacancies increases, the values of these parameters decrease. This reduction intuitively contributes to the enhancement of the electric conductivity of MoS_2_ monolayers. Furthermore, we noticed that these three parameters were significantly affected by the layer occupancy. For the same vacancy concentration, and the same line or cluster arrangement, ionization potential, band gap energy, and average Mo–S bond length decrease when vacancies were located on both layers compared to a single layer. We also observed this trend for the distribution of Mo–S bond lengths, which was significantly lower when vacancies were located on both layers for both arrangements.

By comparing the average vacancy formation energies for different structures, we clearly see that there are the most and the least energetically favorable arrangements for vacancy formation. For example, there is a preference for line vacancy arrangements when the vacancies are located on the same sulfur atomic layer, where in this type of defect, the average vacancy formation energy decreased as the vacancy concentration increased, indicating that line-arranged vacancies are easier to form. Conversely, the cluster arrangements are found to be the least energetically favorable, suggesting a lower likelihood for spontaneous formation of these types of defects.

Using full factorial DOE analysis assisted in revealing the main factors and interactions influencing the current in the studied structures. As shown, a good agreement between the DOE fitted model’s predicted results and the calculated current values is obtained.

This study contributes to an understanding of the effect of sulfur vacancy distribution on charge transport across MoS_2_ monolayers. We found that the electron transport was significantly enhanced by increasing vacancy concentration, with the greatest effect observed when sulfur vacancies were arranged in line with the alternating presence across both layers. The ability to understand and predict how sulfur vacancy distribution in MoS_2_ monolayers will affect the electric properties through selective integration of structural defects is especially important for rationally designed new functional interfaces, which can be used in various applications, such as development of transistors, sensors, fuel cells, and more.

## Supplementary Material





## Notes

Basic data and schemes of the MoS_2_ models; summary of DFT calculation results: calculated energies (total, band gap, ionization potential, and vacancy formation), summary of DOS results, data summary of structure parameters and Mo–S bond length, and examples of charge density difference (CDD) images; charge transport calculations results: side-by-side BS, DOS, *T*(*E*), and *IV* plots for three different structures, summary of the current calculation as a function of bias, current vs as a function of bias plots; workflow for charge transport calculation: section that details the main Python scripts and workflow used to calculate electron charge transport; DOE and data analysis results: the full factorial 5 × 2 × 2 design matrix, predicted and calculated current values versus the applied bias, interaction profiles plot; and input data for DFT calculations: KPOINTS and optimized structures (POSCAR files) (PDF)
Interactive fit least squares report produced by JMP software (ZIP)

## Abbreviations

ANOVA: analysis of variance
BS: band structure
BZ: Brillouin zone
CBM: conduction band minimum
DFT: density functional theory
DOE: design of experiments
DOS: density of states
E_g_: band gap energy
FF: full factorial
GGA: generalized gradient approximation
IP: ionization potential
NEGF: nonequilibrium Green’s function
PAW: projector-augmented wave
PBE: Perdew–Burke–Ernzerhof functional
TMDs: transition metal dichalcogenides
VASP: Vienna ab initio simulation package
VBM: valence band maximum

## References

[1] Wang Q. H., Kalantar-Zadeh K., Kis A., Coleman J. N., Strano M. S., Kis A., Coleman J. N., Coleman J. N., Strano M. S., Strano M. S. (2012). Electronics
and Optoelectronics of Two-Dimensional Transition Metal Dichalcogenides. Nat. Nanotechnol..

[2] Yin Z., Li H., Li H. O., Li H., Jiang L., Li H., Shi Y., Li H., Sun Y., Zhang H., Jiang L., Shi Y., Zhang Q., Lu G., Zhang Q., Chen X., Zhang H. (2012). Single-Layer MoS2 Phototransistors. ACS Nano.

[3] Ganatra R., Zhang Q. (2014). Few-Layer MoS2: A Promising
Layered Semiconductor. ACS Nano.

[4] Yoon Y., Ganapathi K., Salahuddin S. (2011). How Good Can Monolayer MoS2 Transistors
Be?. Nano Lett..

[5] Lopez-Sanchez O., Lembke D., Kayci M., Radenovic A., Kis A. (2013). Ultrasensitive Photodetectors Based on Monolayer MoS 2. Nat. Nanotechnol..

[6] Li H., Yin Z., He Q., Li H., Huang X., Lu G., Fam D. W. H., Tok A. I. Y., Zhang Q., Zhang H. (2012). Fabrication
of Single- and Multilayer MoS 2 Film-Based Field-Effect Transistors
for Sensing NO at Room Temperature. Small.

[7] Chen X., Park Y. J., Kang M., Chen X., Koo J., Kang S. K., Shin J., Jeon S., Park G., Yan Y., MacEwan M. R., Ray W. Z., Lee K. M., Rogers J. A., Ahn J. H., Shin J., Jeon S. H., Sung-eok J., Jeon S., Gayoung P., Park G., Yan Y., Yan Y., Matthew R. M.E., MacEwan M. R., Wilson Z. R., Ray W. Z., Lee H. K., Kyung Mi L., Lee K. M., Rogers J. A., Rogers J. A., Jong H. A., Ahn J. H. (2018). CVD-Grown Monolayer
MoS2 in Bioabsorbable Electronics and Biosensors. Nat. Commun..

[8] Mak K. F., Lee C., Hone J., Shan J., Heinz T. F. (2010). Atomically Thin
MoS2: A New Direct-Gap Semiconductor. Phys.
Rev. Lett..

[9] Yoon J., Park W., Bae G., Kim Y., Jang H. S., Hyun Y., Lim S. K., Kahng Y. H., Hong W., Lee B. H., Ko H. C., Yujun H., Hyun Y., Sung K. L., Lim S. K., Yung Ho K., Kahng Y. H., Woong Ki H., Hong W.-K., Byoung H. L., Lee B. H., Heung C. K., Ko H. C. (2013). Highly Flexible
and Transparent Multilayer
MoS2 Transistors with Graphene Electrodes. Small.

[10] Kim T. Y., Ha J., Cho K., Pak J., Seo J., Park J., Kim J. K., Chung S., Hong Y., Lee T., Seo J., Jongjang P., Park J., Kim J.-K., Kim J.-K., Chung S., Chung S., Yongtaek H., Hong Y., Takhee L., Lee T. (2017). Transparent Large-Area MoS2 Phototransistors
with Inkjet-Printed Components on Flexible Platforms. ACS Nano.

[11] Kim S., Konar A., Kim S., Hwang W. S., Lee J., Lee J. H., Jung C., Kim H., Yoo J. B., Choi J. Y., Jin Y. W., Lee S. Y., Jena D., Choi W., Kim K., Jung C. W., Jung C., Kim H., Kim H., Yoo Ji-B., Yoo J.-B., Jae-Young C., Choi J.-Y., Jin Y., Jin Y. W., Sang Y. L., Lee S. Y., Sang Y. L., Lee S. Y., Debdeep J., Jena D., Woong C., Choi W., Kim K., Kim K. (2012). High-Mobility and Low-Power Thin-Film Transistors Based on Multilayer
MoS2 Crystals. Nat. Commun..

[12] Xiao J., Chen K., Zhang X., Liu X.-z., Yu H., Gao L., Hong M., Gu L., Zhang Z., Zhang Y. (2023). Approaching
Ohmic Contacts for Ideal Monolayer MoS_2_ Transistors Through
Sulfur-Vacancy Engineering. Small Methods.

[13] Yu Z., Ong Z., Pan Y., Cui Y., Xin R., Shi Y., Wang B., Wu Y., Chen T., Zhang Y., Zhang G., Wang X., Wang B., Wang B., Wu Y., Wu Y., Wu Y., Wu Y., Che T., Che T., Zhang Y.-W., Zhang Y.-W., Zhang G., Zhang G., Wang X., Wang X., Wang X. (2015). Realization of Room-Temperature
Phonon-Limited Carrier Transport in Monolayer MoS2 by Dielectric and
Carrier Screening. Adv. Mater. Sci..

[14] Radisavljevic B., Radenovic A., Brivio J., Giacometti V., Kis A. (2011). Single-Layer MoS2 Transistors. Nat. Nanotechnol..

[15] Novoselov K. S., Jiang D., Schedin F., Booth T. J., Khotkevich V. V., Morozov S. V., Geim A. K. (2005). Two-Dimensional
Atomic Crystals. Proc. Natl. Acad. Sci. U.S.A..

[16] Wang S., Robertson A., Warner J. H. (2018). Atomic Structure of Defects and Dopants
in 2D Layered Transition Metal Dichalcogenides. Chem. Soc. Rev..

[17] Liu H., Han N., Zhao J. (2015). Atomistic Insight into the Oxidation of Monolayer Transition
Metal Dichalcogenides: From Structures to Electronic Properties. RSC Adv..

[18] Mitterreiter E., Schuler B., Micevic A., Hernangómez-Pérez D., Barthelmi K., Cochrane K. A., Kiemle J., Sigger F., Klein J., Wong E., Barnard E. S., Watanabe K., Taniguchi T., Lorke M., Jahnke F., Finley J. J., Schwartzberg A. M., Qiu D. Y., Refaely-Abramson S., Holleitner A. W., Weber-Bargioni A., Kastl C. (2021). The Role of Chalcogen
Vacancies for Atomic Defect Emission in MoS2. Nat. Commun..

[19] Rai D. P., Vu T. V., Laref A., Ghimire M. p., Patra P. k., Srivastava S., Laref A., Laref A., Madhav P. G., Ghimire M. P., Ghimire M. P., Patra P. K., Patra P. K., Sunita S., Srivastava S. (2020). Electronic and Optical Properties
of 2D Monolayer (ML) MoS2 with Vacancy Defect at S Sites. Nano-Struct. Nano-Objects.

[20] Qiu H., Xu T., Wang Z., Ren W., Nan H., Ni Z., Chen Q., Yuan S., Miao F., Song F., Long G., Shi Y., Sun L., Wang J., Wang X., Yuan S., Feng M., Miao F., Song F., Song F., Long G., Long G., Gen L., Yi S., Shi Y., Litao S., Sun L., Wang J., Wang J., Wang X., Wang X., Wang X. (2013). Hopping Transport through
Defect-Induced Localized States in Molybdenum
Disulphide. Nat. Commun..

[21] Lee J., Kim M. J., Jeong B. G., Kwon C., Cha Y., Choi S. H., Kim K. K., Jeong M. S. (2023). Electrical Role
of Sulfur Vacancies in MoS2: Transient Current Approach. Appl. Surf. Sci..

[22] Kodama N., Hasegawa T., Okawa Y., Tsuruoka T., Joachim C., Aono M. (2010). Electronic States of
Sulfur Vacancies Formed on a MoS _2_ Surface. Jpn. J. Appl. Phys..

[23] Gu M., Han M., Kim S. (2024). Electron Transport through the Multiple Sulfur Vacancies
in MoS2. Curr. Appl. Phys..

[24] Chee S.-S., Oh C., Son M., Son G.-C., Jang H., Yoo T. J., Lee S., Lee W., Hwang J. Y., Choi H., Lee B. H., Ham M.-H. (2017). Sulfur
Vacancy-Induced Reversible Doping of Transition
Metal Disulfides via Hydrazine Treatment. Nanoscale.

[25] Chaves A., Azadani J. G., Alsalman H., da Costa D. R., Frisenda R., Chaves A. J., da Costa D. R., da Costa D. R., He D., Frisenda R., Zhou J., Frisenda R., Castellanos-Gomez A., Hinkle C. L., Peeters F. M., Ye P. D., Liu Z., Lee Y. H., Avouris P., Wang X., Low T., Jiadong Z., Song S. H., Jiadong Z., Kim Y. D., Castellanos-Gomez A., Castellanos-Gomez A., Castellanos-Gomez A., Peeters F. M., Peeters F. M., Zheng L., Liu Z., Liu Z., Zheng L., Zheng L., Zheng L., Hinkle C. L., Hinkle C. L., Sang H. O., Oh S. H., Oh S.-H., Oh S.-H., Ye P. D., Ye P. D., Steven J. K., Koester S. J., Young H. L., Lee Y. H., Avouris Ph., Avouris P., Wang X., Wang X., Wang X., Tony L., Low T. (2020). Bandgap Engineering of Two-Dimensional
Semiconductor Materials. npj 2D Mater. Appl..

[26] Addou R., McDonnell S., Barrera D., Guo Z., Azcatl A., Wang J., Zhu H., Hinkle C. L., Quevedo-Lopez M., Alshareef H. N., Wang J., Hsu J. W. P., Colombo L., Wang J., Hai-Liang Z., Zhu H., Wallace R. M., Hinkle C. L., Manuel Q.-L., Quevedo-Lopez M., Husam N. A., Alshareef H. N., Luigi C., Colombo L., Julia W. P. H., Hsu J. W. P., Wallace R. M., Wallace R. M. (2015). Impurities
and Electronic Property Variations of Natural MoS2 Crystal Surfaces. ACS Nano.

[27] Yang J., Bussolotti F., Kawai H., Goh K. E. J. (2020). Tuning the Conductivity
Type in Monolayer Ws2 and Mos2 by Sulfur Vacancies. Phys. Status Solidi RRL.

[28] Ghorbani-Asl M., Enyashin A. N., Kuc A., Seifert G., Heine T. (2013). Defect-Induced
Conductivity Anisotropy in MoS 2 Monolayers. Phys. Rev. B:Condens. Matter Mater. Phys..

[29] Ben-Melech
Stan G., Caspary Toroker M. (2019). On the Nature of Trapped States in
an MoS2 Two-Dimensional Semiconductor with Sulfur Vacancies. Mol. Phys..

[30] Momma K., Izumi F. (2011). VESTA 3 for Three-Dimensional Visualization
of Crystal, Volumetric
and Morphology Data. J. Appl. Crystallogr..

[31] Palmer, D. C. ; Fernandez, A. ; Gao, M. ; Palmer, E. CrystalMaker: A Crystal and Molecular Structures Program for Mac and Windows; CrystalMaker Software Ltd Oxford, 2020.

[32] Tsai C., Li H., Park S., Park J., Han H. S., Nørskov J. K., Zheng X., Abild-Pedersen F. (2017). Electrochemical Generation of Sulfur
Vacancies in the Basal Plane of MoS2 for Hydrogen Evolution. Nat. Commun..

[33] Li H., Tsai C., Koh A. L., Cai L., Contryman A. W., Fragapane A. H., Zhao J., Han H. S., Manoharan H. C., Abild-Pedersen F., Nørskov J. K., Zheng X. (2016). Erratum: Activating
and Optimizing MoS2 Basal Planes for Hydrogen Evolution through the
Formation of Strained Sulphur Vacancies (Nature Materials (2016) 15
(48–53)). Nat. Mater..

[34] Zhu Y., Lim J., Zhang Z., Wang Y., Sarkar S., Ramsden H., Li Y., Yan H., Phuyal D., Gauriot N., Rao A., Hoye R. L. Z., Eda G., Chhowalla M. (2023). Room-Temperature
Photoluminescence Mediated by Sulfur Vacancies in 2D Molybdenum Disulfide. ACS Nano.

[35] McDonnell S., Addou R., Buie C., Wallace R. M., Hinkle C. L. (2014). Defect-Dominated
Doping and Contact Resistance in MoS_2_. ACS Nano.

[36] Gali S. M., Pershin A., Lherbier A., Charlier J.-C., Beljonne D. (2020). Electronic
and Transport Properties in Defective MoS_2_: Impact of Sulfur
Vacancies. J. Phys. Chem. C.

[37] Kresse G., Furthmüller J. (1996). Efficient Iterative Schemes for Ab
Initio Total-Energy
Calculations Using a Plane-Wave Basis Set. Phys.
Rev. B:Condens. Matter Mater. Phys..

[38] Kresse G., Joubert D. (1999). From Ultrasoft Pseudopotentials
to the Projector Augmented-Wave
Method. Phys. Rev. B:Condens. Matter Mater.
Phys..

[39] Perdew J. P., Burke K., Ernzerhof M. (1996). Generalized Gradient Approximation
Made Simple. Phys. Rev. Lett..

[40] Stan G. B. M., Dhaka K., Toroker M. C. (2020). Charge
Transport Calculation along
Two-Dimensional Metal/Semiconductor/Metal Systems. Isr. J. Chem..

[41] Tsai, Y.-C. ; Chen, C.-Y. ; Ho, M.-S. ; Li, Y. Work Function Modulation of Monolayer MOS_2_ Doped with 3d Transition Metals. In 2017 75th Annual Device Research Conference (DRC); IEEE, 2017; pp 1–2.

[42] Komsa H.-P., Kotakoski J., Kurasch S., Lehtinen O., Kaiser U., Krasheninnikov A. V., Ossi L., Lehtinen O., Ute K., Kaiser U., Arkady V. K., Krasheninnikov A. V. (2012). Two-Dimensional
Transition Metal Dichalcogenides under Electron Irradiation: Defect
Production and Doping. Phys. Rev. Lett..

[43] Blöchl P. E., Blöchl P. E. (1994). Projector
Augmented-Wave Method. Phys. Rev. B:Condens.
Matter Mater. Phys..

[44] Hinuma Y., Pizzi G., Kumagai Y., Oba F., Tanaka I. (2017). Band Structure
Diagram Paths Based on Crystallography. Comput.
Mater. Sci..

[45] Togo, A. ; Shinohara, K. ; Tanaka, I. S. A Software Library for Crystal Symmetry Search. 2018, Arxiv 1808.01590

[46] Landauer R. (1957). Spatial Variation
of Currents and Fields Due to Localized Scatterers in Metallic Conduction. IBM J. Res. & Dev..

[47] Datta, S. Quantum Transport: Atom to Transistor; Cambridge University Press, 2005.

[48] Elbaz Y., Caspary Toroker M. (2024). From Density
Functional Theory to Machine Learning
Predictive Models for Electrical Properties of Spinel Oxides. Sci. Rep..

[49] Elbaz, Y. Modeling and Prediction of Electron Transfer in Spinel Oxides; Technion-Israel Institute of Technology, 2022.

[50] Kittel, C. Introduction to Solid State Physics; Wiley: Hoboken, N.J, 2005.

[51] Ibach, H. ; Lüth, H. Solid-State Physics an Introduction to Principles of Materials Science, Advanced Texts in Physics; Springer: Berlin Heidelberg, 2009; . 10.1007/978-3-540-93804-0.

[52] Simon, S. H. The Oxford Solid State Basics; University of Oxford.; Oxford University Press: Oxford, 2013.

[53] Zahid F., Liu L., Zhu Y., Wang J., Guo H. (2013). A Generic Tight-Binding
Model for Monolayer, Bilayer and Bulk MoS 2. AIP Adv..

[54] Goringe C. M., Bowler D. R., Hernández E. (1997). Tight-Binding
Modelling of Materials. Rep. Prog. Phys..

[55] Slater J. C., Koster G. F. (1954). Simplified LCAO
Method for the Periodic Potential Problem. Phys.
Rev..

[56] Lee D. H., Joannopoulos J. D. (1981). Simple
Scheme for Surface-Band Calculations. I. Phys.
Rev. B:Condens. Matter Mater. Phys..

[57] Lee D. H., Joannopoulos J. D. (1981). Simple Scheme for Surface-Band Calculations.
II. The
Green’s Function. Phys. Rev. B:Condens.
Matter Mater. Phys..

[58] Wang Z., Ye S., Wang H., He J., Huang Q., Chang S. (2021). Machine Learning
Method for Tight-Binding Hamiltonian Parameterization from Ab-Initio
Band Structure. npj Comput. Mater..

[59] Kingma, D. P. ; Ba, J. Adam: A Method for Stochastic Optimization. 2017, arXiv 1412.6980

[60] Sørensen H. H. B., Hansen P. C., Petersen D. E., Skelboe S., Stokbro K. (2008). Krylov Subspace
Method for Evaluating the Self-Energy Matrices in Electron Transport
Calculations. Phys. Rev. B:Condens. Matter Mater.
Phys..

[61] Smithe K. K. H., English C. D., Suryavanshi S. V., Pop E. (2017). Intrinsic Electrical
Transport and Performance Projections of Synthetic Monolayer MoS_2_ Devices. 2D Mater..

[62] Enyashin A., Seifert G. (2014). Electronic Properties of MoS2Monolayer
and Related
Structures. Nanosyst.:Phys., Chem., Math..

[63] Li W. (2015). Electrical
Transport Limited by Electron-Phonon Coupling from Boltzmann Transport
Equation: An Ab Initio Study of Si, Al, and MoS 2. Phys. Rev. B:Condens. Matter Mater. Phys..

[64] Frensley, W. R. Quantum Transport. In VLSI Electronics Microstructure Science; Elsevier, 1994; Vol. 24, pp 273–303.

[65] Ozaki T., Nishio K., Kino H. (2010). Efficient Implementation of the Nonequilibrium
Green Function Method for Electronic Transport Calculations. Phys. Rev. B:Condens. Matter Mater. Phys..

[66] Peskin U. (2010). An Introduction
to the Formulation of Steady-State Transport through Molecular Junctions. J. Phys. B: At., Mol. Opt. Phys..

[67] Velev J., Butler W. (2004). On the Equivalence
of Different Techniques for Evaluating
the Green Function for a Semi-Infinite System Using a Localized Basis. J. Phys.: Condens. Matter.

[68] Montgomery, D. C. Design and Analysis of Experiments, 8th ed.; John Wiley & Sons, Incorporated, 2012.

[69] SAS Institute Inc. JMP Pro, Version 18.0.2: Cary, NC, 1989–2025, http://www.jmp.com.

[70] Miralrio A., Rangel Cortes E., Castro M. (2018). Electronic Properties and Enhanced
Reactivity of MoS 2 Monolayers with Substitutional Gold Atoms Embedded
into Sulfur Vacancies. Appl. Surf. Sci..

[71] Freysoldt C., Grabowski B., Hickel T., Neugebauer J., Kresse G., Janotti A., Van De Walle C. G. (2014). First-Principles
Calculations for Point Defects in Solids. Rev.
Mod. Phys..

[72] Komsa H. P., Krasheninnikov A. V. (2015). Native
Defects in Bulk and Monolayer MoS2 from First
Principles. Phys. Rev. B:Condens. Matter Mater.
Phys..

[73] Noh J. Y., Kim H., Kim Y. S. (2014). Stability
and Electronic Structures of Native Defects
in Single-Layer MoS2. Phys. Rev. B:Condens.
Matter Mater. Phys..

[74] Park W., Park J., Jang J., Lee H., Jeong H., Cho K., Hong S., Lee T., Lee H. W., Hyunhak J., Jeong H., Kyungjune C., Cho K., Sok C. H., Hong S., Takhee L., Lee T. (2013). Oxygen Environmental
and Passivation Effects on Molybdenum Disulfide Field Effect Transistors. Nanotechnology.

[75] Feng L., Su J., Liu Z. (2014). Effect of Vacancies on Structural, Electronic and Optical
Properties of Monolayer MoS2: A First-Principles Study. J. Alloys Compd..

[76] Kahn A. (2016). Fermi Level,
Work Function and Vacuum Level. Mater. Horiz..

[77] Hu G., Fung V., Huang J., Ganesh P. (2021). Work Function Engineering
of 2D Materials: The Role of Polar Edge Reconstructions. J. Phys. Chem. Lett..

[78] Leung T. C., Kao C. L., Su W. S., Feng Y. J., Chan C. T. (2003). Relationship
between Surface Dipole, Work Function and Charge Transfer: Some Exceptions
to an Established Rule. Phys. Rev. B:Condens.
Matter Mater. Phys..

[79] Le D., Rawal T. B., Rahman T. S. (2014). Single-Layer
MoS2 with Sulfur Vacancies:
Structure and Catalytic Application. J. Phys.
Chem. C.

[80] Wang S., Lee S., Lee G. D., Yoon E., Warner J. H., Sung-Woo L., Lee S., Euijoon Y., Yoon E., Jamie H. W., Warner J. H. (2016). Detailed
Atomic Reconstruction of Extended Line Defects in Monolayer MoS2. ACS Nano.

[81] Chen Q., Li H., Zhou S., Xu W., Chen J., Sawada H., Allen C. S., Kirkland A. I., Chen J., Chen J., Warner J. H., Jun C., Chen J., Hidetaka S., Sawada H., Allen C. S., Allen C. S., Grossman J. C., Kirkland A. I., Jeffrey C. G., Grossman J. C., Jamie H. W., Warner J. H. (2018). Ultralong 1D Vacancy Channels for Rapid Atomic Migration
during 2D Void Formation in Monolayer MoS2. ACS Nano.

[82] Komsa H.-P., Kurasch S., Lehtinen O., Kaiser U., Krasheninnikov A. V., Lehtinen O., Ute K., Kaiser U., Krasheninnikov A. V., Krasheninnikov A. V. (2013). From Point to Extended Defects in Two-Dimensional MoS
2: Evolution of Atomic Structure under Electron Irradiation. Phys. Rev. B:Condens. Matter Mater. Phys..

[83] Liu M., Shi J., Li Y., Zhou X., Ma D., Qi Y., Zhang Y., Liu Z. (2017). Temperature-Triggered Sulfur Vacancy
Evolution in Monolayer MoS2/Graphene Heterostructures. Small.

