# Supplementary material for: The Effect of Sulfur Vacancy Distribution on Charge Transport across MoS2 Monolayers: A Quantum Mechanical Study
Source: ACS Mater Au. 2025 Jun 6;5(4):641–55. doi: 10.1021/acsmaterialsau.4c00171 (PMC12257400; doi:10.1021/acsmaterialsau.4c00171)
Supplement: Supplementary file 1 [file mg4c00171_si_001.pdf]

SUPPORTING INFORMATION

The Effect of Sulfur Vacancy Distribution on Charge Transport Across MoS_2 Monolayers: A Quantum Mechanical study

Hanna Kuperman Benedik [a], Naomi Rom [a], Maytal Caspary Toroker [a,b,c] ✉

a. Department of Materials Science and Engineering, Technion-Israel Institute of Technology, Haifa 3200003, Israel
b. The Nancy and Stephen Grand Technion Energy Program, Haifa 3200003, Israel
c. The Resnick Sustainability Center for Catalysis, Technion - Israel Institute of Technology, Haifa 3200003, Israel.

✉ Corresponding author: maytalc@technion.ac.il

Table of contents:

Basic data of the MoS_2 models 3

KPOINTS 6

Summary of DFT Calculation Results 7

Charge transport calculations 15

Workflow for charge transport calculation 18

Design of Experiments (DOE) and data analysis 19

Optimized structures (POSCAR files) 22

References 92

Basic data of the MoS_2 models

Table S1 – Details of the compositions of 5x5x1 MoS_2 superstructures provides information about the number of atoms in each structure that were used for calculations, including number of Mo and S atoms, and number of missing S atoms. The nomenclature of the structures is derived from the quantity of sulfur vacancies and their placement (top or bottom layer), and the specific location on the layer. For instance, '2vac_top3x3_bottom_1x3' indicates a structure with two vacancies: one on the top layer at position 3x3 and another on the bottom at position 1x3.

#	Sample NumberOfVacancies_LayerColxRow	Number of atoms					Vacancies concentration [%]
		Total	Mo	S total	S top layer	S bottom layer	
**1**	0vac	75	25	50	25	25	0%
**2**	1vac_bottom1x1	74	25	49	25	24	2%
**3**	1vac_top3x3	74	25	49	24	25	2%
**4**	2vac_top3x3_bottom1x2	73	25	48	24	24	4%
**5**	2vac_top3x3_bottom1x3	73	25	48	24	24	4%
**6**	2vac_top3x3_bottom2x2	73	25	48	24	24	4%
**7**	2vac_top3x3_bottom2x3	73	25	48	24	24	4%
**8**	2vac_top3x3_bottom3x2	73	25	48	24	24	4%
**9**	2vac_top3x3_bottom3x3	73	25	48	24	24	4%
**10**	2vac_top3x3_top1x2	73	25	48	23	25	4%
**11**	2vac_top3x3_top1x3	73	25	48	23	25	4%
**12**	2vac_top3x3_top2x2	73	25	48	23	25	4%
**13**	2vac_top3x3_top2x3	73	25	48	23	25	4%
**14**	2vac_top3x3_top5x4	73	25	48	23	25	4%
**15**	3vac_bottom3x3_top3x1_bottom5x1	72	25	47	23	24	6%
**16**	3vac_top1x3_bottom2x3_top3x3	72	25	47	23	24	6%
**17**	3vac_top1x3_top3x3_bottom3x1	72	25	47	23	24	6%
**18**	3vac_top1x3_top3x3_top3x1	72	25	47	22	25	6%
**19**	3vac_top3x3_bottom2x3_bottom4x2	72	25	47	24	23	6%
**20**	3vac_top3x3_bottom2x3_top3x2	72	25	47	23	24	6%
**21**	3vac_top3x3_bottom2x3_top3x5	72	25	47	23	24	6%
**22**	3vac_top3x3_bottom2x3_top4x2	72	25	47	23	24	6%
**23**	3vac_top3x3_bottom2x3_top4x4	72	25	47	23	24	6%
**24**	3vac_top3x3_top1x2_top2x4	72	25	47	22	25	6%
**25**	3vac_top3x3_top1x2_top3x5	72	25	47	22	25	6%
**26**	3vac_top3x3_top2x2_top1x1	72	25	47	22	25	6%
**27**	3vac_top3x3_top2x2_top2x4	72	25	47	22	25	6%
**28**	3vac_top3x3_top2x2_top3x5	72	25	47	22	25	6%
**29**	3vac_top3x3_top2x3_top1x3	72	25	47	22	25	6%
**30**	3vac_top3x3_top2x3_top3x2	72	25	47	22	25	6%
**31**	3vac_top3x3_top2x3_top4x2	72	25	47	22	25	6%
**32**	3vac_top3x3_top2x4_top4x2	72	25	47	22	25	6%
**33**	3vac_top3x3_top2x5_top4x2	72	25	47	22	25	6%

#	Sample<br>NumberOfVacancies_LayerColxRow	Number of atoms					Vacancies concentration [%]
		Total	Mo	S total	S top layer	S bottom layer	
**34**	3vac_top3x3_top2x5_top5x2	72	25	47	22	25	6%
**35**	3vac_top3x3_top3x1_top5x1	72	25	47	22	25	6%
**36**	3vac_top3x3_top3x2_top3x1	72	25	47	22	25	6%
**37**	3vac_top3x3_top3x2_top4x2	72	25	47	22	25	6%
**38**	3vac_top3x3_top4x2_top5x1	72	25	47	22	25	6%
**39**	3vac_top3x3_top4x4_top5x2	72	25	47	22	25	6%
**40**	4vac_top1x3_bottom2x3_top3x3_bottom4x3	71	25	46	23	23	8%
**41**	4vac_top1x3_top2x3_top3x3_top4x3	71	25	46	21	25	8%
**42**	4vac_top2x3_top3x3_top3x2_top4x2	71	25	46	21	25	8%
**43**	4vac_top2x4_top3x3_top4x2_top5x1	71	25	46	21	25	8%
**44**	4vac_top3x3_bottom2x3_top3x2_bottom4x2	71	25	46	23	23	8%
**45**	4vac_top3x3_bottom2x3_top3x2_top4x2	71	25	46	22	24	8%

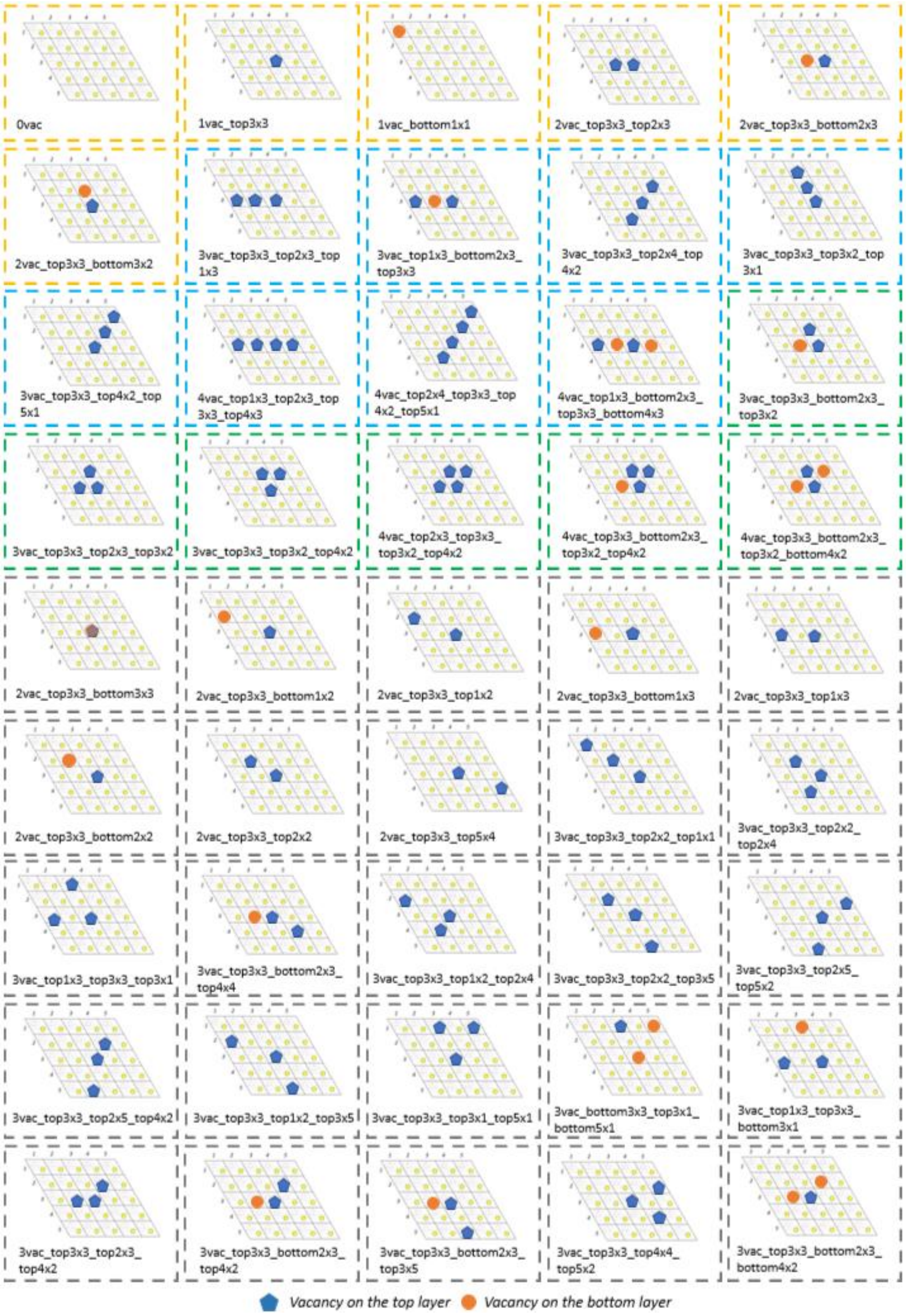

Figure S1 - The schemes of all MoS_2 structures that were used for the calculations. All models are 5x5 monolayers. Blue pentagons and orange circles represent vacancies on the top and bottom layers, respectively. Models within light blue frames are categorized as structures with line defects, those within green frames as structures with cluster defects, and those within gray frames as structures with "other" defects, neither a line nor a cluster. The single and adjacent double vacancies in the models within the orange frames may be classified as lines, clusters and "others".

KPOINTS

KPOINTS file contains the k-points of MoS_2 that were used for band structure calculations. KPOINTS file and the image bellow produced by SeeK-path[1,2].

<u>KPOINTS</u>:

```
k points along high symmetry lines
40
Line_mode
Reciprocal
  0.0000000000   0.0000000000   0.0000000000 !   \Gamma
  0.5000000000   0.0000000000   0.0000000000 !   M

  0.5000000000   0.0000000000   0.0000000000 !   M
  0.3333333333   0.3333333333   0.0000000000 !   K

  0.3333333333   0.3333333333   0.0000000000 !   K
  0.0000000000   0.0000000000   0.0000000000 !   \Gamma
```

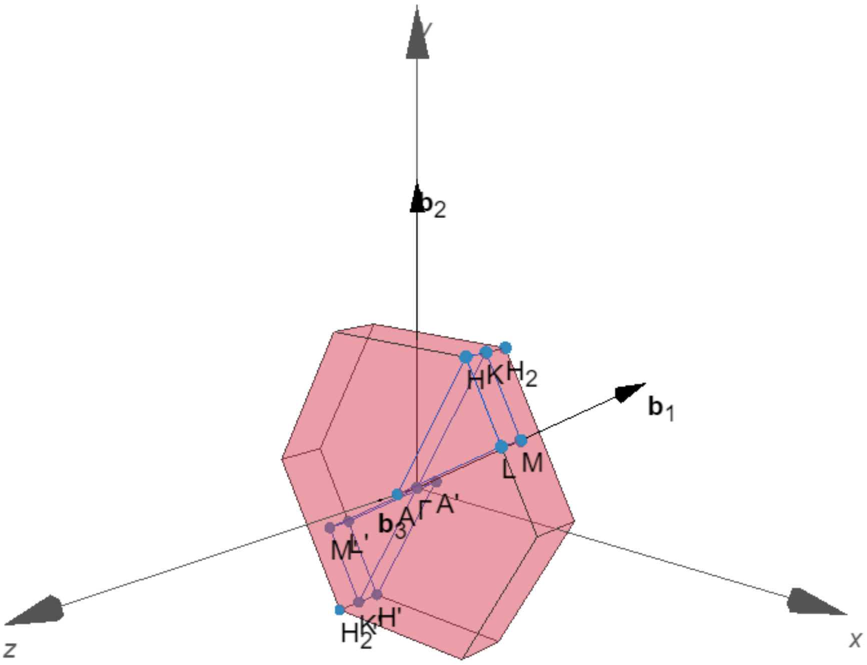

Figure S2 - The primitive Brillouin zone of MoS_2.

Summary of DFT Calculation Results

Table S2 - Summary of DFT-calculated energies (total, band gap, last occupied energy level, ionization potential, and vacancy formation) for various MoS_2 monolayers. Vacancies formation energies and ionization potential values were determined using equations (5) and (6), respectively, as described in the Methodology section. The μ_S parameter employed in equation (5) was calculated to be $-4.1146\ eV$.

#	**Sample** NumberOfVacancies_LayerColxRow	**Total energy per number of atoms [eV]**	**Band gap energy, Eg [eV]**	**Last occupied energy level [eV]**	**Vacuum energy [eV]**	**Ionization potential, IP [eV]**	**Vacancies formation energy [eV]**	**Vacancy formation energy per number of vacancies [eV]**
**1**	0vac	-7.234	1.586	-1.131	4.618	5.749		
**2**	1vac_bottom1x1	-7.241	1.082	-1.095	4.583	5.678	2.601	2.601
**3**	1vac_top3x3	-7.241	1.082	-1.095	4.587	5.682	2.601	2.601
**4**	2vac_top3x3_bottom1x2	-7.249	1.046	-1.095	4.560	5.655	5.154	2.577
**5**	2vac_top3x3_bottom1x3	-7.249	0.982	-1.095	4.559	5.654	5.167	2.583
**6**	2vac_top3x3_bottom2x2	-7.249	1.037	-1.104	4.555	5.659	5.160	2.580
**7**	2vac_top3x3_bottom2x3	-7.247	0.595	-1.023	4.549	5.571	5.266	2.633
**8**	2vac_top3x3_bottom3x2	-7.247	0.595	-1.014	4.549	5.562	5.266	2.633
**9**	2vac_top3x3_bottom3x3	-7.249	1.073	-1.122	4.557	5.679	5.143	2.572
**10**	2vac_top3x3_top1x2	-7.248	1.028	-1.095	4.557	5.652	5.178	2.589
**11**	2vac_top3x3_top1x3	-7.248	0.883	-1.041	4.565	5.606	5.208	2.604
**12**	2vac_top3x3_top2x2	-7.248	0.928	-1.068	4.564	5.632	5.184	2.592
**13**	2vac_top3x3_top2x3	-7.249	0.901	-1.068	4.574	5.641	5.147	2.574
**14**	2vac_top3x3_top5x4	-7.248	1.028	-1.095	4.564	5.659	5.178	2.589
**15**	3vac_bottom3x3_top3x1_bottom5x1	-7.256	0.874	-1.059	4.527	5.586	7.735	2.578
**16**	3vac_top1x3_bottom2x3_top3x3	-7.254	0.388	-0.951	4.516	5.467	7.947	2.649
**17**	3vac_top1x3_top3x3_bottom3x1	-7.256	0.883	-1.077	4.525	5.601	7.740	2.580
**18**	3vac_top1x3_top3x3_top3x1	-7.255	0.739	-0.951	4.522	5.473	7.835	2.612
**19**	3vac_top3x3_bottom2x3_bottom4x2	-7.254	0.451	-0.969	4.525	5.494	7.904	2.635
**20**	3vac_top3x3_bottom2x3_top3x2	-7.255	0.559	-0.987	4.523	5.510	7.875	2.625
**21**	3vac_top3x3_bottom2x3_top3x5	-7.255	0.559	-1.023	4.530	5.553	7.830	2.610
**22**	3vac_top3x3_bottom2x3_top4x2	-7.257	0.559	-1.014	4.533	5.547	7.718	2.573
**23**	3vac_top3x3_bottom2x3_top4x4	-7.255	0.595	-1.059	4.521	5.580	7.808	2.603
**24**	3vac_top3x3_top1x2_top2x4	-7.257	0.883	-1.086	4.521	5.607	7.725	2.575
**25**	3vac_top3x3_top1x2_top3x5	-7.256	0.820	-1.014	4.533	5.546	7.802	2.601
**26**	3vac_top3x3_top2x2_top1x1	-7.256	0.874	-1.068	4.524	5.592	7.744	2.581
**27**	3vac_top3x3_top2x2_top2x4	-7.256	0.739	-1.014	4.520	5.534	7.782	2.594
**28**	3vac_top3x3_top2x2_top3x5	-7.256	0.820	-1.023	4.521	5.543	7.771	2.590
**29**	3vac_top3x3_top2x3_top1x3	-7.259	0.793	-1.041	4.527	5.568	7.563	2.521
**30**	3vac_top3x3_top2x3_top3x2	-7.253	0.784	-1.086	4.527	5.613	7.961	2.654
**31**	3vac_top3x3_top2x3_top4x2	-7.257	0.811	-1.059	4.523	5.582	7.672	2.557
**32**	3vac_top3x3_top2x4_top4x2	-7.259	0.802	-1.041	4.526	5.567	7.563	2.521
**33**	3vac_top3x3_top2x5_top4x2	-7.257	0.838	-1.068	4.522	5.590	7.688	2.563

#	Sample NumberOfVacancies_LayerColxRow	Total energy per number of atoms [eV]	Band gap energy, Eg [eV]	Last occupied energy level [eV]	Vacuum energy [eV]	Ionization potential, IP [eV]	Vacancies formation energy [eV]	Vacancy formation energy per number of vacancies [eV]
**34**	3vac_top3x3_top2x5_top5x2	-7.256	0.784	-1.023	4.534	5.556	7.781	2.594
**35**	3vac_top3x3_top3x1_top5x1	-7.255	0.685	-0.987	4.521	5.508	7.864	2.621
**36**	3vac_top3x3_top3x2_top3x1	-7.259	0.793	-1.041	4.528	5.568	7.563	2.521
**37**	3vac_top3x3_top3x2_top4x2	-7.254	0.793	-1.068	4.526	5.594	7.927	2.642
**38**	3vac_top3x3_top4x2_top5x1	-7.259	0.802	-1.041	4.527	5.568	7.563	2.521
**39**	3vac_top3x3_top4x4_top5x2	-7.256	0.883	-1.041	4.526	5.566	7.747	2.582
**40**	4vac_top1x3_bottom2x3_top3x3_bottom4x3	-7.260	0.307	-0.933	4.475	5.408	10.607	2.652
**41**	4vac_top1x3_top2x3_top3x3_top4x3	-7.270	0.676	-1.014	4.501	5.515	9.887	2.472
**42**	4vac_top2x3_top3x3_top3x2_top4x2	-7.258	0.685	-1.077	4.494	5.571	10.750	2.688
**43**	4vac_top2x4_top3x3_top4x2_top5x1	-7.270	0.676	-1.014	4.500	5.513	9.887	2.472
**44**	4vac_top3x3_bottom2x3_top3x2_bottom4x2	-7.260	0.027	-0.842	4.471	5.313	10.648	2.662
**45**	4vac_top3x3_bottom2x3_top3x2_top4x2	-7.261	0.469	-0.942	4.490	5.431	10.574	2.643

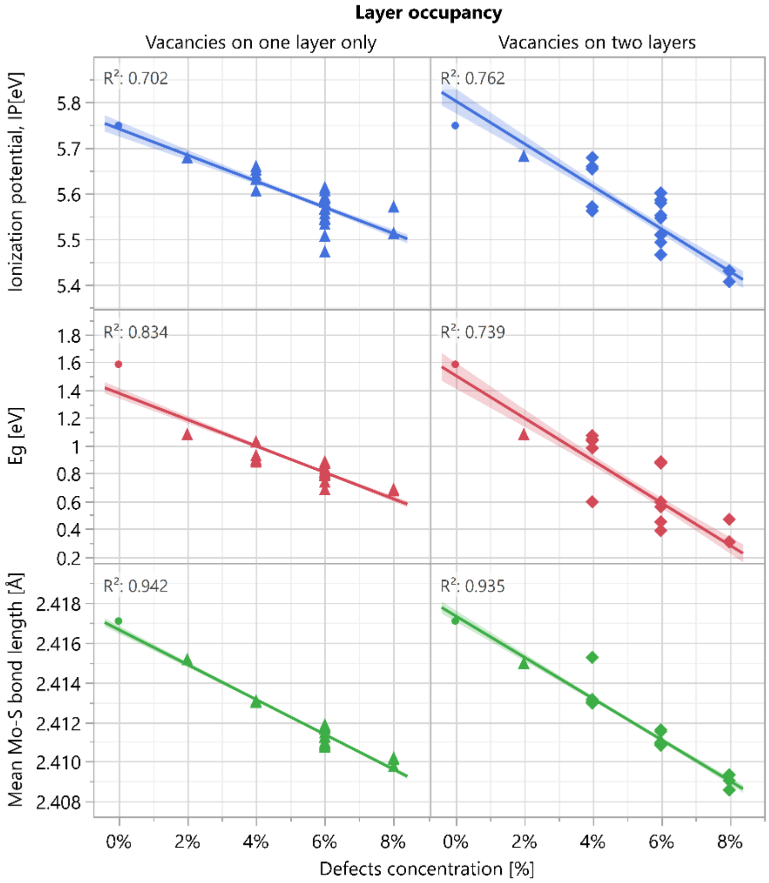

Figure S3 - Band gap energies (Eg), ionization potential (IP), and mean Mo-S bond length vs. vacancy concentration. The left plots belong to models with vacancies located on one sulfur layer only, and the right on both layers. Categorizing the results by layer occupancy revealed more clearly the inverse correlation between the parameters and vacancies concentration.

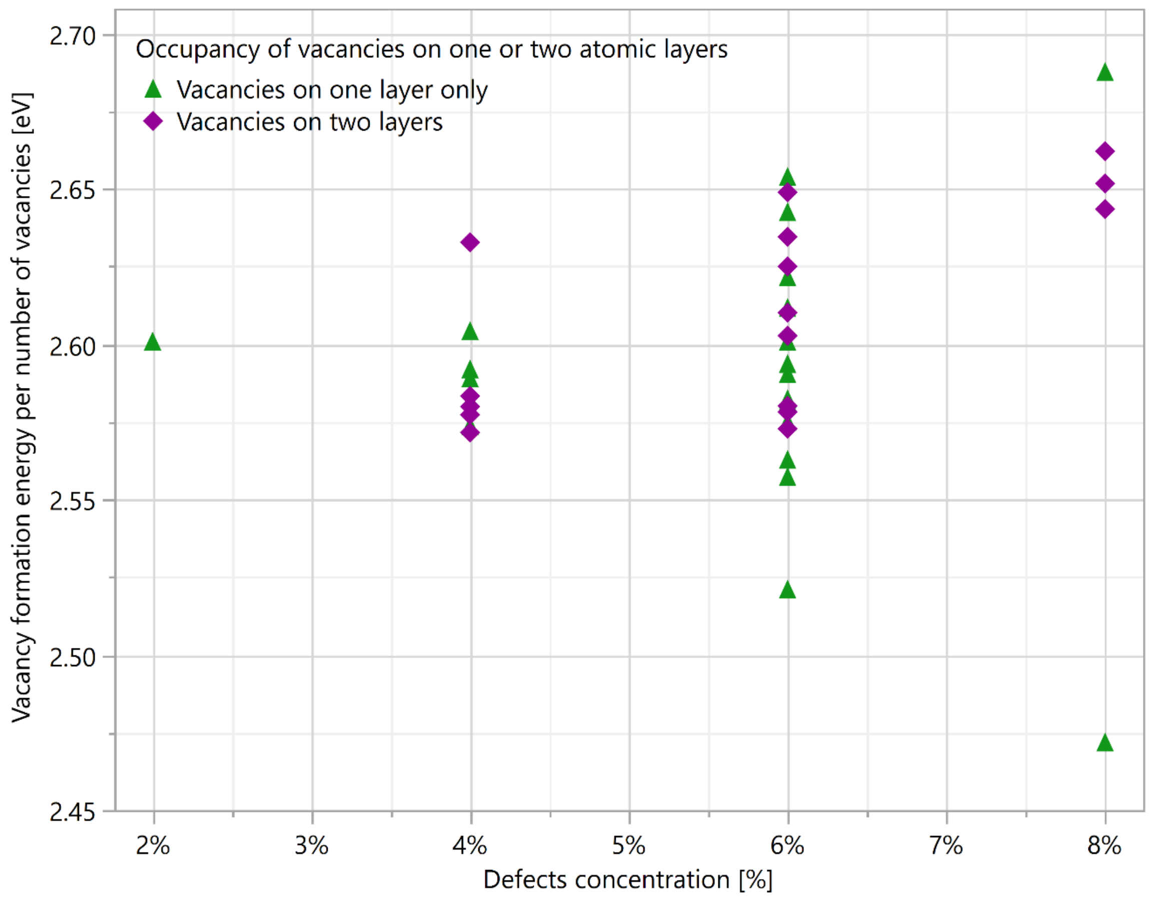

Figure S4 - Average vacancies formation energies versus vacancies concentration. The vacancy formation energies were calculated using equation (5), while the chemical potential parameter was calculated to be -4.1146 eV.

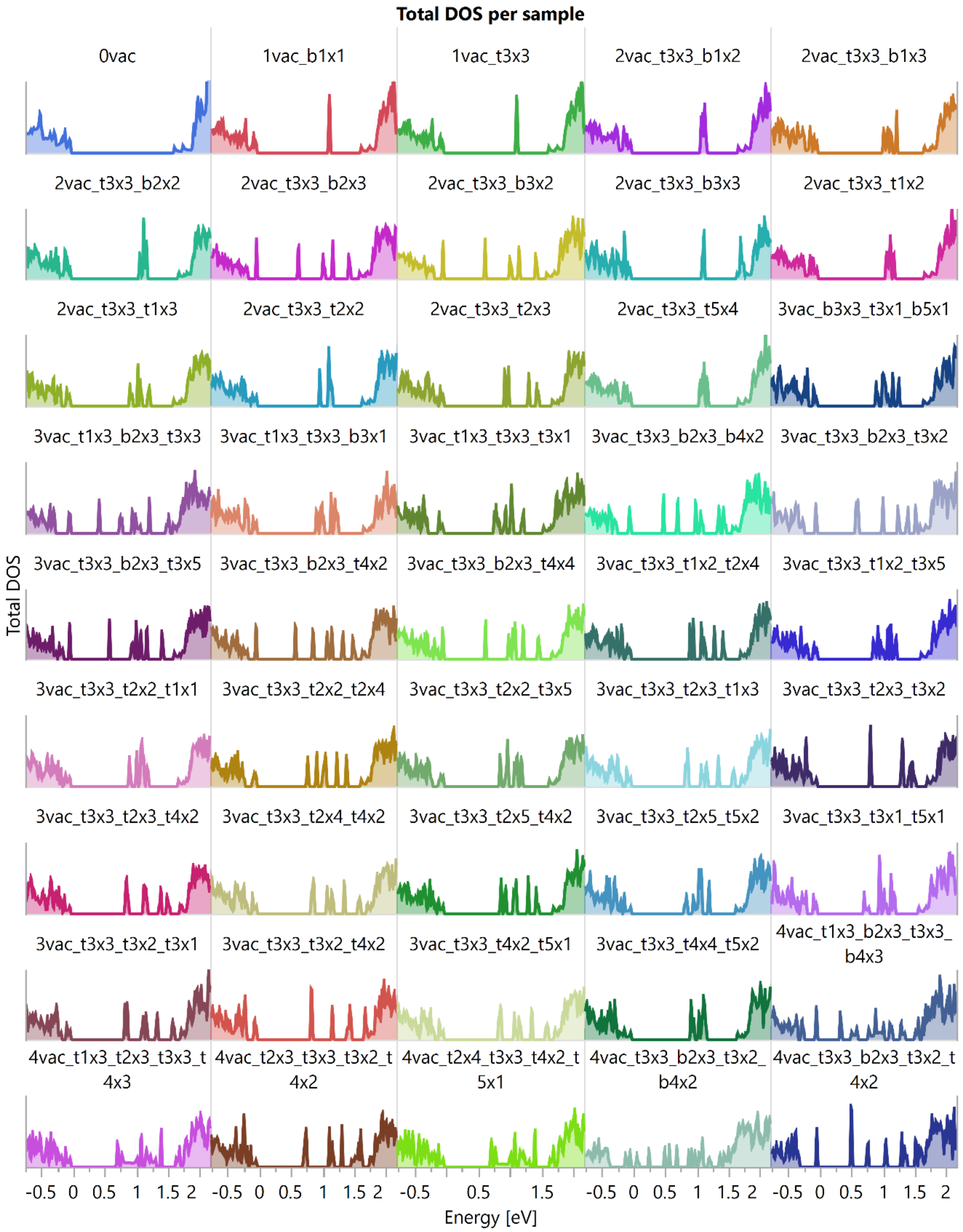

Figure S5 - Calculated total Density of States for different MoS_2 5x5x1 structures with various vacancies concentration and arrangements.

Table S3 - Data summary of Mo-S bond lengths in 5x5x1 MoS_2 structures, following structural relaxation.

#	Sample<br>NumberOfVacancies_LayerColxRow	Mo-S bonds length [Å]			
		Min	Max	Mean	Std Dev
**1**	0vac	2.4171	2.4171	2.4171	6.16E-06
**2**	1vac_bottom1x1	2.3824	2.4387	2.4152	0.0095
**3**	1vac_top3x3	2.3823	2.4384	2.4150	0.0095
**4**	2vac_top3x3_bottom1x2	2.3811	2.4428	2.4131	0.0134
**5**	2vac_top3x3_bottom1x3	2.3784	2.4392	2.4130	0.0127
**6**	2vac_top3x3_bottom2x2	2.3782	2.4423	2.4130	0.0131
**7**	2vac_top3x3_bottom2x3	2.3666	2.4552	2.4131	0.0145
**8**	2vac_top3x3_bottom3x2	2.3663	2.4553	2.4130	0.0145
**9**	2vac_top3x3_bottom3x3	2.3962	2.4545	2.4153	0.0145
**10**	2vac_top3x3_top1x2	2.3789	2.4496	2.4130	0.0136
**11**	2vac_top3x3_top1x3	2.3776	2.4405	2.4131	0.0132
**12**	2vac_top3x3_top2x2	2.3740	2.4477	2.4131	0.0130
**13**	2vac_top3x3_top2x3	2.3639	2.5175	2.4130	0.0170
**14**	2vac_top3x3_top5x4	2.3785	2.4493	2.4130	0.0136
**15**	3vac_bottom3x3_top3x1_bottom5x1	2.3808	2.4408	2.4109	0.0147
**16**	3vac_top1x3_bottom2x3_top3x3	2.3572	2.4538	2.4108	0.0182
**17**	3vac_top1x3_top3x3_bottom3x1	2.3777	2.4403	2.4109	0.0147
**18**	3vac_top1x3_top3x3_top3x1	2.3772	2.4392	2.4107	0.0155
**19**	3vac_top3x3_bottom2x3_bottom4x2	2.3566	2.4601	2.4110	0.0179
**20**	3vac_top3x3_bottom2x3_top3x2	2.3342	2.5209	2.4115	0.0195
**21**	3vac_top3x3_bottom2x3_top3x5	2.3663	2.4558	2.4109	0.0168
**22**	3vac_top3x3_bottom2x3_top4x2	2.3437	2.5451	2.4116	0.0211
**23**	3vac_top3x3_bottom2x3_top4x4	2.3638	2.4549	2.4110	0.0167
**24**	3vac_top3x3_top1x2_top2x4	2.3634	2.5237	2.4112	0.0195
**25**	3vac_top3x3_top1x2_top3x5	2.3760	2.4500	2.4109	0.0161
**26**	3vac_top3x3_top2x2_top1x1	2.3724	2.4576	2.4110	0.0157
**27**	3vac_top3x3_top2x2_top2x4	2.3626	2.4989	2.4113	0.0184
**28**	3vac_top3x3_top2x2_top3x5	2.3659	2.4477	2.4109	0.0153
**29**	3vac_top3x3_top2x3_top1x3	2.3633	2.5081	2.4118	0.0215
**30**	3vac_top3x3_top2x3_top3x2	2.3485	2.5145	2.4117	0.0224
**31**	3vac_top3x3_top2x3_top4x2	2.3491	2.5190	2.4119	0.0216
**32**	3vac_top3x3_top2x4_top4x2	2.3639	2.5080	2.4117	0.0215
**33**	3vac_top3x3_top2x5_top4x2	2.3631	2.5143	2.4113	0.0189
**34**	3vac_top3x3_top2x5_top5x2	2.3697	2.4467	2.4109	0.0153
**35**	3vac_top3x3_top3x1_top5x1	2.3793	2.4406	2.4108	0.0156
**36**	3vac_top3x3_top3x2_top3x1	2.3640	2.5084	2.4118	0.0215
**37**	3vac_top3x3_top3x2_top4x2	2.3619	2.4391	2.4115	0.0187
**38**	3vac_top3x3_top4x2_top5x1	2.3643	2.5078	2.4118	0.0215
**39**	3vac_top3x3_top4x4_top5x2	2.3749	2.4485	2.4109	0.0153
**40**	4vac_top1x3_bottom2x3_top3x3_bottom4x3	2.3514	2.4545	2.4086	0.0210

#	Sample<br>NumberOfVacancies_LayerColxRow	Mo-S bonds length [Å]			
		Min	Max	Mean	Std Dev
**41**	4vac_top1x3_top2x3_top3x3_top4x3	2.3594	2.5048	2.4102	0.0251
**42**	4vac_top2x3_top3x3_top3x2_top4x2	2.3463	2.5166	2.4098	0.0239
**43**	4vac_top2x4_top3x3_top4x2_top5x1	2.3592	2.5054	2.4101	0.0252
**44**	4vac_top3x3_bottom2x3_top3x2_bottom4x2	2.3280	2.4560	2.4091	0.0222
**45**	4vac_top3x3_bottom2x3_top3x2_top4x2	2.3332	2.4414	2.4093	0.0207

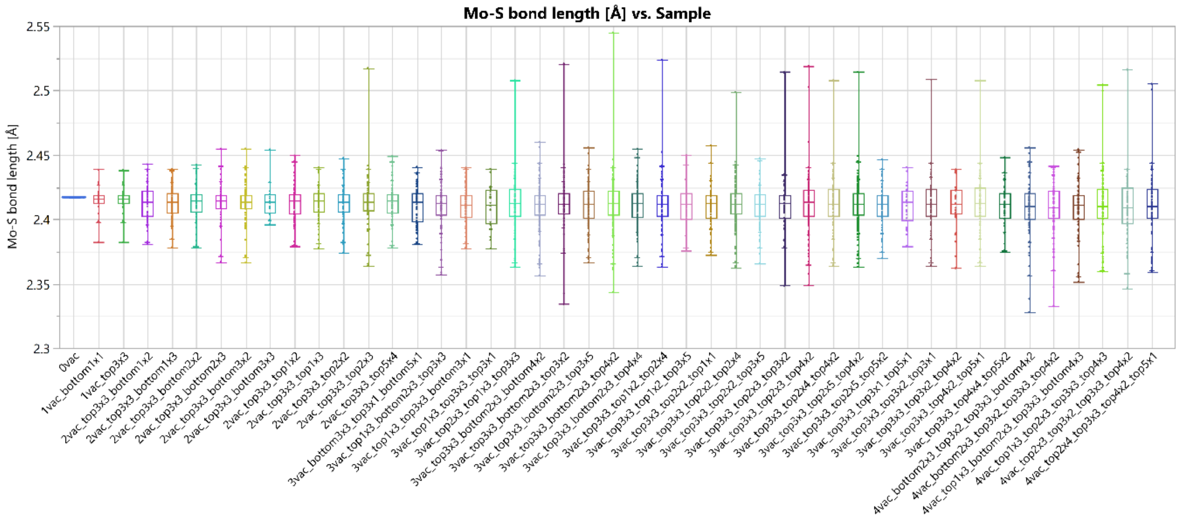

Figure S6 - Box plot showing the variability and central tendency of Mo-S bonds length across different 5x5x1 MoS_2 structures. The box plots visually represent bonds length distributions using boxes, lines, and whiskers, showing the interquartile range (IQR) as the box, the median bond length as a line within the box, and the data range as whiskers extending from the box, when the highest data point represents the longest bond, and the lowest represents the shortest.

Table S4 - Data summary of structure parameters of 5x5x1 MoS_2 models, following structural relaxation.

#	Sample<br>NumberOfVacancies_LayerColxRow	Structure parameters					
		a	b	c	alpha	beta	gamma
**1**	0vac	15.9608	15.9608	14.8682	90.0000	90.0000	120.0000
**2**	1vac_bottom1x1	15.8988	15.8988	14.8790	89.8921	90.1078	119.9998
**3**	1vac_top3x3	15.8974	15.8974	14.8790	89.8691	90.1304	120.0001
**4**	2vac_top3x3_bottom1x2	15.8414	15.8428	14.8790	89.8340	90.2474	119.9971
**5**	2vac_top3x3_bottom1x3	15.8423	15.8428	14.8790	89.9022	90.3226	119.9997
**6**	2vac_top3x3_bottom2x2	15.8426	15.8426	14.8790	89.9030	90.0969	120.0032
**7**	2vac_top3x3_bottom2x3	15.8455	15.8514	14.8790	89.9488	90.0407	119.9877
**8**	2vac_top3x3_bottom3x2	15.8499	15.8445	14.8790	89.8053	90.1856	119.9887
**9**	2vac_top3x3_bottom3x3	15.8406	15.8406	14.8790	90.0000	90.0000	120.0000
**10**	2vac_top3x3_top1x2	15.8474	15.8416	14.8790	89.9783	89.9453	120.0121
**11**	2vac_top3x3_top1x3	15.8515	15.8397	14.8790	89.8652	90.0370	120.0236
**12**	2vac_top3x3_top2x2	15.8494	15.8494	14.8790	89.7016	90.2981	120.0465
**13**	2vac_top3x3_top2x3	15.8621	15.8086	14.8790	89.4098	90.3645	120.1122
**14**	2vac_top3x3_top5x4	15.8490	15.8435	14.8790	89.9260	90.0669	120.0115
**15**	3vac_bottom3x3_top3x1_bottom5x1	15.7770	15.7770	14.8790	90.0572	89.7366	119.9438
**16**	3vac_top1x3_bottom2x3_top3x3	15.8110	15.8034	14.8790	89.8488	90.0515	120.0153
**17**	3vac_top1x3_top3x3_bottom3x1	15.7985	15.7815	14.8790	89.8950	89.9921	120.0357
**18**	3vac_top1x3_top3x3_top3x1	15.7908	15.7908	14.8790	89.9315	90.0684	120.0000
**19**	3vac_top3x3_bottom2x3_bottom4x2	15.8037	15.8004	14.8790	89.9967	89.9538	119.9785
**20**	3vac_top3x3_bottom2x3_top3x2	15.7741	15.8433	14.8790	89.9366	90.1075	120.1459
**21**	3vac_top3x3_bottom2x3_top3x5	15.7879	15.8022	14.8790	90.0795	89.9904	120.0095
**22**	3vac_top3x3_bottom2x3_top4x2	15.7661	15.7736	14.8790	89.9653	90.0664	119.7526
**23**	3vac_top3x3_bottom2x3_top4x4	15.7905	15.7987	14.8790	89.9967	89.9748	120.0307
**24**	3vac_top3x3_top1x2_top2x4	15.7717	15.7717	14.8790	90.0229	90.0033	119.8001
**25**	3vac_top3x3_top1x2_top3x5	15.7862	15.7937	14.8790	89.8419	90.2282	119.9850
**26**	3vac_top3x3_top2x2_top1x1	15.7935	15.7935	14.8790	89.5457	90.4520	120.0638
**27**	3vac_top3x3_top2x2_top2x4	15.7658	15.7848	14.8790	89.9283	90.0935	119.8416
**28**	3vac_top3x3_top2x2_top3x5	15.7820	15.8029	14.8790	90.0009	90.0608	120.0438
**29**	3vac_top3x3_top2x3_top1x3	15.8501	15.7402	14.8790	89.1009	90.5026	120.2326
**30**	3vac_top3x3_top2x3_top3x2	15.7934	15.7934	14.8790	89.8068	90.1900	120.0007
**31**	3vac_top3x3_top2x3_top4x2	15.8041	15.7363	14.8790	89.8771	90.0794	119.8820
**32**	3vac_top3x3_top2x4_top4x2	15.7392	15.7392	14.8790	90.0723	89.9276	119.5297
**33**	3vac_top3x3_top2x5_top4x2	15.7604	15.7709	14.8790	89.9834	89.9938	119.7476
**34**	3vac_top3x3_top2x5_top5x2	15.7794	15.7794	14.8790	90.0378	89.9620	119.8933
**35**	3vac_top3x3_top3x1_top5x1	15.7918	15.7917	14.8790	90.0480	90.1049	120.0000
**36**	3vac_top3x3_top3x2_top3x1	15.7379	15.8466	14.8790	90.0656	89.9988	120.2287
**37**	3vac_top3x3_top3x2_top4x2	15.7961	15.7983	14.8790	89.7925	90.2937	119.9955
**38**	3vac_top3x3_top4x2_top5x1	15.7387	15.7387	14.8790	90.1175	89.7105	119.5362
**39**	3vac_top3x3_top4x4_top5x2	15.7889	15.7886	14.8790	89.7062	90.2324	120.0052
**40**	4vac_top1x3_bottom2x3_top3x3_bottom4x3	15.7725	15.7561	14.8790	89.9245	90.0618	120.0347

#	Sample NumberOfVacancies_LayerColxRow	Structure parameters					
		a	b	c	alpha	beta	gamma
**41**	4vac_top1x3_top2x3_top3x3_top4x3	15.8254	15.6453	14.8790	88.5766	90.7710	120.3817
**42**	4vac_top2x3_top3x3_top3x2_top4x2	15.7669	15.7074	14.8790	89.9988	90.0846	119.8768
**43**	4vac_top2x4_top3x3_top4x2_top5x1	15.6453	15.6453	14.8790	90.1450	89.6294	119.2421
**44**	4vac_top3x3_bottom2x3_top3x2_bottom4x2	15.7300	15.8272	14.8790	89.9916	90.0975	120.2047
**45**	4vac_top3x3_bottom2x3_top3x2_top4x2	15.7485	15.7633	14.8790	90.0425	89.9434	120.0271

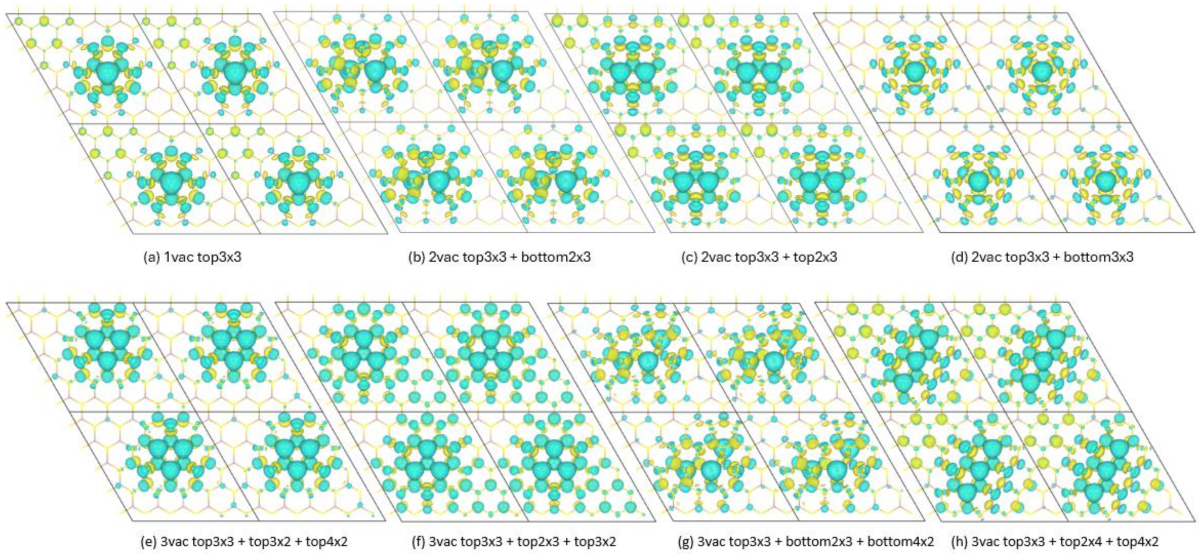

Figure S7 - Examples of charge density difference (CDD) between MoS_2 monolayers with selected S vacancies structures related to the pristine structure. (a) with one sulfur vacancy (2%), (b)-(d) with two sulfur vacancies (4%), and (e)-(h) with three sulfur vacancies (6%). The charge densities were calculated using DFT, by solving the Kohn-Sham equations self-consistently. The big blue surface around the vacancies is caused by the reduction of charge due to the absent S atom. The comparison of the CDDs shows that the surface charge distribution exhibits significant variability for samples with the same vacancy concentration but different arrangements.

Charge transport calculations

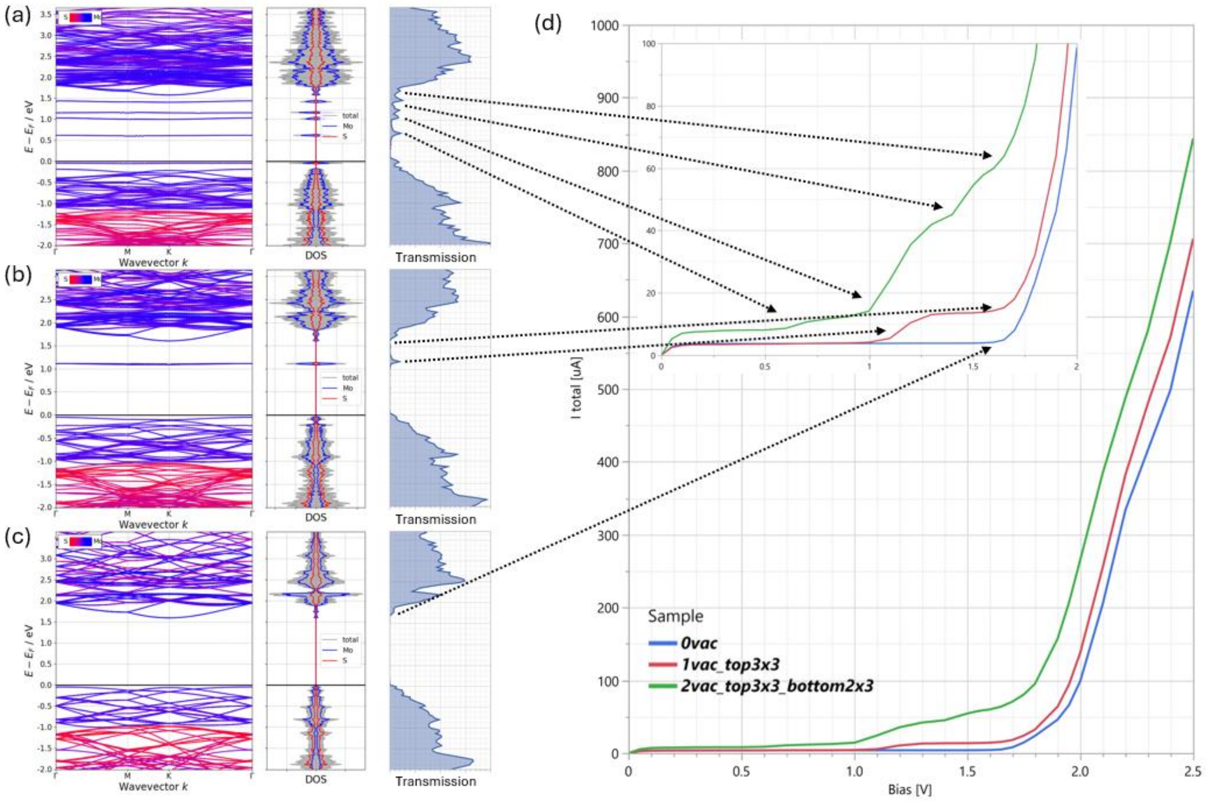

Figure S8 – (a), (b), and (c) DFT calculations of band structure on the left side, DOS in the middle, and transmission functions on the right side for MoS_2 monolayers with two, one and zero vacancies, respectively. (d) calculated current-voltage (IV) plots at 300°K. The band structure (BS) and density of states (DOS) results derived from DFT calculations provide a lot of insight on the contribution of vacancies when they are presented alongside the transmission functions and current voltage (IV). Increasing the vacancies concentration in MoS_2 monolayers leads to a higher current, primarily due to changes in the transmission function and DOS. For example, the slope of the current curve of pristine monolayers starts to increase around 1.6 V, consistently to the calculated band gap energy of the structure (1.59 eV). Similarly, the IV curve of model with one vacancy shows a current increase around 1.1 V, corresponding to the mid-gap state induced by the sulfur absence and around 1.6 V, the edge of the conduction band. Hence, the source of the rise in current due to vacancies are mid gap states that reduce the band gap and increase the transmission function.

Table S5 – Results summary of the current calculation as function of bias, at T=300K for 5x5x1 MoS_2 structures with different concentrations and arrangements of sulfur vacancies.

#	Sample<br>NumberOfVacancies_LayerColxRow	I total [uA] vs. Bias [V]					
		**1.5**	**1.6**	**1.7**	**1.8**	**1.9**	**2**
**1**	0vac	6.43	6.76	8.51	17.97	35.42	85.95
**2**	1vac_bottom1x1	41.71	43.57	49.22	67.70	102.69	181.78
**3**	1vac_top3x3	33.37	34.80	41.49	60.05	89.29	157.30
**4**	2vac_top3x3_bottom1x2	98.28	101.28	106.66	126.03	163.52	235.71
**5**	2vac_top3x3_bottom1x3	152.42	156.23	162.50	180.32	214.95	288.40

#	Sample NumberOfVacancies_LayerColxRow	I total [uA] vs. Bias [V]					
		1.5	1.6	1.7	1.8	1.9	2
6	2vac_top3x3_bottom2x2	104.13	107.06	111.16	122.57	148.48	211.17
7	2vac_top3x3_bottom2x3	175.59	196.64	219.82	256.09	338.53	464.50
8	2vac_top3x3_bottom3x2	141.51	156.44	177.17	212.41	287.45	408.38
9	2vac_top3x3_bottom3x3	47.27	49.90	57.58	86.49	120.65	185.55
10	2vac_top3x3_top1x2	133.12	137.59	142.23	155.60	188.02	264.32
11	2vac_top3x3_top1x3	155.36	159.65	171.88	197.37	254.94	356.90
12	2vac_top3x3_top2x2	107.85	111.11	119.03	139.30	178.52	262.92
13	2vac_top3x3_top2x3	134.75	150.91	161.78	184.42	226.84	318.37
14	2vac_top3x3_top5x4	107.19	109.51	114.05	129.30	159.42	224.51
15	3vac_bottom3x3_top3x1_bottom5x1	254.33	259.52	267.55	291.90	344.68	442.15
16	3vac_top1x3_bottom2x3_top3x3	315.13	347.69	398.42	482.58	601.71	746.91
17	3vac_top1x3_top3x3_bottom3x1	249.48	253.50	260.67	282.56	333.54	423.86
18	3vac_top1x3_top3x3_top3x1	256.76	266.45	294.37	358.07	461.37	597.14
19	3vac_top3x3_bottom2x3_bottom4x2	309.83	340.07	374.92	440.89	561.56	710.55
20	3vac_top3x3_bottom2x3_top3x2	256.05	300.51	344.53	399.13	501.56	640.03
21	3vac_top3x3_bottom2x3_top3x5	312.93	333.60	351.89	390.35	476.87	615.67
22	3vac_top3x3_bottom2x3_top4x2	301.48	336.93	366.39	412.10	505.99	649.03
23	3vac_top3x3_bottom2x3_top4x4	279.01	300.38	312.22	336.56	392.69	502.18
24	3vac_top3x3_top1x2_top2x4	258.88	274.25	283.40	301.15	342.62	434.39
25	3vac_top3x3_top1x2_top3x5	251.88	257.69	266.51	305.59	384.65	497.82
26	3vac_top3x3_top2x2_top1x1	212.35	215.84	220.79	236.88	277.86	366.59
27	3vac_top3x3_top2x2_top2x4	275.60	288.08	306.23	345.11	425.46	555.57
28	3vac_top3x3_top2x2_top3x5	238.79	244.20	254.72	279.23	341.03	453.05
29	3vac_top3x3_top2x3_top1x3	245.71	266.96	298.02	331.58	404.64	530.96
30	3vac_top3x3_top2x3_top3x2	171.41	211.60	231.13	255.80	308.32	411.54
31	3vac_top3x3_top2x3_top4x2	218.71	251.66	272.15	299.46	357.78	470.45
32	3vac_top3x3_top2x4_top4x2	240.93	261.77	291.78	326.52	398.11	518.47
33	3vac_top3x3_top2x5_top4x2	255.26	272.04	281.39	300.72	342.69	432.97
34	3vac_top3x3_top2x5_top5x2	217.68	223.67	233.08	261.98	323.98	436.21
35	3vac_top3x3_top3x1_top5x1	252.39	259.70	281.49	327.03	416.25	539.64
36	3vac_top3x3_top3x2_top3x1	226.50	244.90	276.16	312.87	384.96	505.94
37	3vac_top3x3_top3x2_top4x2	143.33	170.76	193.59	242.34	305.46	411.35
38	3vac_top3x3_top4x2_top5x1	236.06	257.80	290.14	327.40	403.61	529.14
39	3vac_top3x3_top4x4_top5x2	196.57	199.89	205.25	222.30	270.38	368.24
40	4vac_top1x3_bottom2x3_top3x3_bottom4x3	455.28	487.33	547.04	649.97	795.81	969.85
41	4vac_top1x3_top2x3_top3x3_top4x3	362.81	380.50	403.16	450.92	542.52	677.62
42	4vac_top2x3_top3x3_top3x2_top4x2	228.02	274.79	339.77	380.16	452.53	575.60
43	4vac_top2x4_top3x3_top4x2_top5x1	400.25	416.65	438.88	486.61	583.77	727.05
44	4vac_top3x3_bottom2x3_top3x2_bottom4x2	395.67	460.32	529.35	621.33	733.95	869.81
45	4vac_top3x3_bottom2x3_top3x2_top4x2	325.85	383.17	468.45	581.63	735.03	906.74

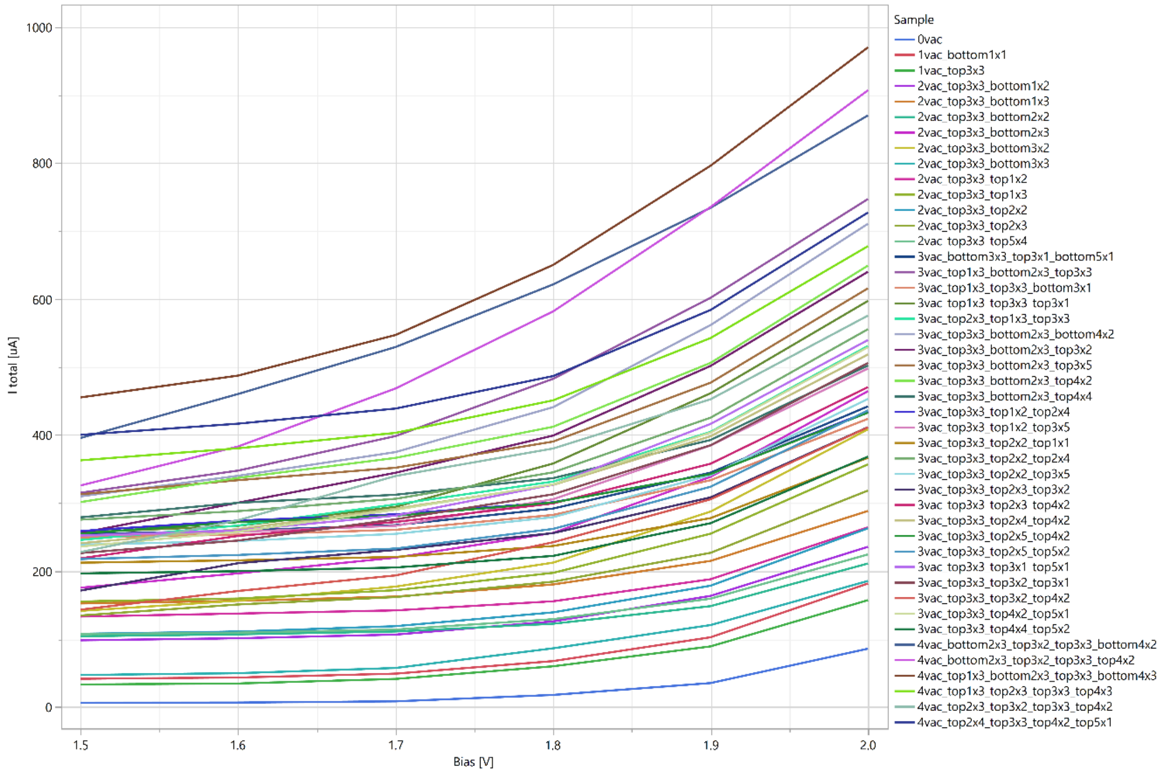

Figure S9 – Current as a function of bias plots @ T=300K for MoS_2 5x5x1 structures with different concentrations and arrangements of sulfur vacancies.

Workflow for charge transport calculation

This section details the main Python scripts and workflow used to calculate electron charge transport. All scripts are available in the Supplementary Information of the publication by Elbaz, Y, et al,[3] with changes to the system type and parameter values according to the description below. In addition to the parameters listed below, we also modified the codes to enable automated execution across all relevant folders for this project. The following is a summary of the steps and key parameters for each script.

1. **'bandsclass_create.py'**: This script processes band structure data obtained from VASP and creates a Python object for tight-binding (TB) fitting.
2. **'multipleTtrainV4.py'**: This is a full TB fitting script that fits a TB Hamiltonian to the DFT band structure. Key parameters include:
    a. The number of bands below and above the VBM to be considered for the fitting, 100 in this work.
    b. The upper limit for the k-points to be used in the fitting process (`klim=120`), and the step size for selecting k-points (`step=1`).
    c. Parameters related to the fitting algorithm: learning rate (lr=3), and number of training steps (`epochs=500`).
    d. In the 'TBHfitting.py' module we define the R vectors.
3. **BlockTransport.py**: This script calculates electron charge transport properties using the NEGF formalism and the Landauer formula. Key parameters include:
    a. The number of unit cells (layers) to consider (`N = 4`).
    b. A small imaginary value for energy broadening (`eta = 0.005`).
    c. Convergence criterion for self-energy calculations (`delta = 1e-15`).
    d. An array of chemical potential values (related to applied bias) for current calculations: `mus = np.array([1.5, 1.6, 1.7, 1.8, 1.9, 2])`. When mu1 was consistently zero (`mu1 = 0`), mu2 was assigned values from the `mus` array (`mu2 = mu`).
    e. The temperatures of the left and right electrodes (for Fermi-Dirac distribution of electrons): `T1 = T2 = 300`.
    f. The `bulk_transport6` class within transport_mod_extensionv2.py is initialized with the on-site Hamiltonian (`H00`, representing interactions within an atom) and the coupling Hamiltonian (`H01`, representing interactions between atoms)."
    g. The slices and coupling matrices for the Hamiltonian of the system were changed to: `sliceBlock_up = np.block([[H0up]])` and `sliceBlock_dn = np.block([[H0dn]])`
    h. The slice coupling blocks were changed to: `sliceCouplingBlock_up = np.block([[H100up]])` and `sliceCouplingBlock_dn = np.block([[H100dn]])`

Design of Experiments (DOE) and data analysis

Table S6 - The full factorial 5x2x2 design matrix used for examination of the main effects and all possible interactions between the three factors (defects concentration - zero to eight percent sulfur vacancies, layer occupancy – single or double sulfur atomic layers, and defect planar arrangement – line or cluster). For each pattern in the table the current was calculated for six bias values between 1.5V and 2V.

Pattern	Defects concentration [%]	Layer occupancy	Defect planar arrangement	Related sample
**112**	0	One layer	Cluster	0vac
**111**	0	One layer	Line	0vac
**122**	0	Two layers	Cluster	0vac
**121**	0	Two layers	Line	0vac
**212**	2	One layer	Cluster	1vac_top3x3
**211**	2	One layer	Line	1vac_top3x3
**222**	2	Two layers	Cluster	1vac_top3x3
**221**	2	Two layers	Line	1vac_top3x3
**312**	4	One layer	Cluster	2vac_top3x3_top2x3
**311**	4	One layer	Line	2vac_top3x3_top2x3
**322**	4	Two layers	Cluster	2vac_top3x3_bottom2x3, 2vac_top3x3_bottom3x2
**321**	4	Two layers	Line	2vac_top3x3_bottom2x3, 2vac_top3x3_bottom3x2
**412**	6	One layer	Cluster	3vac_top3x3_top2x3_top3x2, 3vac_top3x3_top3x2_top4x2
**411**	6	One layer	Line	3vac_top2x3_top1x3_top3x3, 3vac_top3x3_top3x2_top3x1, 3vac_top3x3_top2x4_top4x2, 3vac_top3x3_top4x2_top5x1
**422**	6	Two layers	Cluster	3vac_top3x3_bottom2x3_top3x2
**421**	6	Two layers	Line	3vac_top1x3_bottom2x3_top3x3
**512**	8	One layer	Cluster	4vac_top2x3_top3x2_top3x3_top4x2
**511**	8	One layer	Line	4vac_top1x3_top2x3_top3x3_top4x3, 4vac_top2x4_top3x3_top4x2_top5x1
**522**	8	Two layers	Cluster	4vac_bottom2x3_top3x2_top3x3_bottom4x2, 4vac_bottom2x3_top3x2_top3x3_top4x2
**521**	8	Two layers	Line	4vac_top1x3_bottom2x3_top3x3_bottom4x3

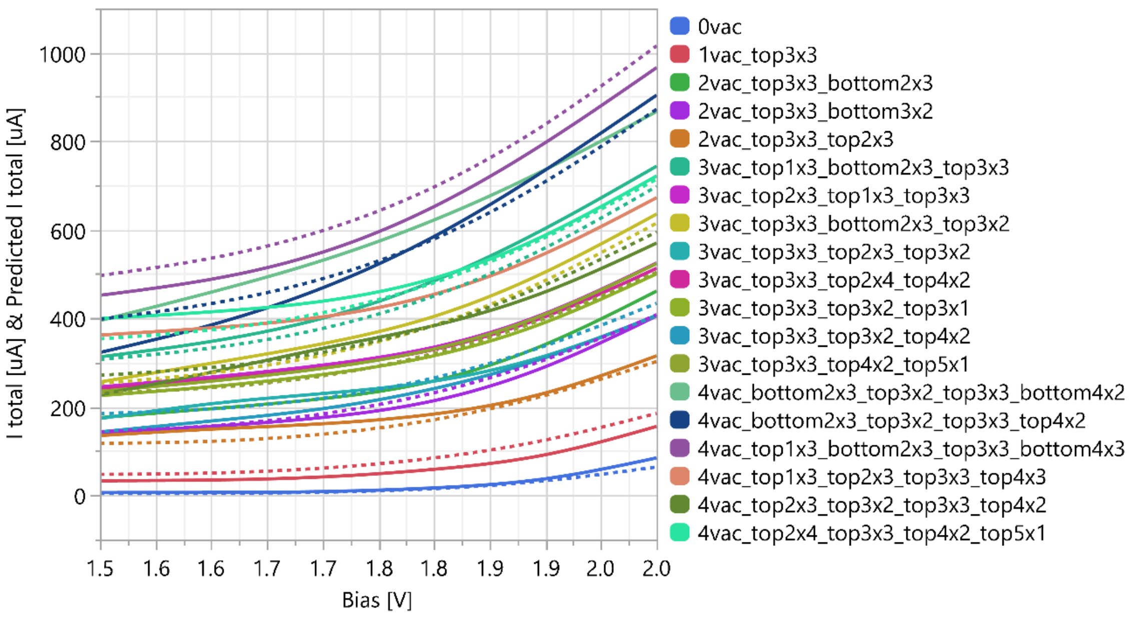

Figure S10 - Predicted and calculated current values versus the applied bias. The solid lines depict the calculated current, whereas the dashed lines represent the predicted current.

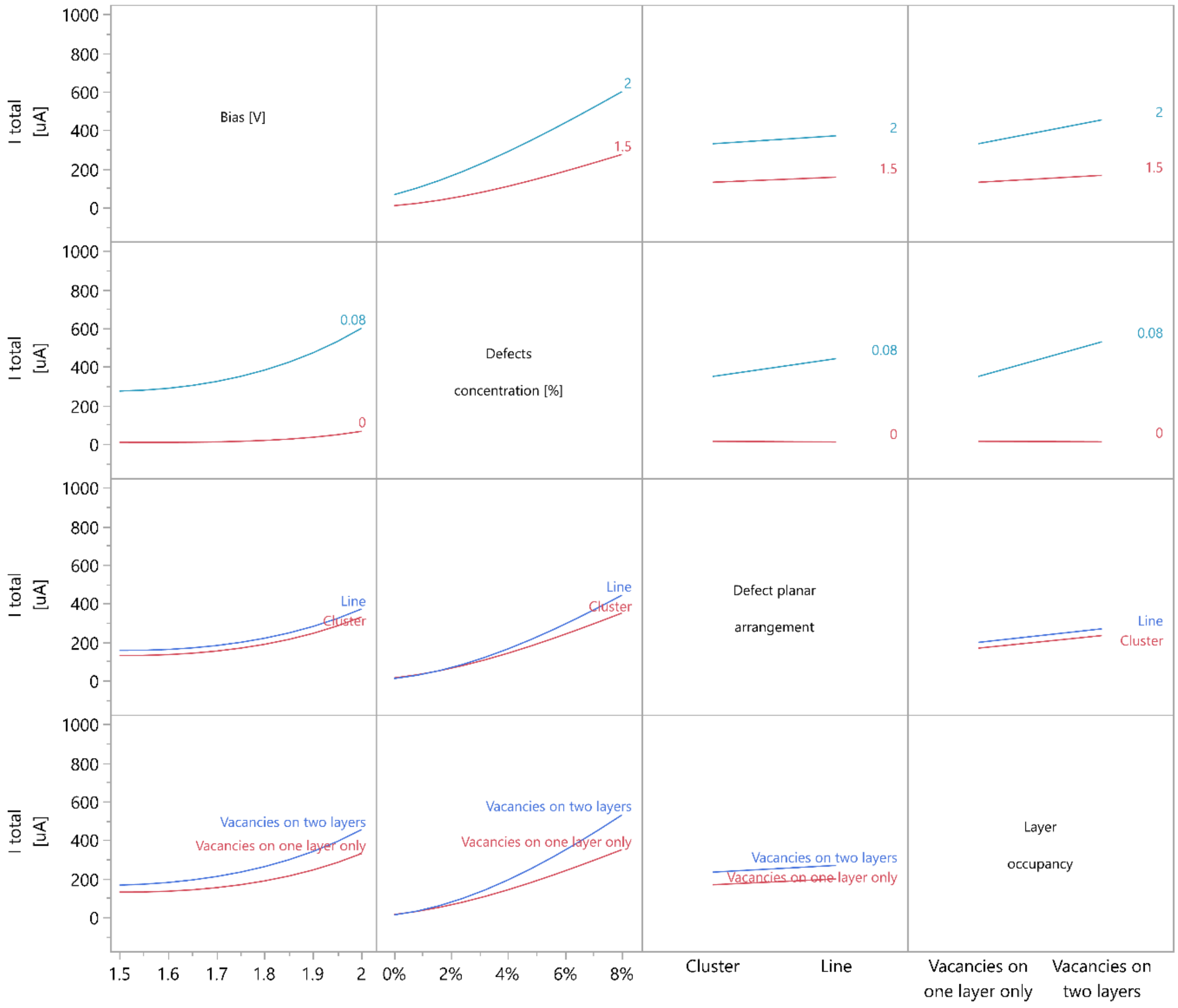

Figure S11 - Interaction profiles plot of the four experimental factors: the applied bias, defects concentration, defect planar arrangement, and layer occupancy. Interaction profiles plot is a useful tool for visualization and understanding complex relationships between variables in statistical analyses. The figure shows a matrix of interaction plots, while each cell of the matrix contains a plot of electric current (I) versus the factor displayed in the diagonal cells in the column where the plot appears. Each plot has two line segments, blue and red, where these lines represent an interaction with the factor displayed in the diagonal cells in the relating row. When blue and red segments are non-parallel, there is evidence of possible interactions between the factors. The current slope increases strongly at higher vacancies concentrations for all three other factors, the slop increases at 8% vacancies concentration compared to pristine structure. We also observe an interaction between bias and layer occupancy, where the current increases with bias at a slightly faster rate when the vacancies are located on both sulfur layers. There is no evidence for other interactions between these factors.

Optimized structures (POSCAR files)

This section provides the optimized structures data used for the DFT calculations. The data is extracted from VASP format POSCAR files, presenting the following information: first line is a structure description, second line has the scaling factor, lines three to five have the lattice vectors, line six shows the elements in the structure, line seven has the number of amount element, line eight specifies the coordinate type, and the following lines have the atomic positions of the elements.

POSCAR_0vac

```
0vac
  1.00000000000000
   15.9608001886909054   0.0000000510296350   0.0000000002706322
   -7.9804000407791662  13.8224583529219043   0.0000000026515711
    0.0000000058534747   0.0000000117230697  14.8681791621172295
  Mo   S
   25   50
Direct
 0.0666665347801043 0.1333330723566633 0.2499999811092550
 0.0666665639280808 0.3333327448225774 0.2499999809790410
 0.0666662782589995 0.5333331571758464 0.2499999812328113
 0.0666665923522061 0.7333338437810042 0.2499999811607339
 0.0666665542739722 0.9333334657762578 0.2499999808875373
 0.2666663208912610 0.1333331505466901 0.2499999812453808
 0.2666661818693896 0.3333327421155730 0.2499999813529783
 0.2666664193144683 0.5333328457617341 0.2499999809804336
 0.2666664226641231 0.7333335999840074 0.2499999806160815
 0.2666661760585853 0.9333334308039767 0.2499999811441995
 0.4666666289840578 0.1333334735746803 0.2499999810840805
 0.4666666306850544 0.3333331282508283 0.2499999811515110
 0.4666668760564647 0.5333331494634166 0.2499999810110651
 0.4666671812580887 0.7333336021301733 0.2499999812804532
 0.4666668577449897 0.9333337152512513 0.2499999811229188
 0.6666668744724689 0.1333334811914924 0.2499999810756606
 0.6666666810342150 0.3333333295685463 0.2499999808138256
 0.6666668876088622 0.5333333867442249 0.2499999812086955
 0.6666672732470076 0.7333338403416505 0.2499999813109355
 0.6666672499364878 0.9333334260916075 0.2499999813408706
 0.8666668248721408 0.1333331471977885 0.2499999808197231
 0.8666665184367446 0.3333331346586874 0.2499999811361135
 0.8666665360229828 0.5333334018813218 0.2499999812972220
 0.8666668711513879 0.7333337029548446 0.2499999807345077
 0.8666669318021434 0.9333334575891215 0.2499999810743319
 0.1333328941440755 0.0666664527749603 0.3551829159139146
 0.1333326614812407 0.2666653132005337 0.3551831056486066
 0.1333320131583449 0.4666653408224164 0.3551831643684551
 0.1333320157020097 0.6666666875696450 0.3551831757886958
 0.1333326529759091 0.8666673512914116 0.3551831046257519
 0.3333333113656565 0.0666673288979993 0.3551830342722013
 0.3333333041093525 0.2666659684242489 0.3551830381626786
 0.3333333284058853 0.4666653361383624 0.3551831624068029
 0.3333333403895509 0.6666666785663509 0.3551830389655635
 0.3333333145024469 0.8666679879494410 0.3551831663422291
 0.5333339894784643 0.0666679721648222 0.3551830044202404
```

```
 0.5333337591745178 0.2666668666644796 0.3551828794744338
 0.5333340006683187 0.4666660173634583 0.3551829917129297
 0.5333346828354664 0.6666666896725317 0.3551831696818368
 0.5333346656936442 0.8666679788022265 0.3551831720810981
 0.7333340204187664 0.0666673296260853 0.3551830362534076
 0.7333331302974031 0.2666668615028200 0.3551828930679974
 0.7333331397344054 0.4666662601357316 0.3551828788504707
 0.7333340552147334 0.6666667162698801 0.3551830366439432
 0.7333346982890276 0.8666673362327586 0.3551831023860785
 0.9333335587064013 0.0666664505068510 0.3551829168075571
 0.9333326658200107 0.2666659632377630 0.3551830395781508
 0.9333320225903421 0.4666660280603594 0.3551829952629930
 0.9333326897634393 0.6666667116111995 0.3551830460505130
 0.9333335706694612 0.8666671129417765 0.3551829199151086
 0.1333328943785901 0.0666664532911128 0.1448170585960327
 0.1333326615669108 0.2666653129149381 0.1448168700834600
 0.1333320131524687 0.4666653403471983 0.1448168118642457
 0.1333320155408941 0.6666666879568766 0.1448167991264455
 0.1333326530348558 0.8666673517001584 0.1448168725169481
 0.3333333118037061 0.0666673294954592 0.1448169417581440
 0.3333333046113793 0.2666659686222559 0.1448169392803607
 0.3333333285758258 0.4666653357778401 0.1448168127781955
 0.3333333394709044 0.6666666783037414 0.1448169359562925
 0.3333333142358157 0.8666679880508070 0.1448168078713294
 0.5333339898903375 0.0666679716497356 0.1448169716166205
 0.5333337584866698 0.2666668660175588 0.1448170951596666
 0.5333340005616165 0.4666660179259026 0.1448169833901076
 0.5333346829464887 0.6666666901681992 0.1448168063205628
 0.5333346664826095 0.8666679788800238 0.1448168052410281
 0.7333340195306945 0.0666673293662186 0.1448169388064144
 0.7333331310397995 0.2666668622903288 0.1448170825982018
 0.7333331402383152 0.4666662600020857 0.1448170965114315
 0.7333340546705855 0.6666667156154631 0.1448169401495676
 0.7333346984175222 0.8666673363379616 0.1448168729867163
 0.9333335579098971 0.0666664503201844 0.1448170599891583
 0.9333326662020980 0.2666659633022732 0.1448169346488228
 0.9333320230274396 0.4666660281921153 0.1448169821105338
 0.9333326899289247 0.6666667115087677 0.1448169291866179
 0.9333335708381441 0.8666671133891697 0.1448170542113019
```

POSCAR_1vac_bottom1x1

```
1vac_bottom1x1
1.0
     15.8987483978       0.0000000000       0.0000000000
     -7.9493338637      13.7687432883       0.0000000000
     -0.0280050817       0.0161733716      14.8789683792
   Mo   S
   25  49
Cartesian
    0.004793388      1.824048345      2.754965859
   -1.595093739      4.592508041      2.738244562
   -3.192904603      7.362175390      2.754951331
   -4.774876146     10.154160943      2.841672771
```

-6.317629256	12.826675129	2.841659564
3.174591393	1.801108625	2.795884582
1.584661892	4.584616307	2.755549263
-0.012337723	7.350487133	2.755598293
-1.627973712	10.119092817	2.795982312
-3.231787168	12.826860617	2.841672771
6.344489473	1.824296305	2.754881831
4.764286481	4.584688419	2.755450048
3.174349853	7.346460623	2.756879505
1.577526609	10.104205262	2.755598458
-0.022870455	12.852817230	2.754951496
9.530694514	1.836896922	2.731744074
7.944158907	4.592601035	2.738102921
6.361083304	7.350411470	2.755450048
4.771341053	10.104088672	2.755549428
3.174637024	12.853896320	2.738244562
12.717391678	1.836764485	2.731839327
11.123937198	4.596463223	2.731743909
9.541754529	7.362103095	2.754881666
7.976893748	10.118912982	2.795884582
6.372136066	12.852574324	2.754965859
1.567645576	0.905065965	4.369110432
-0.012595089	3.669706943	4.314840259
-1.603207575	6.424373379	4.314850164
-3.207388887	9.175119736	4.369131893
-4.774707799	11.935866825	4.423333060
4.781714094	0.905294163	4.369060577
3.174541471	3.673087211	4.330964614
1.580965729	6.426460105	4.321022632
-0.006861356	9.183139921	4.331077531
-1.600171297	11.958892847	4.369132223
7.949384438	0.924106958	4.311223282
6.361679918	3.669951802	4.314734936
4.767794494	6.426481753	4.320930515
3.174407181	9.186372324	4.321022962
1.584135173	11.944995519	4.314850494
11.124014634	0.924184936	4.299627165
9.536827834	3.673070297	4.299549576
7.951962163	6.424392129	4.314734606
6.355685444	9.182972380	4.330964614
4.765053121	11.944808392	4.314840259
14.298703916	0.923789947	4.311296579
12.711025670	3.672959179	4.299627165
11.123785805	6.422313220	4.311222952
9.556251071	9.175007225	4.369060246
7.949422591	11.958590812	4.369110432
1.633098325	0.841172468	1.228605614
0.007615491	3.660697461	1.180418352
-1.600860870	6.446425455	1.180438162
-3.229904168	9.263833223	1.228634421
4.716197509	0.841386488	1.228583163
3.174445547	3.637418693	1.205572520
1.582615464	6.427475481	1.189840156
-0.037751397	9.200974488	1.205698809

```
-1.688256676     11.934035565     1.228634586
 7.926447293      0.910779896     1.176633155
 6.341449567      3.660873355     1.180287440
 4.766025647      6.427502858     1.189734255
 3.174352342      9.184436120     1.189840073
 1.566211061     11.931937218     1.180438327
11.124099090      0.915633009     1.163623841
 9.529513021      3.677293377     1.163525864
 7.949708619      6.446451963     1.180287275
 6.386527018      9.200889821     1.205572602
 4.782960689     11.931809990     1.180418434
14.321636139      0.910550176     1.176698363
12.718474327      3.677162174     1.163623841
11.123858998      6.448840687     1.176632907
 9.578837931      9.263700217     1.228583163
 8.037482040     11.933853342     1.228605697
```

POSCAR_1vac_top3x3

```
1vac_top3x3
1.0
    15.8974132538     0.0000000000     0.0000000000
    -7.9487208161    13.7675544388     0.0000000000
    -0.0338676149     0.0196952934    14.8789519446
  Mo   S
  25   49
Direct
   0.067261964     0.133637115     0.249999553
   0.067260116     0.333617061     0.249997437
   0.066668622     0.533322453     0.251176238
   0.066381305     0.733182430     0.251604527
   0.066667780     0.933332205     0.251177847
   0.267762572     0.134498015     0.249964997
   0.269400924     0.334699959     0.247116312
   0.267765641     0.533251047     0.249962002
   0.266819268     0.733180821     0.251605272
   0.266817570     0.933618665     0.251604527
   0.466744751     0.134497970     0.249965325
   0.468644649     0.337310374     0.243944645
   0.468650758     0.531349123     0.243942067
   0.466748893     0.732234299     0.249962047
   0.466677517     0.933331370     0.251176268
   0.666380942     0.133640200     0.249998733
   0.665295005     0.334705055     0.247117206
   0.662689388     0.531355202     0.243944675
   0.665299952     0.730598867     0.247116283
   0.666382909     0.932739794     0.249997392
   0.866667032     0.133332893     0.249921143
   0.866359830     0.333619028     0.249998778
   0.865502179     0.533255279     0.249965280
   0.865502000     0.732237399     0.249964997
   0.866362810     0.932737887     0.249999553
   0.134528413     0.067859776     0.355848134
   0.136284828     0.267916739     0.354104519
   0.134531915     0.466206878     0.355845690
```

0.133305848	0.666145265	0.356992275
0.133305237	0.866693079	0.356992275
0.334253281	0.068045259	0.356082469
0.339365005	0.272529036	0.352449358
0.339369178	0.466384232	0.352447152
0.334258556	0.665739655	0.356079757
0.333853066	0.866692483	0.356992275
0.533789396	0.067858823	0.355848193
0.533610046	0.272530615	0.352450281
0.533613980	0.660628974	0.352447242
0.533791363	0.865466297	0.355845660
0.733326733	0.066437833	0.355199039
0.732079983	0.267918289	0.354106337
0.727467775	0.466388255	0.352450341
0.727469265	0.660633206	0.352449507
0.732081413	0.863713264	0.354104519
0.933560848	0.066437326	0.355200291
0.933560371	0.266671479	0.355199069
0.932139456	0.466208845	0.355848163
0.931953073	0.665744960	0.356082499
0.932138443	0.865469754	0.355848104
0.133509144	0.066493608	0.145221740
0.133243322	0.266843468	0.144054398
0.133510143	0.467456609	0.145219162
0.133307144	0.667046607	0.146253914
0.133305266	0.866696537	0.146253645
0.332953453	0.066345438	0.145415217
0.334266245	0.266251326	0.141383767
0.334270120	0.468467295	0.141381666
0.332955807	0.667045891	0.145414442
0.332955182	0.866694689	0.146253899
0.532543004	0.066494264	0.145220086
0.531529427	0.266250789	0.141383827
0.533180773	0.466821313	0.137600675
0.531534433	0.665731609	0.141381606
0.532545090	0.866491497	0.145219162
0.732887924	0.066669680	0.144802615
0.733153224	0.266848564	0.144053429
0.733751118	0.468472421	0.141383767
0.733750522	0.665735602	0.141383648
0.733158171	0.866758227	0.144054413
0.933334708	0.066666953	0.144804031
0.933332026	0.267113805	0.144802630
0.933507502	0.467458785	0.145220116
0.933656394	0.667048335	0.145415232
0.933508098	0.866492510	0.145221800

POSCAR_2vac_top3x3_bottom1x2

```
2vac_top3x3_bottom1x2
1.0
    15.8414201736     0.0000000000     0.0000000000
    -7.9207139192    13.7206822352     0.0000000000
    -0.06424609125    0.01267597849    14.8788594203
  Mo   S
```

25 48
Cartesian

-0.003893299	1.835536365	2.712813829
-1.596893463	4.596483823	2.731937728
-3.180009504	7.375679797	2.843176038
-4.718036442	10.032979700	2.851068171
-6.338182824	12.791973874	2.751003002
3.165483905	1.839186247	2.731494789
1.598713862	4.598092678	2.688027637
-0.025845224	7.337844356	2.775038966
-1.641794072	10.033046736	2.851039185
-3.180018801	12.772945060	2.801081183
6.315679177	1.839298672	2.731465151
4.740475339	4.633448255	2.639724144
3.199971916	7.297222252	2.640241829
1.563437144	10.048149070	2.732636660
-0.021869479	12.792168459	2.750937538
9.485042061	1.835726008	2.712707654
7.882336545	4.598218188	2.687998163
6.281186485	7.297236191	2.640284658
4.740688400	10.028539041	2.669738647
3.152928394	12.799479218	2.707130529
12.661272598	1.836125111	2.705518849
11.077976269	4.596545150	2.731926654
9.507150749	7.337863983	2.775038315
7.917973557	10.048043631	2.732706032
6.328404035	12.799354978	2.707210486
1.570848323	0.931500226	4.300645549
0.015213868	3.675126621	4.273670879
-1.598550996	6.392286080	4.359674358
-3.179878832	9.143196188	4.438695031
-4.786858976	11.880044553	4.377800341
4.740628120	0.937689086	4.322524464
3.200375959	3.743072636	4.255180123
1.669151858	6.404124156	4.265816525
0.003624734	9.160973974	4.362969370
-1.573038425	11.880141021	4.377760607
7.910363398	0.931710450	4.300578782
6.280626304	3.743210007	4.255153742
3.206450292	9.078443091	4.249551539
1.591846494	11.882657102	4.298646460
11.077802098	0.915367870	4.275078839
9.465813421	3.675261523	4.273626585
7.812070196	6.404194079	4.265816200
6.274900851	9.078430864	4.249597787
4.740625278	11.859568446	4.258254316
14.244709582	0.915236052	4.275142675
12.661222205	3.656961574	4.287451824
11.079852219	6.392330648	4.359670775
9.477932446	9.160924112	4.362998356
7.889553395	11.882500157	4.298734722
1.594573113	0.895866291	1.162437421
-0.016824560	3.680509412	1.144177498
-1.643657192	6.500049349	1.215406676

```
-4.711248356    11.807288700    1.231546272
 4.740609993     0.868745363    1.193647696
 3.176662350     3.650208397    1.114302635
 1.542921538     6.448584310    1.133983872
-0.115242037     9.151091242    1.218886924
-1.648738264    11.807395806    1.231512970
 7.886577148     0.896068318    1.162348019
 6.304321797     3.650302844    1.114290096
 4.740588770     6.409710620    1.053811149
 3.118260342     9.132476520    1.105140634
 1.545283621    11.878717622    1.159574519
11.069245698     0.918287157    1.137743481
 9.497848095     3.680719948    1.144116838
 7.938252348     6.448715177    1.134002273
 6.363099689     9.132434023    1.105174831
 4.740715828    11.890390438    1.127081186
14.253365338     0.918143904    1.137823763
12.661225527     3.687803359    1.152152520
11.124942580     6.500095968    1.215411072
 9.596732550     9.151089630    1.218914933
 7.936067271    11.878560668    1.159652603
```

POSCAR_2vac_top3x3_bottom1x3

```
2vac_top3x3_bottom1x3
1.0
   15.8422489166     0.0000000000     0.0000000000
   -7.9213000531    13.7202755788     0.0000000000
   -0.0837652137    -0.0190322440    14.8787555613
 Mo  S
 25  48
Cartesian
 -0.016276449     1.844509875    2.525866249
 -1.589468765     4.632004279    2.616354988
 -3.134044412     7.285192170    2.632440403
 -4.759858541    10.040955158    2.549558492
 -6.351190458    12.801827499    2.525660651
  3.155645853     1.846368317    2.525059811
  1.569529854     4.602677086    2.526163960
 -0.042720586     7.288794393    2.615936266
 -1.595109512    10.019752410    2.592915604
 -3.183291361    12.798899724    2.549377277
  6.313286599     1.844011566    2.523682785
  4.729574960     4.634488037    2.434052110
  3.180950799     7.289073205    2.436329944
  1.569252651    10.029409662    2.525830427
 -0.014317000    12.795433441    2.542720174
  9.491242387     1.835279914    2.523749010
  7.881295221     4.593995780    2.480797676
  6.275256599     7.285325378    2.417060618
  4.734352977    10.017594708    2.457665786
  3.153047346    12.794145682    2.506712064
 12.668917694     1.841624556    2.525023538
 11.102576020     4.592787528    2.566439208
  9.492194777     7.312443296    2.523881912
```

7.900633142	10.040167778	2.500210590
6.322444455	12.796848024	2.500678829
1.568548086	0.924768633	4.094803508
-0.002470069	3.663716280	4.127080600
-1.578702225	6.399001566	4.208650657
-3.200268518	9.123251301	4.167997025
-4.779164733	11.885575087	4.111599336
4.735710888	0.932169189	4.103317316
3.200528894	3.739245220	4.062103180
1.659724095	6.418433144	4.097393803
0.023315706	9.119373623	4.156260475
-1.594448470	11.891152843	4.128538149
7.903397462	0.930726904	4.098926608
6.275533002	3.746112939	4.047476819
3.209098700	9.065199979	4.041681241
1.590555366	11.869994197	4.094583461
11.080032941	0.905582113	4.084088628
9.459143473	3.676295245	4.083213256
7.813800140	6.405909574	4.042974733
6.273270991	9.063901103	4.028429045
4.740506573	11.846305183	4.051131528
14.245506283	0.901169328	4.082153658
12.683525091	3.649753905	4.141309612
11.061703131	6.417755559	4.149533234
9.467362629	9.135953810	4.094035601
7.890808313	11.869883516	4.078818334
1.571444108	0.924655963	0.956548046
-0.059785848	3.737219423	0.989301806
-3.132315509	9.065122721	1.021826638
-4.751424858	11.871426886	0.971560995
4.735430811	0.903600740	0.968006225
3.141479593	3.665996860	0.924587705
1.478041920	6.418465035	0.955449466
-0.070669224	9.065049955	1.010304944
-1.601890517	11.848273114	0.998756759
7.901327194	0.906322684	0.964470646
6.297897658	3.652562765	0.907820249
4.717309525	6.401536047	0.842189461
3.114090973	9.118426997	0.895424160
1.547579427	11.868907067	0.955735061
11.078888996	0.928481241	0.949631462
9.524282087	3.676002945	0.963914131
7.921719738	6.419438603	0.898560133
6.340675360	9.123055758	0.881953272
4.733408175	11.888201251	0.921840051
14.246976027	0.929303682	0.946237062
12.708566952	3.743193882	1.001067555
11.171611586	6.405362188	1.005138577
9.516457630	9.136902433	0.954977840
7.919655524	11.885008270	0.939352838

POSCAR_2vac_top3x3_bottom2x2

2vac_top3x3_bottom2x2
1.0

```
    15.8425550461       0.0000000000       0.0000000000
    -7.9220320356      13.7196194851       0.0000000000
    -0.0251659419       0.0145652460      14.8789751132
  Mo   S
  25   48
Cartesian
   0.001927599     1.840014961     2.797514163
  -1.573209062     4.597210446     2.820933262
  -3.139564336     7.338172596     2.883075515
  -4.742867064    10.060633703     2.849783358
  -6.339249969    12.805865135     2.818153000
   3.171457608     1.846711378     2.805075103
   1.602684682     4.610123422     2.781504779
   0.014614534     7.378680427     2.919439620
  -1.536868237    10.033383958     2.940247512
  -3.163617987    12.796146847     2.849783358
   6.323309179     1.839248751     2.819676357
   4.739700589     4.635875440     2.731543498
   3.180673983     7.309883056     2.775953507
   1.537914455    10.017282475     2.919439620
  -0.004245108    12.769049662     2.883075515
   9.493146474     1.827288814     2.816864807
   7.883309312     4.594987962     2.774742694
   6.275946908     7.296901094     2.731543666
   4.729591088    10.026427310     2.781504779
   3.152675007    12.783201378     2.820933262
  12.668320026     1.832536091     2.798863754
  11.085126081     4.584853486     2.816864975
   9.489699193     7.323945416     2.819676525
   7.907160884    10.049710551     2.805075271
   6.328043819    12.797866115     2.797514163
   1.581295992     0.934871677     4.377573328
   0.026591585     3.674394514     4.361748644
  -1.547124901     6.390745258     4.450304901
  -3.168830445     9.164975780     4.467056451
  -4.767679190    11.898575832     4.408205329
   4.740827705     0.935759280     4.395255108
   3.202687006     3.739052516     4.341183991
   1.652332287     6.395637672     4.399558727
   0.010656224     9.139977366     4.517480997
  -1.600887571    11.880905373     4.467056114
   7.911584627     0.935653484     4.405802542
   6.284487895     3.743068406     4.344428191
   3.208165325     9.090590673     4.399558727
   1.612520456    11.863759503     4.450305238
  11.089995824     0.913075101     4.377277942
   9.470338692     3.678774130     4.364814536
   7.821583049     6.405564380     4.344428191
   6.284013340     9.076406028     4.341184328
   4.751807751    11.859229961     4.361748644
  14.255951738     0.915974940     4.364210307
  12.675334106     3.659135316     4.377277942
  11.066424479     6.400342594     4.405802878
   9.480802895     9.146159322     4.395255444
```

```
  7.901655897      11.882750946      4.377573328
  1.590944347       0.913485592      1.236903459
 -0.002978762       3.683881254      1.231196252
 -1.537943381       6.503625318      1.305863142
 -3.076974115       9.151211248      1.321783792
 -4.735691895      11.880108766      1.268316736
  4.759550868       0.893759693      1.256263944
  3.177314466       3.669618082      1.196607575
  1.534271235       6.513589670      1.252849342
 -1.543034949      11.808241360      1.321783708
  7.907027686       0.870452105      1.277053922
  6.305274789       3.651362260      1.203397837
  4.708826397       6.427660276      1.166487033
  3.046983135       9.133849108      1.252849342
  1.519358495      11.799362968      1.305863142
 11.067887830       0.898438684      1.242284666
  9.501322751       3.660886554      1.239659835
  7.911394498       6.433420680      1.203398005
  6.331456319       9.133098962      1.196607575
  4.728806202      11.880093689      1.231196252
 14.253905782       0.917155742      1.227789893
 12.676954092       3.685600394      1.242284750
 11.120609993       6.436892591      1.277054006
  9.526536726       9.150946760      1.256264028
  7.925000756      11.885090023      1.236903544
```

POSCAR_2vac_top3x3_bottom2x3

```
2vac_top3x3_bottom2x3
1.0
    15.8455200195     0.0000000000     0.0000000000
    -7.9227658119    13.7294421874     0.0000000000
    -0.0105759605     0.0092574035    14.8789968862
  Mo   S
  25  48
Cartesian
  0.013797170       1.849893046      2.684385865
 -1.548258647       4.599873738      2.726326013
 -3.153382205       7.322117117      2.703595195
 -4.755499824      10.064610360      2.685591991
 -6.341220117      12.816294039      2.677590764
  3.167032730       1.850133474      2.686096391
  1.603654460       4.643706938      2.732443998
  0.065435824       7.306610593      2.774898302
 -1.574997343      10.054633341      2.713477224
 -3.169747169      12.816506349      2.693953725
  6.320355043       1.850299964      2.688001804
  4.730127733       4.643814101      2.640311716
  3.166603916       7.302769781      2.686213234
  1.591099938      10.021546684      2.735175075
 -0.002507920      12.808913230      2.706183044
  9.498281496       1.837583892      2.688146140
  7.882125840       4.600170117      2.646318845
  6.268734856       7.306846423      2.597574505
  4.743274725      10.021450676      2.637422779
```

3.167125278	12.802142984	2.686243609
12.681410137	1.837315276	2.684534857
11.089742831	4.588159250	2.686254695
9.487739816	7.322073061	2.668298273
7.909496305	10.054503249	2.658273903
6.336841338	12.808588707	2.665983354
1.583012861	0.921068668	4.253306533
0.045520938	3.672369090	4.283992683
-1.561061473	6.428578511	4.308088597
-3.178139246	9.151067079	4.270387848
-4.764104652	11.898049694	4.256277948
4.744694692	0.928196452	4.260958781
3.190345845	3.715584483	4.250716023
1.677406177	6.418014903	4.305320715
-0.000082502	9.133343332	4.320093541
-1.590426406	11.898541515	4.276006533
7.912061501	0.933532969	4.263799385
6.289536284	3.744939717	4.227368173
3.251314208	9.061642574	4.268168488
1.596041490	11.891441153	4.276587424
11.092174606	0.915841501	4.252436526
9.472880070	3.687514311	4.236355142
7.824783429	6.418469242	4.206934537
6.295056307	9.085226111	4.202145508
4.761143396	11.858702575	4.226401942
14.268054930	0.910461780	4.244438846
12.674866466	3.666964341	4.262897008
11.071088193	6.414209073	4.257078337
9.486129099	9.146333542	4.242337438
7.910471801	11.883241074	4.237918671
1.589440963	0.928281533	1.110989782
0.044394762	3.744682948	1.145319143
-1.490399861	6.418049457	1.165390719
-3.151369905	9.146247644	1.129120250
-4.746712211	11.898257311	1.115006581
4.751319879	0.921735817	1.118668526
3.143413895	3.715927885	1.121844805
0.039523457	9.084907524	1.170023108
-1.575663607	11.883523339	1.133764168
7.911805147	0.911063615	1.127967446
6.288319832	3.672875255	1.088562048
4.656555648	6.418162791	1.067393317
3.083110148	9.061441974	1.104753291
1.573235559	11.858897292	1.145945707
11.087505304	0.915950213	1.120226845
9.504424630	3.667224698	1.109719470
7.894989579	6.428612224	1.064215264
6.334158342	9.133321524	1.052090040
4.738088106	11.891316456	1.095748031
14.267512880	0.933340635	1.108573761
12.706551249	3.687165310	1.136278630
11.108811102	6.413905123	1.114980973
9.512363149	9.150787529	1.101086247
7.924682503	11.898413081	1.095737167

POSCAR_2vac_top3x3_bottom3x2

```
2vac_top3x3_bottom3x2
1.0
    15.8499288559    0.0000000000    0.0000000000
    -7.9195583135   13.7233224578    0.0000000000
    -0.0481940449    0.03056472534   14.8788940793
  Mo   S
  25   48
Cartesian
   -0.001294276     1.838488229     2.510571466
   -1.578927483     4.587156234     2.530034351
   -3.169122171     7.327997888     2.550005470
   -4.759055671    10.066939190     2.537825285
   -6.344378273    12.814629484     2.522034461
    3.170139717     1.852585428     2.503421245
    1.616818827     4.611238312     2.481908258
    0.041008189     7.341414803     2.578524458
   -1.570096584    10.066622437     2.557240598
   -3.169370374    12.815859084     2.530088245
    6.325018141     1.850851968     2.512793641
    4.729939115     4.646942867     2.443283878
    3.183725445     7.335177235     2.529693520
    1.629360363    10.019583374     2.617840134
    0.006657914    12.799024984     2.547511263
    9.493144730     1.829479423     2.529659497
    7.880326777     4.601692964     2.491064924
    6.268078612     7.310152751     2.484594257
    4.705347991    10.018599772     2.576234990
    3.166810780    12.769977251     2.569485215
   12.671237074     1.826480914     2.528553904
   11.080437040     4.582521504     2.530985485
    9.481399421     7.328664134     2.531890554
    7.905527248    10.060279199     2.530235778
    6.329319047    12.791481197     2.528585519
    1.586318464     0.937928955     4.082911896
    0.034394849     3.677955232     4.070499874
   -1.575261575     6.402592256     4.120854013
   -3.174096562     9.158202068     4.120144049
   -4.761479724    11.906535092     4.100673336
    4.744528858     0.940220696     4.087134043
    3.202489073     3.735200808     4.047648252
    1.702898586     6.384179358     4.111545598
    0.015222094     9.163240855     4.163580105
   -1.589212218    11.905717081     4.114591999
    7.902809330     0.933160906     4.101338739
    6.277037051     3.742477312     4.052135055
    3.204648192     9.068175495     4.147831734
    1.577031975    11.866359376     4.151584787
   11.082976495     0.917447534     4.106518043
    9.466317343     3.679478116     4.079967262
    7.825851887     6.408584794     4.072105873
    6.302035378     9.107756327     4.094823511
    4.766673624    11.853420374     4.127550495
   14.266775434     0.915633880     4.088748473
```

```
  12.675499135      3.663756084      4.095813185
  11.070907542      6.407510732      4.107116303
   9.492548053      9.154249796      4.104700680
   7.917414135     11.896192585      4.097464950
   1.580787496      0.918830780      0.940391940
  -0.004797169      3.681788622      0.940113660
  -1.559073876      6.438900906      0.989703413
  -3.153868694      9.152835218      0.977573811
  -4.752853706     11.891778628      0.959252424
   4.752945347      0.916115818      0.946063370
   3.181626620      3.677050128      0.897720119
   1.618728188      6.528625698      0.948701196
   0.076214601      9.153011008      1.013772858
  -1.572809255     11.886170388      0.973484067
   7.921173674      0.900490597      0.958815847
   6.302418346      3.677663723      0.909721533
   4.694905581      6.486065294      0.912066852
   1.620500185     11.811378883      1.008886813
  11.081840252      0.880705027      0.979615183
   9.497935637      3.662724851      0.953416675
   7.887185315      6.445297285      0.933075142
   6.278592147      9.148418609      0.966244875
   4.704119912     11.818412837      0.989073614
  14.246731675      0.904882594      0.953086231
  12.673573675      3.667277581      0.963914158
  11.090253614      6.420164443      0.971806258
   9.500628282      9.151425669      0.962753391
   7.915398171     11.887833781      0.955387818
```

POSCAR_2vac_top3x3_bottom3x3

```
2vac_top3x3_bottom3x3
1.0
    15.8406305313     0.0000000000     0.0000000000
    -7.9203209071    13.7183940047     0.0000000000
     0.0000039625     0.0000045755    14.8790035248
  Mo   S
  25  48
Cartesian
   0.013813873      1.838304147      2.841145608
  -1.569211266      4.580230905      2.841144254
  -3.168071519      7.315866433      2.841149503
  -4.759149882     10.056096914      2.841153398
  -6.336817892     12.804150139      2.841151874
   3.188780447      1.865261830      2.841183373
   1.636718707      4.603134122      2.841187776
   0.041907323      7.316177152      2.841176768
  -1.576940175     10.056020052      2.841157463
  -3.168112490     12.811835514      2.841162035
   6.315486108      1.865329289      2.841180325
   4.752139761      4.753547176      2.841215210
   3.324836479      7.226020572      2.841209791
   1.605266711     10.024045493      2.841171857
   0.000539336     12.804072460      2.841152721
   9.490413048      1.838317843      2.841140697
```

7.867582183	4.603231426	2.841178631
6.179660059	7.226146495	2.841202678
4.752234618	9.999443137	2.841170672
3.169107606	12.787300208	2.841135447
12.672373732	1.829222380	2.841121392
11.073490223	4.580290596	2.841138834
9.462590557	7.316334146	2.841166269
7.899307307	10.024205759	2.841164237
6.335204492	12.787326373	2.841134939
1.600978825	0.931159908	4.409543154
0.028322321	3.674605403	4.406361113
-1.561068326	6.408316931	4.409517413
-3.168060758	9.143951642	4.411350092
-4.753613102	11.890055519	4.411351447
4.752161461	0.930923292	4.410339087
3.241745482	3.752662364	4.409310131
1.702936450	6.418349456	4.409335195
0.014449906	9.137256478	4.410310975
-1.582622085	11.889990104	4.411355172
7.903259533	0.931127916	4.409517752
6.262528381	3.752776430	4.409315889
3.213329539	9.034490455	4.409317244
1.590020775	11.866187439	4.409515381
11.082373768	0.911205960	4.406848157
9.475940747	3.674655282	4.406350953
7.801530193	6.418483147	4.409311148
6.291255099	9.034649903	4.409287100
4.752174316	11.856623035	4.406344856
14.262394935	0.911224664	4.406836641
12.672397588	3.665206172	4.406850866
11.065531436	6.408397882	4.409506575
9.490136536	9.137412655	4.410321475
7.914368922	11.866332168	4.409530283
1.601001004	0.931172231	1.272834091
0.028353117	3.674621612	1.276017910
-1.561050750	6.408327006	1.272851958
-3.168056874	9.143954765	1.271015299
-4.753606857	11.890061094	1.271019194
4.752158618	0.930938067	1.272061697
3.241754560	3.752677753	1.273127317
1.702954971	6.418343586	1.273092092
0.014457096	9.137248154	1.272074314
-1.582627643	11.889991592	1.271019448
7.903235270	0.931134822	1.272854328
6.262510787	3.752789775	1.273112160
3.213332952	9.034467414	1.273093871
1.590010498	11.866170121	1.272846284
11.082357946	0.911201522	1.275479385
9.475912769	3.674668629	1.276012491
7.801508350	6.418477685	1.273096326
6.291243878	9.034629314	1.273113176
4.752172538	11.856595907	1.276005802
14.262413752	0.911224008	1.275494033
12.672402656	3.665224425	1.275481840

```
  11.065522103     6.408408365     1.272850518
   9.490130979     9.137408419     1.272047133
   7.914378000    11.866320574     1.272824015
```

POSCAR_2vac_top3x3_top1x2

```
2vac_top3x3_top1x2
1.0
     15.8473939896       0.0000000000       0.0000000000
     -7.9237026368      13.7175897175       0.0000000000
      0.0142076073       0.0147070705      14.87898947298
  Mo   S
  25  48
Cartesian
   0.009337851     1.835770077     2.777376273
  -1.583631958     4.595302804     2.755030074
  -3.166927493     7.372241290     2.678924605
  -4.706288558    10.032287330     2.688343843
  -6.327103153    12.790319408     2.777370744
   3.180394602     1.841011432     2.760549549
   1.611066246     4.597544947     2.716764489
  -0.016073439     7.337094644     2.712098855
  -1.627534591    10.032300389     2.688320753
  -3.166929741    12.771902691     2.740598201
   6.333319739     1.840988974     2.760576867
   4.756799629     4.634132416     2.670097738
   3.213448999     7.293143421     2.671335002
   1.579776514    10.045291929     2.758818778
  -0.006707828    12.790333323     2.777386354
   9.504339802     1.835757440     2.777413022
   7.902553474     4.597556014     2.716793758
   6.300001217     7.293145084     2.671362482
   4.756768513    10.022762694     2.736281030
   3.168781588    12.795813541     2.785284420
  12.680526097     1.834961944     2.783470720
  11.097191793     4.595290962     2.755043733
   9.529547647     7.337093874     2.712146498
   7.933742121    10.045273107     2.758801867
   6.344860412    12.795775086     2.785257753
   1.606039267     0.921834116     4.345599220
   0.027493713     3.699168636     4.317153370
  -1.612842001     6.483590694     4.290461382
  -4.706111173    11.809837027     4.309237418
   4.756809753     0.899836243     4.322547639
   3.214758096     3.746818800     4.286338361
   1.640338758     6.421864512     4.267434192
  -0.075856102     9.135300703     4.293815604
  -1.627763371    11.809839460     4.309217580
   7.907633914     0.921799927     4.345648327
   6.298919332     3.746791417     4.286346166
   3.204954225     9.059468988     4.296689816
   1.581641393    11.864747686     4.349069217
  11.089309322     0.919150045     4.353674850
   9.486150536     3.699160497     4.317190769
   7.873089056     6.421871917     4.267481673
```

6.308561733 9.059513151 4.296701523
4.756816722 11.847534898 4.336663082
14.271804002 0.919125781 4.353632248
12.680480825 3.686656689 4.336044856
11.126340622 6.483596424 4.290467886
9.589334858 9.135320330 4.293820157
7.931951477 11.864736218 4.349047428
1.587424508 0.908572814 1.207421416
-0.003477084 3.655105857 1.187299414
-1.604330196 6.403755020 1.142968651
-3.166928431 9.152128070 1.100541508
-4.767231099 11.877559367 1.163767904
4.756842379 0.911377046 1.193214102
3.191581185 3.652193192 1.145264557
1.591565866 6.427147396 1.132549959
-0.003476615 9.176504313 1.150352559
-1.566575274 11.877602694 1.163760343
7.926263315 0.908584072 1.207437921
6.322068818 3.652170744 1.145301387
4.756754220 6.409326460 1.085575071
3.155697382 9.139975712 1.151108430
1.586907211 11.890883169 1.210583602
11.095594110 0.913048328 1.216417881
9.517072752 3.655086262 1.187328765
7.921930534 6.427150301 1.132594513
6.357774339 9.139957732 1.151116153
4.756809079 11.892946324 1.206719530
14.265498540 0.913029004 1.216410645
12.680480948 3.655052610 1.200846714
11.117804717 6.403757110 1.143014587
9.517025758 9.176470004 1.150384755
7.926711684 11.890834078 1.210550187

POSCAR_2vac_top3x3_top1x3

2vac_top3x3_top1x3
1.0
15.8514661789 0.0000000000 0.0000000000
-7.9254858460 13.7142883413 0.0000000000
-0.0096209938 0.0348651997 14.8789595653
Mo S
25 48
Cartesian
-0.002965131 1.857580156 2.740428469
-1.582478232 4.635081043 2.637895993
-3.129919647 7.292556654 2.655703919
-4.751412622 10.047080296 2.759183140
-6.342453149 12.808565008 2.776408962
3.169953085 1.856523657 2.738961200
1.583525522 4.618321974 2.654112685
-0.032107383 7.293209717 2.646255977
-1.584165529 10.021506848 2.721165364
-3.172299341 12.801269660 2.763962162
6.330347595 1.849676490 2.734024199
4.748467213 4.634574009 2.638323503

3.199838552	7.293120497	2.645507189
1.583286006	10.030832247	2.741091013
-0.000857984	12.796168350	2.758161114
9.509037056	1.840709627	2.734283091
7.899078791	4.598793986	2.685390889
6.296323992	7.292242244	2.655833042
4.750559459	10.022125941	2.718849201
3.168110977	12.796507237	2.757314798
12.687467390	1.850523967	2.734896630
11.117962569	4.599487572	2.685038499
9.508949347	7.318329549	2.725114514
7.917119754	10.046944930	2.758625700
6.339809425	12.802630994	2.764091769
1.584028521	0.961608649	4.327196912
-0.020574175	3.775931551	4.244124853
-3.126003873	9.057681558	4.286954213
-4.748607508	11.878622173	4.342095931
4.752424178	0.941769556	4.317804262
3.186693766	3.774082233	4.243657042
1.584768848	6.408811137	4.223642731
-0.035212289	9.050862906	4.280145002
-1.583236385	11.853523810	4.316005238
7.920459913	0.939241014	4.312828573
6.292752211	3.752315454	4.255469992
3.202910014	9.051256477	4.278564891
1.583868638	11.861257490	4.327484820
11.097466875	0.938889879	4.313157749
9.508597904	3.703045474	4.262146695
7.853780283	6.399770115	4.262807627
6.290333515	9.058194431	4.285704246
4.750438047	11.853449441	4.314268437
14.265619570	0.943067096	4.319358258
12.724220638	3.753255565	4.255495785
11.163846489	6.400440026	4.262643200
9.508421769	9.124195787	4.324773387
7.915358898	11.878482771	4.341909258
1.582665725	0.912949574	1.188187275
-0.019530892	3.653454938	1.108575437
-1.588080598	6.418383663	1.065343875
-3.190367952	9.142371984	1.140381500
-4.763234599	11.890148329	1.201222144
4.748464720	0.910531867	1.182109200
3.186306151	3.653369196	1.107741534
1.583911686	6.451349456	1.074700415
0.011044910	9.139648867	1.132255502
-1.589371791	11.888217432	1.185475197
7.919343329	0.909378563	1.177223220
6.317077534	3.654196466	1.114031269
4.755219822	6.417756277	1.064818676
3.154767555	9.139416254	1.131012063
1.583350540	11.881544779	1.187249961
11.099320029	0.910118883	1.177235793
9.508436622	3.658579103	1.140700198
7.920823791	6.435469408	1.115978278

```
  6.356761780      9.141202237      1.138987417
  4.757532977     11.890318451      1.184518619
 14.270281032      0.912636068      1.183382380
 12.699728756      3.654479568      1.113971947
 11.096987063      6.436024664      1.115548028
  9.508585164      9.161356673      1.184580118
  7.929921867     11.890447733      1.200968411
```

POSCAR_2vac_top3x3_top2x2

```
2vac_top3x3_top2x2
1.0
    15.8494338989     0.0000000000     0.0000000000
    -7.9358657859    13.7195706702     0.0000000000
    -0.0774224679    0.04472987072    14.878734855
  Mo   S
  25   48
Cartesian
  -0.012686969      1.847914324      1.937438737
  -1.590583803      4.606032005      1.906200361
  -3.161173220      7.347620546      1.881728778
  -4.770315500     10.071068548      1.935358035
  -6.367519552     12.814939973      1.957627543
   3.159903934      1.860883602      1.929412788
   1.589237686      4.618637392      1.860423123
  -0.007909059      7.383060590      1.809371417
  -1.565289345     10.044971641      1.842473695
  -3.192647119     12.806235864      1.935358035
   6.312027835      1.847692721      1.908267256
   4.726660123      4.646438651      1.819413930
   3.154914003      7.322316753      1.762911041
   1.518696542     10.029701548      1.809371862
  -0.029475800     12.776971176      1.881729001
   9.484432166      1.834140988      1.909251598
   7.873554296      4.600563168      1.867304162
   6.258182818      7.301605058      1.819413262
   4.711330297     10.031337401      1.860423012
   3.130094430     12.790162051      1.906200584
  12.660147118      1.839613845      1.930485543
  11.074793604      4.591313958      1.909251375
   9.474629496      7.330621521      1.908267256
   7.884931343     10.052555961      1.929412788
   6.307631343     12.805303508      1.937438737
   1.577219582      0.950486846      3.516358594
   0.014090942      3.715160752      3.469135799
  -1.537417283      6.497885768      3.438984430
  -3.104995215      9.150714117      3.460537952
  -4.770247999     11.893620062      3.519978611
   4.748801694      0.935864299      3.500205144
   3.188073273      3.781908771      3.446018688
   1.607798283      6.493144580      3.384303130
  -1.562139104     11.825527383      3.460537952
   7.899753154      0.902626613      3.466527738
   6.271445058      3.748574142      3.433784789
   3.098015719      9.076698565      3.384302908
```

```
   1.519090531    11.796882530    3.438984875
  11.058844697     0.905367824    3.485808589
   9.460478339     3.685211674    3.458874370
   7.808869712     6.413973467    3.433784567
   6.236160334     9.066307869    3.446018020
   4.704715387    11.847187438    3.469135799
  14.243457890     0.926346338    3.507163238
  12.667070866     3.693512058    3.485808366
  11.087675949     6.429454490    3.466527738
   9.481211862     9.140335369    3.500205367
   7.880532571    11.878395321    3.516358817
   1.570481392     0.919682083    0.377389292
  -0.021082112     3.659815596    0.341212671
  -1.590138453     6.413295631    0.293657828
  -3.196699534     9.185998247    0.316029027
  -4.790794568    11.905470771    0.378836101
   4.725505602     0.916506909    0.360815019
   3.165771507     3.657701907    0.298520298
   1.539723734     6.423860500    0.231801328
  -0.039308413     9.164772330    0.240875895
  -1.638598551    11.887242016    0.316028999
   7.898562801     0.920274313    0.337548308
   6.294641478     3.660949634    0.295303872
   4.729869757     6.413867962    0.221288037
   3.123903257     9.170316040    0.231801620
   1.565915975    11.884874061    0.293658189
  11.082654586     0.920143040    0.350537027
   9.492401742     3.666797829    0.332996062
   7.896333978     6.437767824    0.295303621
   6.332509616     9.147803600    0.298519908
   4.735011799    11.905346000    0.341212504
  14.251377934     0.921778084    0.370753698
  12.666202585     3.665503508    0.350537082
  11.071803268     6.421648387    0.337548253
   9.486303747     9.170193350    0.360814991
   7.903824474    11.899652209    0.377389041
```

POSCAR_2vac_top3x3_top2x3

```
2vac_top3x3_top2x3
1.0
    15.8621416092     0.0000000000     0.0000000000
    -7.9310801017    13.6751455119     0.0000000000
    -0.0946619428     0.1222759086    14.8781999452
  Mo   S
  25   48
Cartesian
  -0.000301314     1.864895692    2.184892441
  -1.571688899     4.593509126    2.121139996
  -3.176928897     7.310948645    2.187054152
  -4.772144211    10.050308466    2.238458768
  -6.355843447    12.796630367    2.245367391
   3.158941192     1.908474153    2.198509841
   1.588950102     4.666720418    2.016460505
   0.026454400     7.272177843    2.079387723
```

-1.592744236	10.031212029	2.215124414
-3.188337856	12.796640528	2.245314423
6.318195007	1.864973691	2.184837170
4.728938676	4.666749727	2.016456283
3.158904896	7.228279723	1.983087422
1.580341315	9.968233775	2.153708322
-0.013071070	12.766294471	2.216873896
9.498875467	1.834420605	2.184837170
7.889543902	4.593580909	2.121146649
6.291378507	7.272205207	2.079444401
4.737378724	9.968227991	2.153797881
3.158908776	12.747215117	2.200180510
12.681124681	1.834370493	2.184888859
11.089970779	4.577153842	2.170747919
9.494755749	7.310984029	2.187094838
7.910591651	10.031191474	2.215301231
6.330933098	12.766219587	2.216985973
1.579686991	0.996156252	3.785529162
0.064804900	3.782280675	3.672649088
-1.512538241	6.386615568	3.703475225
-3.172546271	9.113859098	3.790757904
-4.772070200	11.880848058	3.821586088
4.738312165	0.996170225	3.785481312
3.158963900	3.958536505	3.659726926
0.042410711	9.025322419	3.723777861
-1.584851526	11.858450010	3.802263746
7.909348769	0.949237553	3.767578872
6.253104261	3.782332492	3.672656253
3.158846369	8.887690386	3.666602028
1.584878771	11.796453365	3.757834794
11.089998430	0.920020601	3.757782338
9.472397043	3.685859974	3.724790140
7.830395194	6.386682465	3.703532032
6.275333142	9.025298832	3.723883285
4.732919811	11.796448227	3.757903883
14.270703287	0.949168852	3.767649752
12.707639919	3.685854298	3.724793722
11.090006587	6.388333918	3.756946876
9.490489152	9.113864708	3.790845417
7.902844113	11.858469625	3.802369426
1.591385474	0.929855806	0.650823277
-0.005705097	3.646049708	0.533858137
-1.612832074	6.432191824	0.552820386
-3.195636406	9.156702027	0.647010408
-4.772136690	11.884351819	0.677157619
4.726451119	0.929893612	0.650799096
3.158927123	3.654246944	0.503231301
1.555664211	6.436298708	0.434597605
-0.042104422	9.132649937	0.579363466
-1.616113809	11.873783767	0.660464809
7.907036004	0.910779192	0.630582821
6.323540749	3.646148403	0.533816811
4.762235004	6.436343755	0.434623866
3.158897261	9.094800735	0.518971422

```
  1.564570761     11.848288182      0.631305631
 11.089987190      0.903725898      0.624965455
  9.510883179      3.650519808      0.594119423
  7.930623403      6.432268605      0.552840025
  6.359869814      9.132726889      0.579503051
  4.753217288     11.848250458      0.631376895
 14.272920713      0.910681870      0.630667263
 12.669020999      3.650439369      0.594150513
 11.089935974      6.414452596      0.613201362
  9.513372981      9.156742485      0.647073419
  7.933951552     11.873724584      0.660602859
```

POSCAR_2vac_top3x3_top5x4

```
2vac_top3x3_top5x4
  1.0
    0.0000000000   0.0000000000  -15.8490447998
  -13.7192959773   0.0112129655   7.9245178905
    0.0000000000  14.8789933815   0.0173736576
 Mo  S
 25  48
Direct
    0.0659714118   0.1349640042   0.2499997334
    0.0631277784   0.3314697743   0.2469586967
    0.0652045906   0.5304000378   0.2513092587
    0.0660664067   0.7325367927   0.2546058130
    0.0663854480   0.9336026907   0.2540971839
    0.2674680948   0.1347396821   0.2525898324
    0.2685396671   0.3346664607   0.2498092816
    0.2665552795   0.5320926309   0.2529227695
    0.2664822340   0.7325393558   0.2546031842
    0.2667556107   0.9335016608   0.2544042328
    0.4672793150   0.1347406507   0.2525869807
    0.4686083496   0.3372190893   0.2476742309
    0.4684856534   0.5311806202   0.2483804412
    0.4667192399   0.7322368622   0.2541788586
    0.4672267437   0.9336048961   0.2540935524
    0.6689908504   0.1349654943   0.2499948878
    0.6661334038   0.3346686959   0.2498097828
    0.6626971364   0.5311818719   0.2483819785
    0.6654690504   0.7309267521   0.2517862740
    0.6675605774   0.9340562821   0.2529829896
    0.8688545823   0.1377149671   0.2468891417
    0.8683399558   0.3314700723   0.2469586188
    0.8655503988   0.5320934653   0.2529267797
    0.8655324578   0.7322357297   0.2541819331
    0.8665031791   0.9340570569   0.2529852844
    0.1331882924   0.0693371072   0.3575440079
    0.1306722760   0.2678262293   0.3542319027
    0.1280774474   0.4600956738   0.3561944361
    0.1317694932   0.6632941961   0.3588377261
    0.1330814660   0.8666471243   0.3599574902
    0.3342803716   0.0683180019   0.3587729834
    0.3381459713   0.2722093165   0.3558732401
    0.3378444612   0.4656367302   0.3561600819
```

```
0.3327950537  0.6646617055  0.3597549296
0.3338081837  0.8666491508  0.3599556187
0.5363829732  0.0693385154  0.3575404655
0.5343016386  0.2722111046  0.3558718141
0.5333496929  0.6607584358  0.3572177544
0.5347701311  0.8669735193  0.3595162103
0.7392645478  0.0728974715  0.3554964802
0.7373875379  0.2678254545  0.3542306106
0.7280310989  0.4656382799  0.3561624435
0.7276507020  0.6607588530  0.3572198041
0.7328348160  0.8654308915  0.3579911916
0.9338590503  0.0728976727  0.3555004904
0.9322594404  0.4600959122  0.3561961071
0.9321115613  0.6646619439  0.3597601206
0.9324448109  0.8669729233  0.3595194853
0.1334094554  0.0663269907  0.1472373310
0.1339368224  0.2684878707  0.1435893869
0.1340638399  0.4661834836  0.1448206740
0.1332914382  0.6668068767  0.1485237975
0.1330303103  0.8663794398  0.1491555014
0.3330585062  0.0663433299  0.1480950329
0.3347095549  0.2667747736  0.1443527485
0.3348250687  0.4688970744  0.1449155435
0.3333300352  0.6667028665  0.1488489760
0.3331254721  0.8663818240  0.1491527444
0.5326907635  0.0663288012  0.1472321623
0.5318391323  0.2667767108  0.1443521915
0.5334414840  0.4671119750  0.1416967705
0.5318210721  0.6657317877  0.1458380660
0.5329853892  0.8661446571  0.1486349975
0.7342191934  0.0661661476  0.1444010047
0.7343223095  0.2684915662  0.1435876269
0.7338499427  0.4688992202  0.1449181946
0.7336893677  0.6657330393  0.1458393638
0.7330395579  0.8662980795  0.1477143742
0.9317167997  0.0661667660  0.1444023582
0.9334662557  0.2671700120  0.1403791135
0.9318957925  0.4661852122  0.1448228462
0.9331529737  0.6667007804  0.1488512874
0.9329382777  0.8661436438  0.1486382391
```

POSCAR_3vac_bottom3x3_top3x1_bottom5x1

```
3vac_bottom3x3_top3x1_bottom5x1
1.0
    15.7769798485    0.0076979546    0.0288239702
    -7.8817981537   13.6672007881   -0.0026848386
     0.0412109486    0.0095356671   14.8789433973
  Mo   S
  25   47
Cartesian
   0.010929903     1.790042313     2.420574156
  -1.564203936     4.554867758     2.362507545
  -3.147251909     7.299858788     2.317482370
  -4.752714487    10.047726770     2.355064918
```

-6.342991829	12.734605660	2.438852572
3.163957141	1.825297458	2.383204929
1.617696424	4.573049920	2.425948608
0.036155826	7.308478146	2.346642827
-1.536318987	10.047246183	2.271195238
-3.140527784	12.761500005	2.320711631
6.317768216	1.825294007	2.328676415
4.739537655	4.611644035	2.452299118
3.203256695	7.287229515	2.452628760
1.608276648	10.072193992	2.250633467
0.065831071	12.730883018	2.217343148
9.470417114	1.792946373	2.284416186
7.861814243	4.578179332	2.373643523
6.270606135	7.286559455	2.449385331
4.750361371	10.025811418	2.396844921
3.139173578	12.724051652	2.255778745
12.628203272	1.799964108	2.365014666
11.044150085	4.561534649	2.341176459
9.446393671	7.314923925	2.357876022
7.882882215	10.079046439	2.454588219
6.348284664	12.734772350	2.462916104
1.546649912	0.859964184	0.839574954
0.050145920	3.625748202	0.842651036
-1.539071079	6.369155257	0.771326130
-3.176530824	9.131857588	0.749086128
-4.821110413	11.862148734	0.796180499
4.714852483	0.933091894	0.769029544
3.193781130	3.712923719	0.845081676
1.691611031	6.369306107	0.839739244
0.057889357	9.095307737	0.710140516
-1.572603637	11.873329873	0.689091522
7.873552617	0.916012815	0.697087955
6.258826661	3.734849659	0.809562749
3.238347290	9.036272634	0.796840913
1.626625121	11.832559569	0.646265037
11.094284203	0.892464272	0.723445257
9.445564689	3.675486913	0.767652892
7.791824681	6.405829264	0.817642822
6.328283651	9.117899392	0.860114198
4.853713094	11.834568009	0.802185093
14.271736907	0.840286488	0.858008281
12.656612295	3.638311304	0.795022177
11.031608170	6.402527213	0.773002444
9.408992746	9.206732197	0.822876618
1.607410163	0.888859212	3.994295227
-0.003047784	3.650436175	3.969330090
-1.571785975	6.409194455	3.911845536
-3.113298305	9.137506584	3.874106269
-4.700651002	11.861640876	3.948253893
4.767594969	0.896598566	3.914628691
3.193844737	3.636826944	3.999627340
1.583566779	6.428070354	3.988867518
0.051678565	9.211641175	3.856882428
-1.468775841	11.850435308	3.840697060

```
   7.939919277     0.828449423     3.848459269
   6.314266646     3.621627847     3.961463634
   4.737787564     6.393147697     4.038205262
   3.097650379     9.192986403     3.937232279
  10.985266335     0.830623117     3.869829094
   9.467139430     3.609008306     3.892579215
   7.889578263     6.399335248     3.965879945
   6.324411206     9.114222292     4.009237392
   4.642685231    11.869064769     3.942380354
  14.161726467     0.895298133     4.007119964
  12.600499281     3.637681406     3.935455445
  11.043303017     6.389872217     3.914029223
   9.458395124     9.116474611     3.970987567
   7.887551593    11.848650821     4.048484162
```

POSCAR_3vac_top1x3_bottom2x3_top3x3

```
3vac_top1x3_bottom2x3_top3x3
1.0
    15.8109722137     0.0000000000     0.0000000000
    -7.9053550831    13.6840129704     0.0000000000
    -0.0133853851     0.0376129720    14.8789499625
  Mo   S
  25  47
Cartesian
   0.003254232     1.855050924     1.971844881
  -1.563206605     4.637559277     1.912160725
  -3.100063188     7.282566657     1.888005591
  -4.736278727    10.023060082     1.964255468
  -6.326586098    12.779116467     1.984005826
   3.154113864     1.855368010     1.970073884
   1.578545777     4.650296663     1.966239462
   0.021672435     7.282936996     1.959004828
  -1.572760419     9.984930075     1.947121004
  -3.163212298    12.771003401     1.977235693
   6.309478755     1.848580384     1.967352297
   4.720891780     4.637047932     1.911859919
   3.136323871     7.283043627     1.960855095
   1.579005449     9.974284988     2.023150796
  -0.001255679    12.760100852     1.991426711
   9.484270194     1.837157935     1.968905464
   7.875994532     4.592046655     1.919896754
   6.258388690     7.282957931     1.890496459
   4.730625076     9.985580897     1.950327231
   3.159130196    12.760507676     1.993938524
  12.659327795     1.848759847     1.969862337
  11.093485768     4.592179874     1.919842619
   9.484463407     7.302626673     1.940107746
   7.894598338    10.023300217     1.965680064
   6.321394174    12.770923431     1.980447075
   1.579375296     0.942704911     3.551675510
  -0.006601397     3.739771301     3.510885214
  -3.124255779     9.044776886     3.508367279
  -4.738546794    11.846855375     3.550435876
   4.735681491     0.930698255     3.541809998
```

```
  3.164154936    3.738972220    3.510011316
  1.578132708    6.410281560    3.536959412
 -0.070483760    9.013682290    3.565032426
 -1.588411171   11.819810052    3.539213115
  7.897542261    0.934141381    3.545699038
  6.280287774    3.744208996    3.503081676
  3.226554283    9.014136786    3.566720609
  1.577615818   11.838174581    3.575588163
 11.069872706    0.934979035    3.547595063
  9.484658449    3.699203560    3.496405957
  7.832785935    6.396799144    3.490159454
  6.283721796    9.046015100    3.510659972
  4.745961425   11.820520970    3.542638783
 14.232785064    0.931493685    3.544950489
 12.688603460    3.746180476    3.504250741
 11.137389241    6.397522461    3.489133461
  9.485560516    9.107238042    3.533594968
  7.898242107   11.847178077    3.552258109
  1.578238807    0.923139822    0.407297241
  0.019695785    3.705332223    0.389639107
 -1.488597233    6.411687002    0.344245015
 -3.162432416    9.113231706    0.354389018
 -4.746166706   11.864450864    0.405224028
  4.738122674    0.921634135    0.403372150
  3.137365383    3.707211447    0.388514229
  0.076807878    9.044483924    0.393469299
 -1.571056977   11.850808719    0.405584245
  7.899265878    0.905454111    0.410369499
  6.281347140    3.664763018    0.363449429
  4.647325343    6.412452094    0.346354600
  3.083422274    9.044447618    0.395239652
  1.580084459   11.818353326    0.441148794
 11.070309899    0.904756878    0.412357279
  9.484881283    3.653682628    0.373271479
  7.878245604    6.420168366    0.343288263
  6.319192514    9.113923806    0.356823656
  4.729665326   11.850974591    0.409251589
 14.230209299    0.920478131    0.406013743
 12.688243332    3.663330351    0.364573864
 11.089389550    6.419912810    0.342377348
  9.483553422    9.134972392    0.388931602
  7.903064929   11.864398973    0.406637306
```

POSCAR_3vac_top1x3_top3x3_bottom3x1

```
3vac_top1x3_top3x3_bottom3x1
1.0
    15.7984695173    -0.0000128984     0.0009094308
    -7.8992352204    13.6622135587     0.0120906205
     0.0012020424     0.0190390818    14.8789912951
  Mo   S
  25  47
Cartesian
 -0.004323088    1.846622058    2.661833995
 -1.576051802    4.615303719    2.565332544
```

-3.105606336	7.264653503	2.608128165
-4.726684415	10.009023809	2.720363146
-6.319170420	12.756511356	2.721036095
3.164478516	1.846631681	2.662007924
1.580051147	4.603893153	2.573135637
-0.020161643	7.278254130	2.595610628
-1.548896283	10.001108499	2.720732360
-3.141703029	12.750197402	2.725099852
6.321239703	1.822763326	2.681149323
4.736177405	4.615311866	2.565690155
3.180221271	7.278251253	2.595770418
1.580032936	10.039086789	2.788923124
0.052363020	12.712364224	2.806348313
9.479319162	1.790729851	2.721563629
7.874806919	4.570564985	2.633195703
6.265673049	7.264615103	2.608675282
4.709000714	10.001085351	2.721076395
3.107716033	12.712334846	2.806500572
12.637370799	1.822704983	2.681523774
11.083853321	4.570569426	2.633381420
9.479275395	7.287322699	2.681641137
7.886791798	10.008977087	2.721086843
6.301828389	12.750152419	2.725635597
1.579990604	0.961034575	4.252440174
-0.015747499	3.759389903	4.166411420
-3.110886698	9.015926678	4.255984283
-4.730604109	11.839474950	4.297612663
4.732140007	0.940337200	4.256137294
3.175688084	3.759413892	4.166592253
1.579960203	6.372558881	4.162272165
-0.013349233	9.000653157	4.279687297
-1.571693697	11.828556335	4.326280943
7.878793671	0.912615236	4.307405848
6.266435296	3.729812569	4.195374285
3.173256760	9.000635821	4.279847804
1.579952179	11.805476849	4.393517573
11.079611624	0.912556088	4.307597864
9.479237629	3.681932380	4.219734613
7.822871464	6.367766940	4.213549751
6.270798307	9.015906245	4.256515631
4.731622395	11.828525345	4.326615977
14.226309035	0.940284308	4.256678029
12.692020737	3.729772218	4.195757642
11.135516041	6.367765576	4.213739935
9.479192560	9.086890291	4.285297515
7.890538020	11.839428722	4.298326014
1.580156159	0.900698800	1.109297608
-0.022647539	3.640046484	1.025346596
-1.571920974	6.412095995	1.002352762
-3.146992402	9.127823396	1.112448409
-4.726872992	11.838502322	1.153464027
4.751270101	0.893419204	1.116196254
3.182946248	3.640053418	1.025535549
1.580106478	6.452925576	1.005223154

0.057411783	9.191726010	1.130952262
-1.488882619	11.840933292	1.182998929
7.945339669	0.818940601	1.163112882
6.312009438	3.622720483	1.050556160
4.732188255	6.412075681	1.002704030
3.102847370	9.191740147	1.131119614
11.013463424	0.818914715	1.163277210
9.479429047	3.604235933	1.105275290
7.893201931	6.412150612	1.067631401
6.307288784	9.127772176	1.112986299
4.649197628	11.840909403	1.183339460
14.207536824	0.893380147	1.116744945
12.646802876	3.622664055	1.050927420
11.065544899	6.412169635	1.067807193
9.479373334	9.129374191	1.144964514
7.887181045	11.838457600	1.154189402

POSCAR_3vac_top1x3_top3x3_top3x1

```
3vac_top1x3_top3x3_top3x1
1.0
    15.7908132895     0.0000051527    -0.0080735737
    -7.8954072174    13.6752451190     0.0080828287
    -0.0101486661     0.0058790038    14.8789989022
  Mo   S
  25  47
Cartesian
  -0.004864091    1.843855872    2.734347815
  -1.578809499    4.613917482    2.620123475
  -3.111702480    7.268955004    2.621673208
  -4.723600292   10.016850158    2.739150579
  -6.317895926   12.764472367    2.778320326
   3.159751716    1.843913498    2.732713095
   1.577259602    4.606638025    2.648902962
  -0.021805977    7.282993991    2.617158964
  -1.540006493   10.005957565    2.652060509
  -3.141237500   12.757584142    2.739152448
   6.316415571    1.825476387    2.687816730
   4.733329197    4.614042559    2.616864559
   3.176218870    7.283044966    2.615538576
   1.577249519   10.052640536    2.617160356
   0.044458920   12.735588227    2.621676642
   9.472752907    1.797065726    2.636674779
   7.869895364    4.573151022    2.638284846
   6.266198119    7.269050567    2.616866284
   4.694574818   10.005988515    2.648906349
   3.110235272   12.735584253    2.620128321
  12.629166973    1.825385147    2.684590437
  11.075484261    4.573079423    2.636677429
   9.472709903    7.292341346    2.687820845
   7.878410057   10.016873525    2.732718445
   6.296151445   12.757539534    2.734355070
   1.578314992    0.949057948    4.327518828
  -0.027231821    3.763827101    4.238681878
  -3.071274451    9.036261699    4.241778865
```

```
  -4.706042950     11.833867810      4.333927901
   4.758762110      0.925146049      4.295459019
   3.183450562      3.763917344      4.237010122
   1.578010513      6.407125776      4.211510118
   0.020430049      9.105038152      4.213079137
  -1.465859731     11.816920713      4.241780460
   7.941868030      0.853193323      4.220782346
   6.287797915      3.717944528      4.222466417
   3.135681099      9.105091697      4.211510857
  11.005273822      0.853181657      4.219230679
   9.473454982      3.647334188      4.195517483
   7.819474707      6.370888676      4.222468478
   6.227486777      9.036345567      4.237013184
   4.622222207     11.816922347      4.238686457
  14.188519784      0.925091384      4.290669849
  12.659171846      3.717818023      4.219232636
  11.127457755      6.370799140      4.220786087
   9.473590183      9.091472173      4.295464770
   7.862657709     11.833863810      4.327526062
   1.576673868      0.902985457      1.182511461
  -0.013642096      3.633583567      1.099125115
  -1.572092112      6.388730231      1.038600418
  -3.178846235      9.115891378      1.102323250
  -4.748390117     11.858319134      1.188945395
   4.717708498      0.903300823      1.156715169
   3.166660636      3.633685556      1.097468383
   1.576424303      6.421605096      1.052623772
   0.030544733      9.099201118      1.054200326
  -1.588605368     11.870269478      1.102325014
   7.867525370      0.893444984      1.072680858
   6.283896022      3.636199167      1.074279309
   4.724950156      6.388886502      1.035387718
   3.122350679      9.099229476      1.052625434
   1.576562503     11.842361303      1.038603648
  11.076427184      0.893422044      1.071034200
   9.471889734      3.648240663      1.075785261
   7.888319732      6.415144317      1.074281313
   6.331878279      9.116005423      1.097472038
   4.741814090     11.870279106      1.099130474
  14.226398770      0.903224574      1.151915138
  12.659901802      3.636081689      1.071036873
  11.055429934      6.415059494      1.072685225
   9.471984104      9.137951565      1.156720949
   7.901739666     11.858325011      1.182518943
```

POSCAR_3vac_top3x3_bottom2x3_bottom4x2

```
3vac_top3x3_bottom2x3_bottom4x2
   1.0
     0.0000000000   0.0000000000  -15.8037357330
   -13.6865141910   0.0072923528   7.8950579146
     0.0000000000  14.8789986806   -0.0120064293
 Mo  S
 25  47
Direct
```

0.0680211782	0.1340811849	0.2500000884
0.0696401596	0.3346952200	0.2530931647
0.0674577207	0.5334480405	0.2515668400
0.0656893253	0.7329983711	0.2502093614
0.0656895786	0.9323823452	0.2494286156
0.2673269808	0.1348540932	0.2487490767
0.2709620595	0.3384676873	0.2537494846
0.2707149684	0.5317342281	0.2562626507
0.2668132782	0.7318523526	0.2506235708
0.2665721775	0.9332709313	0.2487241399
0.4664464593	0.1349054426	0.2482016604
0.4692958593	0.3393651545	0.2470377501
0.4675565064	0.5321580768	0.2533557785
0.4660038948	0.7301858663	0.2534418709
0.4664922357	0.9328827262	0.2495512382
0.6663774252	0.1337154508	0.2487780604
0.6650517583	0.3359718323	0.2471925874
0.6645177007	0.5346596241	0.2507746510
0.6672115922	0.7290871739	0.2529004982
0.6662396193	0.9313519597	0.2500454103
0.8669255375	0.1330788582	0.2499927244
0.8666227460	0.3342574537	0.2502507008
0.8642679453	0.5346991419	0.2523470169
0.8618271351	0.7296709419	0.2542480687
0.8651876450	0.9302379489	0.2513280572
0.1338321567	0.0666708350	0.3549030785
0.1365304440	0.2668876350	0.3577085077
0.1356528253	0.4681377709	0.3593384867
0.1332947612	0.6671118140	0.3562703578
0.1333620697	0.8669348359	0.3549420670
0.3333531022	0.0673622340	0.3540905043
0.3371788561	0.2700809241	0.3549546051
0.3396535814	0.4671456516	0.3603782073
0.3327463865	0.6655436754	0.3594375895
0.3330547810	0.8666700721	0.3551402940
0.5330051184	0.0678528249	0.3543217943
0.5331070423	0.2717390657	0.3530282194
0.5354318022	0.6627977490	0.3588893037
0.5337245465	0.8669876457	0.3563213906
0.7326223254	0.0663493872	0.3548648415
0.7312061787	0.2677870989	0.3537574708
0.7245706320	0.4653976858	0.3564798993
0.7287800908	0.6614029408	0.3585190208
0.7313963175	0.8633408546	0.3569537506
0.9335694909	0.0660379156	0.3555457868
0.9328977466	0.2665567100	0.3562442081
0.9322538376	0.4668142498	0.3567283003
0.9326319099	0.6676400900	0.3584282697
0.9325731993	0.8641914129	0.3573617982
0.1330269724	0.0666975603	0.1434232090
0.1395526379	0.2726986706	0.1468271463
0.1395604611	0.4672537744	0.1482016769
0.1330399364	0.6656553149	0.1448667745
0.1324353218	0.8658003211	0.1434614997

```
0.3333444893  0.0674085319  0.1426975805
0.3352712691  0.2726862729  0.1443780502
0.3342599869  0.6603491902  0.1473592548
0.3331999183  0.8648321033  0.1437860580
0.5330073833  0.0665414035  0.1431975547
0.5318312645  0.2692482174  0.1415019222
0.5344963670  0.4732040465  0.1445343260
0.5300391317  0.6606736184  0.1479024309
0.5320652127  0.8631491065  0.1456337101
0.7336491943  0.0656786710  0.1440777415
0.7340535521  0.2684537172  0.1433221846
0.7335972190  0.4758155644  0.1436789579
0.7322193980  0.8599417210  0.1456921123
0.9332891106  0.0652710274  0.1451194962
0.9361045957  0.2684060932  0.1460571727
0.9334620833  0.4686800539  0.1460247110
0.9283913970  0.6661778689  0.1463704007
0.9278338552  0.8606396318  0.1458472287
```

POSCAR_3vac_top3x3_bottom2x3_top3x2

```
3vac_top3x3_bottom2x3_top3x2
1.0
   15.7741250992    0.0000000000    0.0000000000
   -7.9565769921   13.7005219751    0.0000000000
   -0.0279159252    0.0028294582   14.8789770679
  Mo  S
  25  47
Cartesian
   0.007400265    1.850918540    1.983800662
  -1.545471259    4.603199416    2.010102673
  -3.166215664    7.315858707    1.983532705
  -4.805431191   10.039893047    1.984955899
  -6.395485112   12.777721368    1.984924102
   3.143367639    1.841543014    1.978581804
   1.619964626    4.663186706    2.018668363
   0.075640122    7.322620435    2.018719174
  -1.620184092   10.044125276    1.979159582
  -3.229801050   12.785494934    1.981738493
   6.286489459    1.837916354    1.949206991
   4.701616781    4.631413015    1.889692960
   3.206188899    7.362249723    1.893088526
   1.575304077   10.014717527    1.889935033
  -0.065605655   12.775778465    1.949644959
   9.452709605    1.829438791    1.933750306
   7.829540356    4.584331861    1.889445136
   6.162743879    7.274628497    1.785220882
   4.595151923    9.973547611    1.785380826
   3.084622308   12.754753186    1.890055510
  12.622429361    1.833359836    1.951290892
  11.026864545    4.580666695    1.950663261
   9.395251116    7.306605389    1.934952200
   7.764129772   10.009480262    1.944102858
   6.224870369   12.765566480    1.935614823
   1.566351172    0.918346937    3.558109971
```

```
  0.053508983     3.694019621     3.572500739
 -1.542607082     6.442712594     3.572473895
 -3.203973610     9.131834936     3.558419632
 -4.813435485    11.866052649     3.560823739
  4.728560364     0.925918146     3.546116599
  3.185816309     3.721238290     3.534284609
  1.767240623     6.526693760     3.530045545
  0.033719002     9.149349249     3.534637412
 -1.629967309    11.874934726     3.546668812
  7.885918110     0.919842555     3.517250698
  6.259421947     3.729795747     3.483551976
  1.564108979    11.814552147     3.484177689
 11.034057513     0.880355013     3.489157192
  9.411990174     3.673549491     3.488509748
  7.710462899     6.408026059     3.435022122
  6.000745105     8.985208650     3.426132391
  4.609561252    11.747030095     3.435564428
 14.181666422     0.893531245     3.528075411
 12.604946688     3.660492709     3.536116051
 10.983166581     6.400772491     3.527412628
  9.349030076     9.109481403     3.530879936
  7.768111800    11.831866769     3.531098201
  1.567776569     0.914456265     0.412503917
  0.051041445     3.745715965     0.442726463
 -1.498784826     6.414735085     0.442603030
 -3.205993075     9.135622771     0.412712795
 -4.810514982    11.867499038     0.414004088
  4.706786880     0.909069176     0.397557541
  3.104419254     3.692249849     0.400315090
 -0.032168638     9.093316572     0.400678638
 -1.655151601    11.863915159     0.398107637
  7.866202402     0.907870645     0.374231264
  6.243637807     3.647106838     0.335165764
  4.557986918     6.369211610     0.257629080
  3.013357675     9.028697634     0.257848503
  1.484623763    11.842013946     0.335769268
 11.047411144     0.928912885     0.358755645
  9.460839707     3.660508989     0.358100172
  7.842392656     6.404346869     0.294139437
  6.242583723     9.125812675     0.257578229
  4.671682872    11.863822829     0.294767847
 14.214844334     0.933033348     0.388432808
 12.653830140     3.688323516     0.408645978
 11.034449282     6.410003383     0.387685779
  9.394085939     9.143620138     0.392603884
  7.819483968    11.854510842     0.392855664
```

POSCAR_3vac_top3x3_bottom2x3_top3x5

```
3vac_top3x3_bottom2x3_top3x5
   1.0
     0.0000000000    0.0000000000  -15.7878541946
   -13.6837630815   -0.0206185524    7.9033679713
     0.0000000000   14.8790033173   -0.0024844990
 Mo  S
```

25 47
Direct

0.0685112402	0.1347774267	0.2500002524
0.0696398393	0.3348401785	0.2531633009
0.0676370785	0.5332545042	0.2511885920
0.0670530572	0.7333154082	0.2487903437
0.0672877654	0.9338362217	0.2483516049
0.2687397599	0.1347524672	0.2491216326
0.2708300352	0.3382360935	0.2538598797
0.2704171836	0.5322281122	0.2562044910
0.2678824365	0.7333229780	0.2502433762
0.2695943713	0.9348745346	0.2463812779
0.4684532285	0.1323031932	0.2425184108
0.4678356350	0.3368974626	0.2459071435
0.4656374454	0.5320122838	0.2495292479
0.4654480815	0.7309736610	0.2511614440
0.4682959914	0.9366528988	0.2432590392
0.6623591781	0.1314783096	0.2420631877
0.6636145711	0.3319455683	0.2432973561
0.6615445018	0.5316681266	0.2420885786
0.6639618278	0.7299988270	0.2448291969
0.6647924184	0.9335962534	0.2452091700
0.8661385775	0.1336590946	0.2485351174
0.8657735586	0.3328159451	0.2488608933
0.8651035428	0.5325096846	0.2476318317
0.8653333187	0.7322140932	0.2466293877
0.8661969900	0.9331824780	0.2471921814
0.1347990334	0.0670082942	0.3550394126
0.1366850883	0.2673373818	0.3581366338
0.1356494874	0.4685947597	0.3595576643
0.1340336502	0.6680777669	0.3558643074
0.1357067972	0.8682081699	0.3532334781
0.3399980962	0.0675340593	0.3520600303
0.3381025195	0.2702398598	0.3551503109
0.3402838111	0.4680124819	0.3595259257
0.3336794078	0.6669575572	0.3590096966
0.3392506242	0.8727518916	0.3525639155
0.5333902240	0.2662798762	0.3496391367
0.5355020165	0.6616819501	0.3551136303
0.5338397026	0.8718541860	0.3520774920
0.7269894481	0.0663648769	0.3507558505
0.7256913185	0.2618282438	0.3500618854
0.7256439328	0.4647148550	0.3495468863
0.7277194858	0.6614419818	0.3500754155
0.7308569551	0.8650612235	0.3506167500
0.9322956801	0.0660132021	0.3541294716
0.9319837689	0.2662473917	0.3557422952
0.9308955669	0.4660927057	0.3551589868
0.9318972230	0.6662080288	0.3533514269
0.9319676161	0.8657100797	0.3529782518
0.1347006559	0.0682216435	0.1436240755
0.1396476627	0.2729913295	0.1468516829
0.1396394223	0.4669685960	0.1480116885
0.1342004091	0.6654807925	0.1444483937

```
    0.1337190717   0.8664925098   0.1424828055
    0.3343580067   0.0686488822   0.1400133981
    0.3334887326   0.2705596089   0.1445825742
    0.3326224387   0.6606411934   0.1470697783
    0.3339329362   0.8644512296   0.1406847068
    0.5322266817   0.0657634139   0.1360487700
    0.5291006565   0.2661994695   0.1383269444
    0.5264344215   0.4666189551   0.1401931986
    0.5237352252   0.6591930390   0.1421073187
    0.5295121670   0.8628080487   0.1411195854
    0.7340744734   0.0681188330   0.1395987597
    0.7342919112   0.2659390271   0.1392097044
    0.7325369120   0.4677121341   0.1387529164
    0.7323133945   0.6649746895   0.1382782493
    0.7322658897   0.8658655286   0.1405234333
    0.9350945353   0.0686437935   0.1431459271
    0.9367272854   0.2685936689   0.1453197432
    0.9349601269   0.4665005505   0.1437433202
    0.9337170720   0.6660428643   0.1422640511
    0.9337671399   0.8667614460   0.1418062033
```

POSCAR_3vac_top3x3_bottom2x3_top4x2

```
3vac_top3x3_bottom2x3_top4x2
   1.0
    0.0000000000   0.0000000000  -15.7661247253
  -13.6942619984   0.0004843201   7.8277284281
    0.0000000000  14.8789935359   0.0172409134
 Mo  S
 25  47
Direct
    0.0677415580   0.1343639940   0.2500004381
    0.0693861172   0.3352167904   0.2536320149
    0.0673579350   0.5339088440   0.2529661726
    0.0658242479   0.7332578897   0.2511056848
    0.0660150200   0.9327659011   0.2493284642
    0.2667191625   0.1339998991   0.2512805621
    0.2701618671   0.3378390074   0.2533150155
    0.2712935507   0.5320555568   0.2571088417
    0.2675313652   0.7318874597   0.2548334076
    0.2669073343   0.9330052733   0.2531215609
    0.4662226438   0.1345369667   0.2531039865
    0.4668216109   0.3382257819   0.2471495181
    0.4679627717   0.5311182141   0.2448986220
    0.4680439532   0.7285764217   0.2548801358
    0.4668440819   0.9321725368   0.2548310598
    0.6662960649   0.1341834217   0.2531414147
    0.6640659570   0.3371046781   0.2493127020
    0.6629213690   0.5379015803   0.2373033223
    0.6684005260   0.7281471491   0.2385283983
    0.6664639115   0.9311411381   0.2507704956
    0.8667379618   0.1337523907   0.2508453746
    0.8661058545   0.3351646066   0.2519702699
    0.8632614017   0.5357264877   0.2477047970
    0.8623228669   0.7322320342   0.2418029376
```

```
0.8655759096   0.9311242700   0.2452100596
0.1320074350   0.0659050122   0.3560275044
0.1364054382   0.2671298087   0.3578930527
0.1361391098   0.4683223665   0.3602903219
0.1320745647   0.6664984822   0.3588225330
0.1315275282   0.8662272692   0.3571405239
0.3327488899   0.0673114583   0.3584394759
0.3359071314   0.2700304687   0.3559451945
0.3440291583   0.4683847726   0.3575746401
0.3352654874   0.6642859578   0.3621527700
0.3332652450   0.8659091592   0.3602973881
0.5337842107   0.0677972734   0.3595826294
0.5329124331   0.2740782500   0.3557145169
0.5466176271   0.6541070342   0.3528534307
0.5357197523   0.8641851544   0.3593860292
0.7338671684   0.0653784350   0.3571575740
0.7318865657   0.2705426812   0.3567769787
0.7246948481   0.4769888520   0.3502837687
0.7331700325   0.8568149805   0.3500433807
0.9323111773   0.0634530410   0.3538092611
0.9331988097   0.2672531307   0.3572928122
0.9305191636   0.4695362151   0.3561707662
0.9260213375   0.6675440073   0.3526931188
0.9273857474   0.8614268303   0.3513094179
0.1342886537   0.0673844069   0.1444150156
0.1384266615   0.2728071511   0.1466482747
0.1396596879   0.4680934251   0.1491064729
0.1352444589   0.6666987538   0.1473636374
0.1349739135   0.8664934039   0.1456350996
0.3339430690   0.0663516000   0.1467969044
0.3328590691   0.2692198455   0.1450878329
0.3347814381   0.6621442437   0.1502955225
0.3341765404   0.8650113941   0.1486758899
0.5323520899   0.0656646043   0.1485066826
0.5304868221   0.2665369511   0.1445858983
0.5281754732   0.4648965001   0.1366941140
0.5262895823   0.6615546942   0.1400600012
0.5307317973   0.8627861739   0.1489600428
0.7320011259   0.0668608100   0.1464519765
0.7320764065   0.2675398290   0.1466319141
0.7300197482   0.4679596424   0.1388648827
0.7345367074   0.6661952734   0.1314961975
0.7309173942   0.8664088249   0.1388631332
0.9342275858   0.0691580326   0.1432643230
0.9350687861   0.2692312598   0.1471779898
0.9340017438   0.4678497315   0.1457638358
0.9346574545   0.6680584550   0.1411861274
0.9350150228   0.8665957451   0.1392836127
```

POSCAR_3vac_top3x3_bottom2x3_top4x4

```
3vac_top3x3_bottom2x3_top4x4
1.0
    15.7905263901    0.0000000000    0.0000000000
    -7.9067103784   13.6778722422    0.0000000000
```

```
    0.0065476392      0.004775122486      14.879001318
  Mo   S
  25   47
Cartesian
    0.012957760       1.847550717       2.697337665
   -1.559383927       4.581037125       2.739044621
   -3.156639726       7.276578718       2.720316960
   -4.743548561      10.018713464       2.701515718
   -6.324727849      12.767697373       2.691633630
    3.167622127       1.848010761       2.699930233
    1.599097521       4.627778396       2.765329889
    0.058648591       7.277976656       2.817020986
   -1.575197690      10.016250500       2.749269016
   -3.156963726      12.771849181       2.709884302
    6.336505504       1.860119247       2.656747842
    4.732865463       4.626324872       2.651692269
    3.152503597       7.274338558       2.732247126
    1.578642969       9.988963647       2.776916150
    0.001415198      12.778971660       2.722061121
    9.470718290       1.890636513       2.610124954
    7.900124740       4.557712866       2.560104599
    6.252465763       7.263367832       2.608087753
    4.722281533       9.981292509       2.670529500
    3.143084861      12.767676249       2.699010486
   12.611079578       1.846612041       2.654026188
   11.000432348       4.540443844       2.608518835
    9.454101918       7.257243052       2.636173967
    7.876148825      10.006759295       2.671173004
    6.302852799      12.762875232       2.678852177
    1.583057650       0.921231307       4.267860984
    0.030264995       3.653814900       4.309004256
   -1.577747980       6.391264696       4.337264661
   -3.186372163       9.100524062       4.297742705
   -4.751362063      11.852488421       4.273083551
    4.767931199       0.945163811       4.257894119
    3.204979684       3.700994463       4.274556427
    1.664926897       6.388222394       4.351509073
   -0.010162470       9.101842857       4.365342078
   -1.583287230      11.874344654       4.303173535
    7.937506930       1.022355868       4.238761129
    6.366117089       3.725278493       4.196532396
    3.238647969       9.033469492       4.312485220
    1.585872950      11.870968114       4.306582200
   11.014362996       0.991749147       4.223444371
    7.847179224       6.314712317       4.175907335
    6.282266328       9.028877288       4.225825798
    4.734931055      11.831391914       4.251511550
   14.191578694       0.925702627       4.240135472
   12.538911422       3.650400243       4.233034700
   10.973443838       6.302430545       4.232786448
    9.448222889       9.072109151       4.238883015
    7.874638307      11.835087734       4.251912080
    1.589581031       0.927937007       1.124372455
    0.048098708       3.742318501       1.164695231
```

-1.482151426 6.398678441 1.191578415
-3.136734774 9.106757860 1.152321426
-4.731032069 11.851269867 1.130531245
4.739332999 0.915895973 1.120779521
3.132509508 3.710554029 1.141997379
0.036772543 9.049584868 1.208668925
-1.566911923 11.832594951 1.157254153
7.912279322 0.898873449 1.091421976
6.268296769 3.677501081 1.046900032
4.625954059 6.400200587 1.092355320
3.061598057 9.023145585 1.144440628
1.560765371 11.807421867 1.171473931
11.032038628 0.902660871 1.083280689
9.471692363 3.655547038 1.009599386
7.848824214 6.395453696 1.021295537
6.298885297 9.094975597 1.069585647
4.709116635 11.838333716 1.115419391
14.224015403 0.929791733 1.109872993
12.660046143 3.695673151 1.100218362
11.088278335 6.374010646 1.078636284
9.476645402 9.118203098 1.102562039
7.889248862 11.851104359 1.108767616

POSCAR_3vac_top3x3_top1x2_top2x4

3vac_top3x3_top1x2_top2x4
1.0
15.7717208862 0.0000000000 0.0000000000
-7.8381429407 13.6860871060 0.0000000000
-0.0008697727 -0.0073385245 14.8790016896
Mo S
25 47
Cartesian
0.036486128 1.839760968 2.699363677
-1.542070088 4.574414573 2.736872418
-3.115433304 7.345877328 2.696915413
-4.645244385 10.003937221 2.693253115
-6.250542976 12.765472116 2.742873342
3.149173936 1.873943039 2.637909265
1.673762638 4.532505253 2.581277969
0.041652620 7.287152929 2.706585047
-1.578327445 9.997485141 2.697126354
-3.118047018 12.745682248 2.722351458
6.254154744 1.860566467 2.708274500
4.674181711 4.601711660 2.544375284
3.243109953 7.239665425 2.580874520
1.608593443 10.009528162 2.736741942
0.022884192 12.763787599 2.764343179
9.441026978 1.838532004 2.768478652
7.838039137 4.592896801 2.707747147
6.283310512 7.280639991 2.637028153
4.766064846 9.998743867 2.698745600
3.188910811 12.768058819 2.762829083
12.621302581 1.832161860 2.762936798
11.051512225 4.581613297 2.763961049

9.506213687	7.316134988	2.721595532
7.932267281	10.024517329	2.742350477
6.357115407	12.768630999	2.752224640
1.628064142	0.981949948	4.271315972
0.146935463	3.673671814	4.242488840
-1.548788243	6.441406364	4.293920981
-4.650703983	11.796231453	4.298582525
4.674461539	0.968782817	4.260854187
1.798605109	6.268771788	4.204915983
-0.014648538	9.087516479	4.293973556
-1.584782381	11.792175988	4.307562592
7.822132347	0.940600354	4.317265245
6.113291143	3.767769368	4.226192511
3.229823591	8.991380971	4.242035220
1.611861083	11.831288391	4.330222420
11.030405856	0.917170947	4.336540397
9.415596769	3.689811515	4.316915493
7.826755703	6.407043537	4.259995676
6.301342837	9.043727747	4.270519974
4.774481630	11.816144581	4.307363192
14.235113745	0.929087355	4.307657484
12.650162727	3.666330431	4.330152213
11.095440388	6.459580606	4.307050306
9.568147215	9.117671252	4.298097809
7.936324703	11.852882220	4.325269790
1.594105624	0.883369346	1.119807401
-0.000328302	3.657787618	1.100444972
-1.567191523	6.386094828	1.148352664
-3.111574752	9.115068482	1.110306873
-4.698402059	11.829842612	1.148538680
4.717749387	0.889876075	1.124423423
3.186100645	3.617229979	0.994518997
1.592190674	6.388062990	1.055416884
0.023822231	9.131073576	1.148362041
-1.523103048	11.830845167	1.156824133
7.874289732	0.911510968	1.188376350
6.286324651	3.666841653	1.078164390
4.788725190	6.381886226	0.993960628
3.169919846	9.127158137	1.100028219
1.607630700	11.862300392	1.192090662
11.026776433	0.919379283	1.199836338
9.466840436	3.658538976	1.188007283
7.916842462	6.408396716	1.123583264
6.370118131	9.122518544	1.119027833
4.765913069	11.871832579	1.173699748
14.182947865	0.909221108	1.173981938
12.621633406	3.654386022	1.192037766
11.093027873	6.386787552	1.156320503
9.515824854	9.142964986	1.148040420
7.931313872	11.856363360	1.180454755

POSCAR_3vac_top3x3_top1x2_top3x5

3vac_top3x3_top1x2_top3x5
1.0

```
    15.7861452103       0.0000000000       0.0000000000
    -7.8932755247      13.6798318952       0.0000000000
    -0.0592593048       0.0132070807      14.8788796554
  Mo   S
  25   47
Cartesian
   0.002687354      1.829290794      2.204497541
  -1.591714715      4.579603623      2.183341189
  -3.170405491      7.345208403      2.095909513
  -4.694277235     10.005458827      2.089164825
  -6.302765281     12.759010909      2.188054751
   3.179344958      1.834405444      2.170929451
   1.598704104      4.582850628      2.147232182
  -0.029013680      7.320428448      2.139426741
  -1.624385249     10.017592336      2.090795355
  -3.127821395     12.754449701      2.106340943
   6.344807531      1.809516428      2.062875016
   4.734955861      4.606813512      2.067364077
   3.186396132      7.278217534      2.084036836
   1.553866931     10.034554689      2.155064640
  -0.012406310     12.804777801      2.080964900
   9.413828165      1.806298490      2.071102442
   7.857019076      4.546886718      2.067911983
   6.263623241      7.268301826      2.061237572
   4.719121692      9.999563191      2.131857703
   3.113955291     12.778630222      2.137084595
  12.603871865      1.828306071      2.181822589
  11.030074408      4.566489777      2.149739488
   9.477178362      7.305883962      2.110358005
   7.891866962     10.018672868      2.158942755
   6.304610260     12.765048691      2.188840184
   1.613194367      0.914488187      3.768535344
   0.017070619      3.685051449      3.751427252
  -1.625124067      6.468474202      3.720777357
  -4.657522102     11.786002604      3.698591612
   4.841967424      0.896864956      3.686914971
   3.216663792      3.732346147      3.707014270
   1.629304699      6.409894482      3.696136249
  -0.088813368      9.132248906      3.710584741
  -1.575225978     11.857814638      3.672592882
   6.309920332      3.651078723      3.641359220
   3.172254952      9.056569332      3.704224085
   1.520055250     11.915804254      3.706222726
  10.934085403      0.915129990      3.704229232
   9.384747272      3.601629682      3.667281079
   7.819900622      6.363722568      3.644559489
   6.268038465      9.029641549      3.692498556
   4.692144827     11.832566337      3.717590597
  14.184881833      0.912064097      3.768005289
  12.596784085      3.664804855      3.749293524
  11.052520628      6.443822629      3.699947947
   9.542195014      9.104940716      3.694689535
   7.889927239     11.832566367      3.756204819
   1.574369180      0.914005042      0.624492767
```

```
  -0.011885036     3.644392694      0.616077467
  -1.605817005     6.380051372      0.566775913
  -3.159457983     9.115753147      0.508614679
  -4.758537400    11.846646929      0.560117463
   4.725722982     0.928722908      0.545062649
   3.159386015     3.640348383      0.559562402
   1.566387364     6.404343288      0.555831314
  -0.017612576     9.141282133      0.559368858
  -1.554175831    11.832903898      0.518114734
   7.885470220     0.901657608      0.491285825
   6.265945189     3.629502005      0.491391643
   4.712849020     6.388386991      0.486828726
   3.123666696     9.112292635      0.550833265
   1.551747479    11.846985426      0.551978286
  11.046794029     0.931339118      0.557300704
   9.486318477     3.635225110      0.524396308
   7.879633213     6.409803419      0.513035795
   6.320243601     9.118308913      0.545763814
   4.732852221    11.862448706      0.592829401
  14.207196023     0.919388448      0.626734001
  12.629333672     3.648014767      0.609813704
  11.067085087     6.381084347      0.545519894
   9.470362801     9.148378886      0.550732634
   7.890923773    11.865679591      0.616181516
```

POSCAR_3vac_top3x3_top2x2_top1x1

```
3vac_top3x3_top2x2_top1x1
1.0
    13.6686528142   -0.0618686213    7.9119824350
     0.0000000000    0.0000000000  -15.7935247421
     0.0000000000   14.8785357937   -0.1179768031
 Mo  S
 25  47
Direct
     0.0669861585    0.1332938224    0.2499998673
     0.0683852658    0.3355716765    0.2496511169
     0.0692893639    0.5362293720    0.2464443751
     0.0689641759    0.7377340794    0.2434145283
     0.0681475699    0.9318518639    0.2448701067
     0.2659152746    0.1320558935    0.2466512333
     0.2687630058    0.3354458809    0.2454249515
     0.2692959308    0.5382276773    0.2422057364
     0.2666298747    0.7333698869    0.2404274714
     0.2622654140    0.9310353398    0.2434145625
     0.4648522138    0.1330398619    0.2483209555
     0.4678900838    0.3376653791    0.2427432851
     0.4669114351    0.5330884457    0.2391203868
     0.4617719352    0.7307037115    0.2422057705
     0.4637699425    0.9307099581    0.2464443978
     0.6654752493    0.1331485212    0.2502381483
     0.6650058031    0.3349934519    0.2469803846
     0.6623342037    0.5321094990    0.2427433078
     0.6645535231    0.7312363983    0.2454249628
     0.6644273997    0.9316138029    0.2496511397
```

0.8664226532	0.1335762888	0.2514796170
0.8668504954	0.3345237673	0.2502381483
0.8669592738	0.5351469516	0.2483209441
0.8679434061	0.7340840101	0.2466512105
0.8667052388	0.9330129028	0.2499998788
0.1347928941	0.0622597858	0.3534064955
0.1374325902	0.2685794831	0.3541537529
0.1416392028	0.4745696485	0.3523450572
0.1388976574	0.6720275283	0.3495062547
0.3282176257	0.0606469400	0.3522594316
0.3380640149	0.2710739970	0.3507954959
0.3391765058	0.4717718363	0.3480660343
0.3279640377	0.8610938788	0.3495062547
0.5305774808	0.0639222488	0.3541162418
0.5321458578	0.2712618411	0.3518683358
0.5282198787	0.6608151794	0.3480660571
0.5254217386	0.8583521843	0.3523450800
0.7315241099	0.0646507666	0.3563551406
0.7320963144	0.2678948641	0.3546288325
0.7287295461	0.4678454995	0.3518683358
0.7289174795	0.6619274616	0.3507954959
0.7314117551	0.8625586033	0.3541537529
0.9341841936	0.0658068583	0.3565651798
0.9353402853	0.2684669196	0.3563551178
0.9360689521	0.4694137573	0.3541162190
0.9393444062	0.6717737317	0.3522594088
0.9377315044	0.8651983738	0.3534064955
0.1307970583	0.0663431138	0.1415028993
0.1315202713	0.2668758929	0.1433924090
0.1333994716	0.4674075842	0.1403930728
0.1315014810	0.6685823798	0.1372663585
0.1319928021	0.8680146337	0.1375235773
0.3324229121	0.0661154390	0.1405647344
0.3331125975	0.2667590976	0.1395643628
0.3313679397	0.4682697952	0.1362048745
0.3319228590	0.6680848002	0.1355999085
0.3314250708	0.8685060144	0.1372664040
0.5324905514	0.0675191432	0.1434474557
0.5309178829	0.2667374313	0.1404681862
0.5325895548	0.4674180746	0.1351704351
0.5317378044	0.6686396599	0.1362048745
0.5325996876	0.8666077852	0.1403930728
0.7326079607	0.0672614351	0.1452911312
0.7327190638	0.2672879100	0.1441295874
0.7332698107	0.4690893591	0.1404681862
0.7332482338	0.6668947339	0.1395643628
0.7331311107	0.8684867620	0.1433924547
0.9323139786	0.0676928908	0.1453098754
0.9327454567	0.2673988938	0.1452911312
0.9324878454	0.4675164819	0.1434474557
0.9338917732	0.6675843001	0.1405647344
0.9336640835	0.8692101240	0.1415028993

POSCAR_3vac_top3x3_top2x2_top2x4

```
3vac_top3x3_top2x2_top2x4
1.0
     15.7657976151       0.0000000000       0.0000000000
     -7.8545560581      13.6917848972       0.0000000000
     -0.02426843276      0.0075578355      14.8789818137
  Mo   S
  25   47
Cartesian
    0.029642185       1.844684771       2.824509266
   -1.531140421       4.582086529       2.867037310
   -3.089244229       7.318200588       2.879632784
   -4.695657320      10.039203953       2.910101553
   -6.279917195      12.784604485       2.884411962
    3.143405496       1.879196730       2.764577562
    1.663506010       4.541809836       2.695916784
    0.070110778       7.323585255       2.783390797
   -1.506556355      10.015931227       2.818180510
   -3.133722141      12.781240879       2.875429375
    6.245125522       1.863920570       2.821004692
    4.657270966       4.610832585       2.660509889
    3.195276789       7.259106764       2.652193107
    1.558720497       9.996030383       2.753143517
    0.008518118      12.751997086       2.835496479
    9.432929922       1.835505300       2.872617265
    7.820208140       4.594635753       2.832783963
    6.250618458       7.286980540       2.763220802
    4.725899421      10.003402843       2.801079825
    3.156462120      12.762841434       2.854956573
   12.611216954       1.834506211       2.881883210
   11.039669519       4.575651416       2.897950060
    9.460742325       7.308085903       2.895666101
    7.887867170      10.028104151       2.887396769
    6.325460648      12.775086243       2.872893915
    1.616647818       0.997798628       4.402575586
    0.151347713       3.678443437       4.368065374
   -1.448644671       6.455973329       4.424606014
   -3.032856596       9.128186249       4.449728890
   -4.700763840      11.881096668       4.472614419
    4.677326738       0.996942005       4.400104176
    1.782132047       6.315879795       4.299316906
   -1.516384046      11.816466558       4.423699607
    7.824041436       0.918487297       4.407087503
    6.094121401       3.766306406       4.341246715
    3.116262368       9.006748455       4.305631913
    1.555137573      11.761482303       4.388504978
   11.010944073       0.900619510       4.440583666
    9.399132281       3.672508210       4.435180767
    7.771944640       6.396367086       4.404594296
    6.234635621       9.048561836       4.398797715
    4.729575885      11.817702467       4.413126866
   14.217930357       0.930056125       4.429141740
   12.647302974       3.667664365       4.458636028
   11.066727846       6.404908556       4.463691856
```

```
   9.467868396      9.123853988      4.479400570
   7.891917883     11.866117149      4.461142984
   1.587792310      0.883295205      1.249702054
  -0.008618498      3.658517766      1.224849289
  -1.534980683      6.393646651      1.278120676
  -3.128388223      9.141089789      1.298068009
  -4.709668862     11.849205339      1.325207667
   4.705638276      0.890191150      1.249202658
   3.173189297      3.615172327      1.115125624
   1.556872406      6.378894849      1.134156071
  -0.001947048      9.110158170      1.214474144
  -1.580675848     11.842577453      1.269821834
   7.862323740      0.922055485      1.286650395
   6.274157669      3.675692167      1.196148066
   4.774769064      6.388748506      1.103099127
   3.151861766      9.150556627      1.155370045
   1.596147347     11.858122183      1.242059880
  11.024534131      0.924480249      1.305621404
   9.455767265      3.671326877      1.309408748
   7.901023362      6.418703386      1.264057193
   6.349700730      9.126378974      1.243630919
   4.748766348     11.877611080      1.281746808
  14.174648811      0.911806084      1.295938807
  12.616993974      3.667067188      1.318879247
  11.058148365      6.402293872      1.331725048
   9.488571732      9.131626243      1.333178553
   7.909897173     11.855537278      1.313697584
```

POSCAR_3vac_top3x3_top2x2_top3x5

```
3vac_top3x3_top2x2_top3x5
1.0
     15.7819585800       0.0000000000       0.0000000000
     -7.9119194057      13.6796765811       0.0000000000
     -0.0157965763      -0.0094063466      14.8789921661
  Mo   S
  25  47
Cartesian
   0.004126056      1.828328160      2.881679768
  -1.573999537      4.575329342      2.858786841
  -3.136897293      7.313117411      2.826570338
  -4.731743293     10.032237786      2.859084988
  -6.325299433     12.770110524      2.881889932
   3.184547195      1.841213320      2.859697219
   1.601090312      4.588706955      2.813961499
   0.002919423      7.348360095      2.757163287
  -1.541522521     10.017481111      2.757302234
  -3.136665829     12.778104701      2.814399091
   6.350791285      1.800641897      2.743180247
   4.728112798      4.606552608      2.743011087
   3.147934400      7.293274811      2.698410517
   1.512042523     10.003364173      2.719238160
  -0.021100672     12.772017646      2.698796481
   9.409976745      1.788756882      2.735999480
   7.849729115      4.539948456      2.750201814
```

6.241964167	7.264386319	2.735580647
4.689914993	9.996324899	2.777539725
3.091236733	12.761351600	2.777726814
12.599478140	1.820046217	2.851375326
11.028469436	4.550571997	2.835881961
9.443510069	7.289151838	2.835837472
7.859524105	10.012584030	2.851383294
6.280887727	12.760563391	2.857286809
1.607744357	0.929057171	4.454827472
0.028666509	3.683202186	4.425566877
-1.523598393	6.465752284	4.393144194
-3.067608461	9.133697097	4.393328793
-4.705751459	11.866039453	4.425994177
4.854357105	0.913494530	4.389916368
3.213304964	3.751579972	4.389686616
1.621731478	6.463304913	4.335799323
-1.501447261	11.861620675	4.336147273
6.302486201	3.644775874	4.318932755
3.077419184	9.065058239	4.310792140
1.479613772	11.828551167	4.311071363
10.922249372	0.884204915	4.364792981
9.379683883	3.575757288	4.340495309
7.775756423	6.346890976	4.340277508
6.211236009	9.026100013	4.364549616
4.656413656	11.821624366	4.370738661
14.175731210	0.904943024	4.439385570
12.605384064	3.654722898	4.425511099
11.037479685	6.383479787	4.405733777
9.452559507	9.102298722	4.425603398
7.852756251	11.835068815	4.439451308
1.583338338	0.914747511	1.310201008
-0.006060856	3.638199730	1.290843599
-1.571485426	6.375763857	1.242053280
-3.169578997	9.137253097	1.242191398
-4.761883472	11.858407996	1.291204829
4.725950073	0.926156764	1.234987058
3.160419602	3.633689062	1.234783866
1.538779967	6.385230112	1.176619257
-0.032754268	9.109305297	1.166255071
-1.610506141	11.829181435	1.176981399
7.877634575	0.901901519	1.147700883
6.264575836	3.622910454	1.172550194
4.709327531	6.378360359	1.147336997
3.114159580	9.127476268	1.150145242
1.551889979	11.829095555	1.150317972
11.046344424	0.929590836	1.216398175
9.483054185	3.633184593	1.205373699
7.877443001	6.407622971	1.205179472
6.312813006	9.111421442	1.216153399
4.724264859	11.861016323	1.246140189
14.208453889	0.917250285	1.298772390
12.630329980	3.647642361	1.285992979
11.041604227	6.385853087	1.272012430
9.459173164	9.127654473	1.286141721

```
   7.879956378      11.857370061       1.298889176
```

POSCAR_3vac_top3x3_top2x3_top1x3

```
3vac_top3x3_top2x3_top1x3
1.0
     15.8500490189       0.0000000000       0.0000000000
     -7.9253876969      13.5993892571       0.0000000000
     -0.1305276419       0.1941711277      14.8771639098
   Mo   S
   25   47
Direct
   0.069851458      0.139058560      0.250000387
   0.070894994      0.339961112      0.235470384
   0.067325331      0.530270815      0.241855249
   0.066538252      0.732451737      0.252990574
   0.067655534      0.935288250      0.255502164
   0.269168466      0.139078587      0.249992788
   0.271109611      0.342548877      0.231789231
   0.262993127      0.526375234      0.234142959
   0.264095128      0.727407217      0.249177665
   0.266496807      0.933270693      0.254104018
   0.466420591      0.134421617      0.248986199
   0.468804896      0.339957029      0.235459536
   0.463078141      0.526377916      0.234128579
   0.462242872      0.724564314      0.248116791
   0.465181231      0.930257976      0.252494246
   0.666146994      0.132360548      0.249055147
   0.665826976      0.334428847      0.243559927
   0.662797928      0.530282795      0.241852194
   0.663248897      0.727413833      0.249201983
   0.665042877      0.930250943      0.252500713
   0.867943645      0.134433538      0.248991638
   0.868479252      0.334431946      0.243572325
   0.866579771      0.533226430      0.247929484
   0.865893602      0.732438505      0.253005296
   0.866773427      0.933274865      0.254107952
   0.137710124      0.073690504      0.358110189
   0.147656620      0.289983124      0.345089912
   0.132171646      0.655968726      0.354117393
   0.133929208      0.864849806      0.360582173
   0.335392922      0.070235707      0.357040286
   0.343901426      0.290005624      0.345075399
   0.323494703      0.644620836      0.349592447
   0.331586957      0.859901309      0.357601613
   0.533533514      0.066218458      0.355846763
   0.532822013      0.274608821      0.347839206
   0.522694349      0.644596517      0.349589080
   0.529440522      0.857210994      0.356660634
   0.734381139      0.066231251      0.355839014
   0.734936297      0.268251956      0.350472331
   0.728398621      0.463725686      0.350472659
   0.725448310      0.655940711      0.354143381
   0.730024576      0.859927177      0.357628763
   0.936546266      0.070241570      0.357046306
```

```
   0.943393946     0.274631798     0.347855508
   0.936951101     0.463728547     0.350498468
   0.932001412     0.662268817     0.357643872
   0.932667255     0.864849329     0.360585839
   0.133528218     0.068809792     0.147675335
   0.131364211     0.266316324     0.133350328
   0.133055851     0.471875370     0.129875794
   0.131927550     0.669317186     0.142569885
   0.133106008     0.869541287     0.149127960
   0.331302106     0.067200229     0.146502033
   0.333005428     0.266324729     0.133331344
   0.335789233     0.473663539     0.125960201
   0.331870049     0.666481435     0.138145119
   0.331714243     0.867041707     0.147299156
   0.531080008     0.064900234     0.145161644
   0.531964481     0.265688300     0.136938572
   0.536825299     0.471873581     0.129859447
   0.532743752     0.666495144     0.138151824
   0.531610370     0.864995480     0.146606758
   0.732037008     0.064892627     0.145169601
   0.731956720     0.265731543     0.140290111
   0.735262573     0.470935613     0.138381660
   0.735578060     0.669348776     0.142573088
   0.733559608     0.867037833     0.147320643
   0.934124649     0.067182064     0.146504149
   0.931850493     0.265705645     0.136951432
   0.933817327     0.470923543     0.138397574
   0.934704661     0.671207666     0.146136656
   0.934686899     0.869526327     0.149129361
```

POSCAR_3vac_top3x3_top2x3_top3x2

```
3vac_top3x3_top2x3_top3x2
1.0
    15.7933530807     0.0000000000     0.0000000000
    -7.8968531160    13.6773386265     0.0000000000
    -0.0493530752     0.02944758006    14.87889253288
  Mo   S
  25   47
Cartesian
   0.006175167     1.846925911     2.154424194
  -1.551440106     4.584886375     2.077356242
  -3.144647734     7.301410408     2.154402649
  -4.746588547    10.031113846     2.231213978
  -6.327704686    12.771536414     2.231224192
   3.150337346     1.885589310     2.186429739
   1.588461498     4.662699118     1.998879571
   0.084505238     7.267519065     1.998457769
  -1.539154266    10.006907451     2.186886970
  -3.163870542    12.772501632     2.231214776
   6.294816142     1.848590581     2.153890997
   4.708654466     4.663362377     1.998295624
   3.147715946     7.300981562     1.903532266
   1.645057481     9.970511012     1.998459365
   0.001094228    12.750072559     2.154404884
```

9.461690926	1.826229924	2.120608557
7.849749725	4.586303202	2.076508170
6.212433315	7.268019299	1.998296741
4.652876368	9.970496360	1.998882444
3.150277443	12.728618038	2.077359115
12.632597231	1.824967082	2.120791449
11.046014269	4.570400095	2.120607440
9.443177617	7.301791738	2.153890199
7.838861370	10.006469583	2.186430697
6.300229655	12.748705454	2.154425471
1.568248182	0.972360112	3.769198768
0.060210615	3.765270653	3.644820394
-1.455345550	6.390549573	3.644754004
-3.120804683	9.091178161	3.769359317
-4.745631862	11.858139652	3.813770270
4.733943092	0.973315900	3.768853091
3.148727638	3.936721270	3.641011890
0.236747972	8.981604642	3.640997846
-1.536957664	11.834520061	3.769360594
7.899287473	0.924880797	3.716140799
6.238248291	3.767436634	3.643949660
1.634587062	11.742546705	3.644756558
11.046924709	0.880563503	3.671494927
9.434087594	3.671599554	3.670759527
7.753136792	6.391335496	3.643949980
6.061738711	8.982273980	3.641013805
4.665925526	11.742716405	3.644823267
14.195293968	0.922759232	3.716849707
12.657615113	3.670402614	3.671493650
11.045382624	6.374155079	3.716139522
9.420729060	9.091184496	3.768852772
7.838674428	11.833213739	3.769199725
1.596892314	0.901386379	0.633085971
0.016903322	3.631643679	0.504772497
-1.593407583	6.419825615	0.504663296
-3.167552682	9.151906589	0.633535302
-4.745455782	11.858038413	0.667111352
4.704614086	0.902486878	0.632795633
3.148824415	3.635983762	0.493747440
1.603786643	6.408492511	0.375456625
-0.024652775	9.132523867	0.493614699
-1.612924031	11.844641275	0.633536499
7.862770144	0.903299964	0.577905388
6.281114351	3.633523749	0.504226852
4.692231720	6.409269751	0.375413974
3.148648693	9.084310300	0.375458620
1.540200907	11.847472693	0.504666169
11.047156316	0.918101093	0.540763533
9.466777186	3.652725259	0.540641446
7.890538888	6.421171109	0.504227051
6.322229777	9.132563264	0.493749275
4.759992830	11.847035130	0.504775569
14.232239534	0.901427373	0.578161972
12.625221070	3.651431315	0.540761858

```
  11.045810740      6.416570233      0.577903792
   9.467401888      9.151999691      0.632795154
   7.914460029     11.843894446      0.633086450
```

POSCAR_3vac_top3x3_top2x3_top4x2

```
3vac_top3x3_top2x3_top4x2
1.0
     15.8041410446      0.0000000000      0.0000000000
     -7.8400661195     13.6442250000      0.0000000000
     -0.02061301375     0.0249585782     14.87896831316
  Mo   S
  25  47
Cartesian
   0.010565271      1.842180578      2.712276052
  -1.548855117      4.570139847      2.663957344
  -3.135457088      7.286960720      2.742057289
  -4.708918966     10.019125916      2.783645312
  -6.267208332     12.748813840      2.769428783
   3.160999488      1.891672831      2.747875782
   1.607792301      4.645818836      2.548046609
   0.083843889      7.235498099      2.627889549
  -1.515818664      9.986948000      2.794011358
  -3.104981825     12.750984661      2.814655678
   6.310177242      1.850357650      2.759640118
   4.726293687      4.655611866      2.547178144
   3.234215932      7.198831239      2.450291935
   1.675081949      9.912588026      2.709878437
   0.065686574     12.716152692      2.799579302
   9.475244560      1.821675082      2.757740729
   7.858733567      4.599989260      2.677990350
   6.288076684      7.317320988      2.506725573
   4.848735323      9.908296422      2.569744354
   3.229062631     12.685730119      2.741297342
  12.643022693      1.813981009      2.724754054
  11.055886091      4.564743370      2.725957330
   9.450424511      7.313765656      2.697402030
   7.896949442      9.992398811      2.648772290
   6.376011118     12.696011057      2.694099883
   1.571608380      0.968954587      4.322900700
   0.084951109      3.752133934      4.206575522
  -1.471829409      6.357240451      4.251106304
  -3.124604890      9.077199688      4.354657289
  -4.709431894     11.838636783      4.372644827
   4.723903174      0.984590849      4.354130656
   3.161305386      3.948939482      4.201531338
   0.146592224      8.964655363      4.291733621
  -1.508933131     11.810371010      4.384198829
   7.897457103      0.935595916      4.349142009
   6.234684291      3.787063494      4.230620931
   3.389423358      8.804640721      4.157801037
   1.681039586     11.733576482      4.322572911
  11.077794565      0.886728962      4.312272295
   9.439706556      3.692095154      4.288800117
   7.718314849      6.490929879      4.202494916
```

```
  4.844251128     11.658808033     4.236370483
 14.230881125      0.894397369     4.279839702
 12.675920981      3.662970408     4.270193387
 11.037455910      6.389298208     4.292737096
  9.409573127      9.097649891     4.289198762
  7.899875398     11.763983853     4.282100711
  1.607813600      0.918149616     1.183342025
  0.008654589      3.628357368     1.067512121
 -1.573605241      6.412252123     1.104841201
 -3.115998200      9.130674619     1.209551576
 -4.667610067     11.845549492     1.223247217
  4.735527134      0.909877543     1.216245317
  3.173758614      3.624769420     1.039724826
  1.580304693      6.402808656     0.948787412
  0.027208659      9.103705918     1.147852329
 -1.530071665     11.825564549     1.240268464
  7.891445853      0.888539720     1.209193067
  6.328942501      3.616931147     1.094219339
  4.806858013      6.381649904     0.895102717
  3.206180571      9.084062809     0.999222784
  1.620417097     11.795516634     1.199288463
 11.043203508      0.887948993     1.178288426
  9.476269499      3.633904628     1.160609048
  7.910907169      6.399802368     1.047161279
  6.413394479      9.113415683     0.984139865
  4.779623467     11.808357656     1.100181779
 14.223288435      0.908743239     1.147515284
 12.626230212      3.636865691     1.140965809
 11.074286282      6.393607291     1.160565003
  9.540713919      9.143431088     1.140034627
  7.995619194     11.834213512     1.131154961
```

POSCAR_3vac_top3x3_top2x4_top4x2

```
3vac_top3x3_top2x4_top4x2
1.0
    15.7392301559      0.0000000000      0.0000000000
    -7.7574601467     13.6947124086      0.0000000000
     0.0188061069     -0.01091077802    14.878987639496
  Mo   S
  25  47
Cartesian
  0.041555988      1.835017250     2.677076713
 -1.513334747      4.565708681     2.757842683
 -3.082538768      7.303560057     2.834648625
 -4.648005022     10.041983310     2.817840860
 -6.188656921     12.780366267     2.742443473
  3.147763132      1.876380860     2.651167728
  1.699746467      4.532589501     2.555118421
  0.134533766      7.266845930     2.772318135
 -1.463425334     10.026332355     2.855447081
 -3.046639924     12.789522568     2.817840860
  6.248681256      1.869788500     2.760495182
  4.679050187      4.611055027     2.534407274
  3.317550893      7.239805933     2.498877574
```

1.725199116	9.996027311	2.772317489
0.107641389	12.777108676	2.834648303
9.427666437	1.845122384	2.811022731
7.807869515	4.622687743	2.744133480
6.275879831	7.350811981	2.534407435
4.875729553	9.981784343	2.555116968
3.263267903	12.761159606	2.757842683
12.599059324	1.830212875	2.759480725
11.022988454	4.582295848	2.811022731
9.434690815	7.336175383	2.760495827
7.900589212	10.031039381	2.651166114
6.405609580	12.754134438	2.677076713
1.602517684	0.979941925	4.267513359
0.187243520	3.658526169	4.227330167
-1.452050868	6.361876364	4.365307221
-3.076905954	9.120599114	4.418457252
-4.654007063	11.885917819	4.374630021
4.652021076	1.020118304	4.321706573
1.937201495	6.244913557	4.185122258
0.180159216	9.068393467	4.381129902
-1.470588418	11.876634911	4.418456284
7.825987284	0.957069017	4.374159104
6.090458799	3.796509298	4.253091487
3.502872797	8.931209826	4.185119676
1.730626692	11.822551205	4.365306575
11.038430915	0.896827383	4.347861668
9.383943787	3.704096839	4.360303365
7.680269580	6.524209148	4.253096006
4.890779086	11.728611725	4.227330490
14.212631730	0.889765600	4.267213831
12.642005115	3.648158514	4.347861991
11.006259440	6.413615579	4.374159750
9.387031032	9.144211244	4.321705604
7.918968978	11.817384922	4.267513682
1.610683185	0.882184753	1.112371532
0.002922871	3.666014320	1.090341398
-1.517300494	6.414358012	1.233379034
-3.045531533	9.128910034	1.273160462
-4.584926036	11.845655129	1.228115351
4.732102025	0.877507065	1.174792155
3.208002054	3.598857883	0.984022616
1.600141412	6.390790212	1.035916115
0.087103682	9.122629139	1.250501255
-1.462356164	11.845240083	1.273160623
7.861905420	0.904580575	1.246134460
6.306386186	3.654795532	1.107586996
4.840665135	6.352078669	0.924060508
3.209817499	9.152586823	1.035917406
1.652802744	11.853458877	1.233378711
10.987360836	0.926378628	1.213976303
9.441532186	3.670532674	1.235743140
7.909997816	6.406179825	1.107584898
6.431551625	9.129664063	0.984021163
4.793416839	11.885298652	1.090341478

```
  14.147485992    0.927735143    1.141233271
  12.591121299    3.678030144    1.213976626
  11.069633142    6.408233991    1.246134945
   9.550588464    9.144821856    1.174793447
   8.008052517   11.858463180    1.112371532
```

POSCAR_3vac_top3x3_top2x5_top4x2

```
3vac_top3x3_top2x5_top4x2
1.0
    15.7603483200    0.0000000000    0.0000000000
    -7.8252159386   13.6925959888    0.0000000000
     0.0016068012    0.0058861458   14.87900227376
  Mo  S
  25  47
Cartesian
   0.029035787    1.826527469    2.799908961
  -1.555253915    4.571853499    2.845196023
  -3.133630636    7.314123829    2.882071142
  -4.698104301   10.056763050    2.853945423
  -6.222226975   12.794862775    2.776023952
   3.210346935    1.807972294    2.719510655
   1.636078889    4.568915243    2.754058644
   0.063584507    7.302497072    2.844950871
  -1.529334976   10.051001279    2.883319402
  -3.098586177   12.832036123    2.779384958
   6.252991141    1.813668665    2.753302343
   4.734754775    4.573241102    2.663021509
   3.273553344    7.265547061    2.663445480
   1.669410646   10.014690427    2.842401085
   0.028966421   12.792166237    2.837906621
   9.432760782    1.835854919    2.852422566
   7.828629617    4.598081976    2.790804569
   6.258964523    7.338682589    2.630938941
   4.841759179    9.983492122    2.663844474
   3.211500309   12.763345127    2.825882895
  12.609303381    1.826837165    2.838514374
  11.020942717    4.580965452    2.864096924
   9.411450922    7.335145101    2.815784659
   7.868706014   10.030591175    2.722358197
   6.363541552   12.755599188    2.760036099
   1.672962916    0.924124625    4.344111593
   0.071386013    3.657113357    4.369591582
  -1.520107367    6.388425185    4.432885615
  -3.132524170    9.142856606    4.454698572
  -4.659431189   11.948397903    4.382084108
   3.246550004    3.647259902    4.291801455
   1.765163317    6.377556296    4.325825393
   0.092129983    9.120080713    4.436612530
  -1.566805102   11.946176440    4.413223120
   7.778671624    0.936010748    4.392670003
   6.209776312    3.675061989    4.313966756
   3.436523565    8.986047697    4.305600469
   1.640670656   11.853991446    4.408878195
  11.030690200    0.896529224    4.414263943
```

```
   9.397072170     3.688401712     4.408554634
   7.677932485     6.496662428     4.322613265
   4.842729672    11.748301801     4.315963377
  14.214337978     0.877251260     4.368441912
  12.619092508     3.651524893     4.421345909
  10.986251774     6.418272431     4.424897809
   9.370289313     9.151655376     4.376149166
   7.911741740    11.827362920     4.323659051
   1.594286780     0.928746184     1.189071390
  -0.005152179     3.661334688     1.237473395
  -1.559217297     6.410602480     1.293587699
  -3.104405186     9.131250092     1.310226977
  -4.636364623    11.848881980     1.232802758
   4.748078965     0.890353259     1.167773303
   3.165202462     3.615033767     1.127242234
   1.589654280     6.407891564     1.181931941
   0.043338566     9.139217462     1.300390187
  -1.535788725    11.849844698     1.262913684
   7.878807708     0.921396767     1.244118155
   6.332343117     3.617212974     1.160523602
   4.779469873     6.361144823     1.064992995
   3.181261444     9.132303983     1.152009181
   1.616780767    11.851167736     1.281660744
  11.016166170     0.922406348     1.275096544
   9.458933348     3.662825915     1.279074897
   7.877594052     6.409695127     1.170301832
   6.381155936     9.120599354     1.078961813
   4.756092215    11.871176315     1.178594425
  14.177939257     0.934747397     1.234499969
  12.602334815     3.670103214     1.284651871
  11.045791842     6.408498365     1.296428541
   9.507190594     9.146768243     1.229822136
   7.958985372    11.860295092     1.181676284
```

POSCAR_3vac_top3x3_top2x5_top5x2

```
3vac_top3x3_top2x5_top5x2
1.0
    15.7793474197     0.0000000000     0.0000000000
    -7.8642232128    13.6799780063     0.0000000000
     0.0098666699    -0.0056653542    14.8789991748
  Mo   S
  25  47
Cartesian
   0.031224372     1.815681294     2.721030558
  -1.566727783     4.564773241     2.754446696
  -3.176346328     7.318346543     2.726375026
  -4.752748885    10.016617940     2.649313247
  -6.250466116    12.746279042     2.646041647
   3.214899474     1.803159979     2.608547749
   1.628538260     4.562576756     2.674750572
   0.029940470     7.297603694     2.750290772
  -1.579587606    10.043746636     2.754646644
  -3.137530939    12.808253977     2.649313566
   6.259839583     1.818753882     2.632176468
```

4.749822797	4.577838666	2.564876797
3.231471586	7.260107307	2.632121068
1.603362171	10.017001049	2.750290772
-0.012593448	12.786368915	2.726375344
9.445502354	1.830028730	2.744499605
7.863378263	4.580123236	2.656291207
6.313603005	7.280573113	2.564876638
4.771226949	9.994191547	2.674750253
3.176842695	12.763247621	2.754446696
12.628894330	1.822834377	2.765277315
11.036096073	4.579105066	2.744499605
9.458177103	7.346549250	2.632176150
7.954137377	9.994146631	2.608547430
6.356578940	12.748007655	2.721030558
1.701287620	0.889195627	4.229554267
0.062510645	3.650574808	4.286749881
-1.576988978	6.401220040	4.316508055
-3.239825179	9.134710215	4.292889365
-4.713173530	11.856813276	4.228011675
3.262855324	3.636633782	4.190100847
1.710184085	6.361474016	4.262518294
0.013730086	9.121865940	4.334230828
-1.618934811	11.936148873	4.292889684
7.773602750	0.942121545	4.279454966
6.236189858	3.682871066	4.199281577
3.252355438	9.026861144	4.262518294
1.579612923	11.856882319	4.316508055
11.027688161	0.911480867	4.332042226
9.414875480	3.682439683	4.289331375
7.830285351	6.438000451	4.199281259
6.388499142	9.038790970	4.190100529
4.781402408	11.806396533	4.286749881
14.245461244	0.887501571	4.312485536
12.620976029	3.665214202	4.332042226
10.972617743	6.471087814	4.279454647
7.992137629	11.761886589	4.229553948
1.583557837	0.927735739	1.088338707
-0.006331559	3.649764896	1.157594557
-1.564888317	6.390565325	1.183565341
-3.132492699	9.135049930	1.139299481
-4.664885931	11.828875732	1.073175857
4.750312732	0.893505978	1.049484574
3.159967886	3.626509618	1.036445613
1.586782198	6.408572846	1.116016054
0.009267656	9.124447938	1.189308591
-1.565736279	11.842928266	1.139299719
7.890633374	0.922972564	1.129486829
6.326853818	3.620454915	1.036504992
4.743075981	6.385504894	1.009347336
3.150020806	9.110372041	1.116016133
1.594880867	11.851702134	1.183565501
11.047018424	0.915025014	1.190095169
9.490051151	3.638944030	1.156057139
7.929583089	6.390507165	1.036504754

```
  6.345998368    9.133035940    1.036445374
  4.747794955   11.866483632    1.157594557
 14.196051275    0.916090203    1.171668838
 12.627537557    3.646689915    1.190095010
 11.047545764    6.379172083    1.129486590
  9.507996335    9.116372553    1.049484176
  7.900050876   11.844745606    1.088338548
```

POSCAR_3vac_top3x3_top3x1_top5x1

```
3vac_top3x3_top3x1_top5x1
1.0
    15.7917614189     0.0000208083    -0.0122454549
    -7.8958693548    13.6759686150    -0.0051309175
    -0.0157085880    -0.0178703204    14.8789845011
  Mo   S
  25  47
Cartesian
  0.001197088    1.783497786    2.529381594
 -1.575613908    4.552351562    2.577816408
 -3.161000037    7.298094319    2.576410746
 -4.770492535   10.048039216    2.525708397
 -6.360696476   12.737490051    2.476192903
  3.157068186    1.820398014    2.551709776
  1.610162375    4.570512615    2.525778327
  0.024492252    7.306782937    2.551217982
 -1.552429981   10.048018147    2.522817147
 -3.160642286   12.762734188    2.547046700
  6.314527530    1.820712445    2.548867368
  4.735443375    4.603488894    2.474306372
  3.198960167    7.282122381    2.448901086
  1.590493775   10.068129089    2.447309813
  0.039001335   12.737789316    2.470438409
  9.469998505    1.784335234    2.521501722
  7.860864767    4.571315385    2.520727429
  6.269770499    7.282329257    2.446524001
  4.733819856   10.025309897    2.421124559
  3.126364668   12.726966323    2.444035473
 12.631543552    1.788349938    2.546306105
 11.046796251    4.552859967    2.567953282
  9.444948699    7.307432867    2.544152347
  7.877499339   10.068151431    2.442906041
  6.342805678   12.726540094    2.441920013
  1.543471496    0.842997670    4.100102930
  0.033711033    3.624755922    4.109212870
 -1.555096420    6.395586347    4.145522795
 -3.160108346    9.156691695    4.106424898
 -4.814165729   11.854718560    4.095569320
  4.737260452    0.910599756    4.132838472
  3.198327275    3.709228590    4.096514782
  1.685109341    6.397023035    4.086356232
  0.066268710    9.201065017    4.084743085
 -1.504707625   11.854810180    4.092571743
  7.930495745    0.843241332    4.094538122
  6.275761163    3.709395847    4.093865759
```

```
   3.186247403     9.132956339     4.010919979
  11.014024402     0.807682583     4.083453252
   9.440012624     3.625494744     4.101660927
   7.786729991     6.397702821     4.081586012
   6.283428778     9.132809245     4.008713227
   4.735183390    11.814526027     4.007523343
  14.251007392     0.806932754     4.081341175
  12.632766117     3.615486028     4.137029931
  11.027487427     6.396037340     4.135829980
   9.404395843     9.201663689     4.077926709
   1.594037706     0.895939700     0.946884150
  -0.006472511     3.643905453     0.985309416
  -1.581047271     6.379030834     1.004434004
  -3.162847068     9.110203995     0.982557703
  -4.742465472    11.870601779     0.942318530
   4.734477926     0.908381867     0.988144441
   3.174302482     3.632709406     0.943333070
   1.576509453     6.393810771     0.938137741
   0.010524306     9.106089198     0.936584283
  -1.582080473    11.870740351     0.939391935
   7.874845344     0.896626965     0.941321844
   6.294679798     3.633334907     0.940709331
   4.733283608     6.367008813     0.877026574
   3.118641228     9.092172887     0.865736370
   1.565953717    11.853401166     0.874232909
  11.064862252     0.897674785     0.935126689
   9.475508749     3.645054907     0.977653255
   7.890601898     6.394861353     0.933297172
   6.346680942     9.092488783     0.863354713
   4.733214703    11.887509612     0.862144150
  14.195644607     0.897081546     0.932961060
  12.630409243     3.640074227     0.995957311
  11.048382005     6.379680555     0.994731880
   9.455333792     9.106684590     0.929660311
   7.901031804    11.853396260     0.870059003
```

POSCAR_3vac_top3x3_top3x2_top3x1

```
3vac_top3x3_top3x2_top3x1
1.0
    15.7379055023     0.0000000000     0.0000000000
    -7.9780091685    13.6918211725     0.0000000000
     0.00032096399   -0.0195401988    14.878990690521400
  Mo   S
  25  47
Cartesian
  -0.014139950     1.820229071     2.551955021
  -1.576054301     4.581887985     2.529280705
  -3.170552380     7.318362446     2.529234162
  -4.803087495    10.038919738     2.551892867
  -6.421423641    12.761620425     2.572438629
   3.139394805     1.831715657     2.535928280
   1.602450630     4.606249766     2.481364973
   0.043384930     7.358334058     2.467062063
  -1.582210049    10.071686454     2.481325079
```

-3.238208285	12.776856760	2.535912380
6.281333430	1.822861316	2.465788631
4.702620061	4.612752824	2.375415291
3.172123404	7.347007037	2.260451126
1.576189240	10.085886865	2.260463124
-0.047963705	12.765548312	2.375435960
9.426305656	1.796299758	2.402772685
7.808991860	4.571955010	2.402715446
6.152486819	7.269462913	2.281187397
4.535819748	9.975946953	2.225920162
2.978292491	12.716935566	2.281244781
12.568493596	1.804340357	2.479198838
10.990695315	4.566887665	2.480548589
9.365295443	7.301682807	2.479102283
7.719169004	10.003062245	2.493994932
6.128988704	12.732126854	2.494030490
1.563403631	0.919022722	4.135765842
0.024333870	3.692861743	4.093148223
-1.534189120	6.439083487	4.080491668
-3.154942545	9.149043514	4.093092718
-4.809276667	11.855697652	4.135743293
4.744503468	0.927359669	4.094352852
3.197203928	3.754391168	4.043232292
1.760198231	6.531231214	3.977129177
0.170281194	9.259748272	3.977127153
-1.537035716	11.879102370	4.043229979
7.911751841	0.859703617	3.993098967
6.247378284	3.716182121	3.993081622
10.943106924	0.772877933	3.953011156
9.378640315	3.627566076	3.992647412
7.666532461	6.396174188	3.952933392
5.918538633	8.982628107	3.911280170
4.353429732	11.668637948	3.911303875
14.102760027	0.867796895	4.085883734
12.552274132	3.635161941	4.068733309
10.950061703	6.384861644	4.068690524
9.306990214	9.098233948	4.085815799
7.675243680	11.805263629	4.101639889
1.555581589	0.888646761	0.988926291
-0.009512486	3.643039100	0.963341873
-1.588047271	6.407678818	0.954173596
-3.214978858	9.144210189	0.963303352
-4.839580099	11.863954057	0.988898250
4.679715611	0.889638918	0.947300316
3.119809153	3.616308798	0.894901193
1.546133397	6.392923830	0.830487797
-0.055565913	9.141739658	0.830477245
-1.695291455	11.879920406	0.894877705
7.824590367	0.900753278	0.836961196
6.240059391	3.620121024	0.836925711
4.643602295	6.336501708	0.709691141
3.012428111	9.088398590	0.651045552
1.422616215	11.864331304	0.709719833
11.036780301	0.926804654	0.814391169

```
  9.440101979    3.663332385    0.865286457
  7.846603300    6.401779305    0.814319041
  6.251054289    9.126793845    0.760126201
  4.642746590   11.886950888    0.760171443
 14.177340456    0.895989763    0.952516039
 12.601341084    3.662936726    0.932909379
 10.998460188    6.413827136    0.932848887
  9.368251336    9.149306271    0.952451067
  7.764583273   11.857370725    0.970167526
```

POSCAR_3vac_top3x3_top3x2_top4x2

```
3vac_top3x3_top3x2_top4x2
1.0
    15.7960453033    0.0000000000    0.0000000000
    -7.8980505887   13.6823030163    0.0000000000
    -0.0762583312    0.01818605126   14.8787969885
  Mo   S
  25  47
Cartesian
  -0.005061320    1.825946908    2.196086691
  -1.573875072    4.575298104    2.186160472
  -3.170011440    7.308556329    2.198470189
  -4.765694677   10.035916113    2.210341003
  -6.339397921   12.762317508    2.208750708
   3.154309539    1.844288450    2.206839987
   1.626001768    4.611390489    2.130727195
   0.057617101    7.328889172    2.133146837
  -1.574326629   10.035954501    2.210388492
  -3.169985311   12.774963893    2.234088467
   6.301879478    1.844316152    2.206818755
   4.728018811    4.632420026    2.077270930
   3.235932297    7.352204137    1.926821869
   1.626917633   10.005139920    2.081091237
  -0.000586646   12.762409714    2.208792199
   9.461222209    1.826013762    2.196070807
   7.830146597    4.611395264    2.130630436
   6.220048907    7.352183661    1.926750393
   4.727968781    9.937800208    1.928406329
   3.159295383   12.702866965    2.132586052
  12.626105904    1.809913427    2.185594500
  11.030019945    4.575300131    2.186150261
   9.398454422    7.328845827    2.133047322
   7.829022621   10.005094143    2.081002905
   6.296760535   12.702831859    2.132554771
   1.561310575    0.922160101    3.782451591
   0.041404185    3.693810852    3.737695760
  -1.529329175    6.413854832    3.739807942
  -3.170031197    9.116913209    3.785144656
  -4.768550105   11.852405314    3.797573986
   4.728107640    0.939564789    3.795527283
   3.196140420    3.759334635    3.716443952
   1.806271928    6.526032195    3.628840254
   0.105291099    9.114041710    3.719738645
  -1.571425068   11.852462440    3.797606078
```

```
   7.894860653      0.922270831      3.782469420
   6.260026127      3.759373704      3.716381390
   1.636957671     11.769264855      3.719052413
  11.055564141      0.852752339      3.738463679
   9.414698729      3.693860986      3.737678255
   7.649819069      6.526025133      3.628733932
   4.728032826     11.589982024      3.629449014
  14.196618295      0.852700805      3.738462058
  12.626098896      3.652951821      3.750525095
  10.985356518      6.413860105      3.739785251
   9.350689586      9.113999183      3.719641399
   7.819075927     11.769168561      3.719002169
   1.578342670      0.910149972      0.636895593
  -0.000383457      3.652253417      0.612337301
  -1.586240962      6.400663308      0.614269457
  -3.169998966      9.137809843      0.640149281
  -4.742219683     11.865917351      0.651258456
   4.728073143      0.907016790      0.649220789
   3.169355315      3.631596155      0.568034595
   1.593811135      6.405570876      0.483261727
  -0.019165664      9.155928111      0.571818151
  -1.597768779     11.865944300      0.651287752
   7.877818326      0.910196441      0.636882100
   6.286704986      3.631602631      0.567994967
   4.727950026      6.347465197      0.395467467
   3.110892994      9.148912874      0.398294042
   1.539301598     11.854718098      0.570854930
  11.039966827      0.907477852      0.612926693
   9.456564431      3.652292493      0.612279926
   7.862250175      6.405571960      0.483147868
   6.345003728      9.148901370      0.398222850
   4.728009926     11.833269782      0.484191439
  14.212241035      0.907447151      0.612912632
  12.626100108      3.653591024      0.624127361
  11.042325233      6.400627356      0.614212811
   9.475170002      9.155905179      0.571739099
   7.916690660     11.854691929      0.570796502
```

POSCAR_3vac_top3x3_top4x2_top5x1

```
3vac_top3x3_top4x2_top5x1
1.0
    15.7386636734     0.0000000000     0.0000000000
    -7.7587617911    13.6933535042     0.0000000000
     0.0751805403     0.00752912429    14.878811682713400
  Mo   S
  25  47
Cartesian
   0.029262087      1.801715317      2.496485144
  -1.536170688      4.572466101      2.580352563
  -3.112178122      7.324225891      2.634493242
  -4.699778830     10.077826650      2.583907587
  -6.233562551     12.772225538      2.472597575
   3.174901877      1.827123331      2.562808237
   1.646246200      4.576442565      2.496477311
```

0.090742373	7.306776736	2.579884044
-1.477829609	10.044290061	2.659197030
-3.042713588	12.782457066	2.641562916
6.316812795	1.836029447	2.641601181
4.752706330	4.617285953	2.472566692
3.303920887	7.273977253	2.376312880
1.738293696	10.007838657	2.595360858
0.141695686	12.766590632	2.680767612
9.470575520	1.824378314	2.659230775
7.853071891	4.611096007	2.583897343
6.283192719	7.352167745	2.356428667
4.921358353	9.981194324	2.321374362
3.329022572	12.737517254	2.595354832
12.625564213	1.809090972	2.579899711
11.031474233	4.587199156	2.634493845
9.412178576	7.364131385	2.567651916
7.880075256	10.092376800	2.356438008
6.479439511	12.723070103	2.376359280
1.542713520	0.865975625	4.087557518
0.078444769	3.631577675	4.087314370
-1.492524255	6.388863061	4.170500251
-3.129188544	9.155191207	4.198472528
-4.748282385	11.886341211	4.144884432
4.708028538	0.933629923	4.197655707
3.206490810	3.720999698	4.087542755
1.792024344	6.398486318	4.048721311
0.152524772	9.101101785	4.189066884
-1.472193692	11.861173396	4.243554057
7.892593172	0.923741380	4.243578763
6.255647706	3.761756871	4.144870873
3.541197124	8.985654836	4.007439461
1.785878488	11.808456012	4.205169798
11.094876897	0.870785784	4.189089783
9.430040212	3.699445233	4.198481567
7.693071205	6.538717752	4.075777339
5.107124340	11.672777423	4.007442173
14.254544686	0.776713134	4.048757165
12.643672696	3.639084634	4.170527669
10.987564833	6.446042260	4.184132816
9.282875110	9.266767241	4.075774024
1.632680834	0.905223529	0.932275892
0.011672555	3.670455488	0.960558281
-1.545433822	6.421296214	1.035592599
-3.064824649	9.150218232	1.069510631
-4.582318460	11.885591616	0.997409908
4.780482719	0.891454405	1.050541010
3.216709288	3.623415710	0.932256759
1.606640985	6.409158041	0.910920588
0.086056693	9.156306696	1.056024422
-1.439414312	11.869176626	1.097751440
7.901806220	0.891328036	1.097794752
6.338143930	3.617671710	0.997420755
4.812942864	6.340004610	0.805457805
3.203476655	9.133374350	0.857779543

```
   1.690163529      11.864241234      1.073744255
  11.014088671       0.901468125      1.056021183
   9.466128408       3.645876456      1.069520348
   7.911809691       6.395584130      0.930479699
   6.444937394       9.093323193      0.746445276
   4.812165694      11.893799560      0.857801839
  14.153861272       0.932687543      0.910943260
  12.589396051       3.669125115      1.035584162
  11.045608768       6.412242840      1.058539062
   9.515211961       9.147020259      0.930501016
   8.035926447      11.870576228      0.805497200
```

POSCAR_3vac_top3x3_top4x4_top5x2

```
3vac_top3x3_top4x4_top5x2
1.0
    15.7889099121     0.0000000000     0.0000000000
    -7.8955398159    13.6726336943     0.0000000000
    -0.0603450261     0.0532606978    14.87878582637
  Mo   S
  25  47
Cartesian
  -0.007978073       1.830028570      2.153642948
  -1.609449007       4.570831852      2.151577914
  -3.213443879       7.303686585      2.124153634
  -4.795258838       9.998180266      2.087048858
  -6.322520560      12.732747416      2.126841397
   3.166344460       1.846428753      2.151673213
   1.600524212       4.583800373      2.127951835
  -0.004726820       7.301619328      2.177959667
  -1.605019845      10.032739163      2.198154648
  -3.166210938      12.764193617      2.177962230
   6.335415821       1.869158310      2.123762346
   4.752391852       4.627565638      2.054950103
   3.205306941       7.273606618      2.086341560
   1.580224670      10.019196515      2.196853611
  -0.000581715      12.784629570      2.197370790
   9.459872155       1.891719305      2.086213267
   7.901281653       4.574496040      1.998633291
   6.307974441       7.281531735      1.997697756
   4.747907603       9.990636325      2.121902006
   3.145298910      12.770400936      2.175864522
  12.591731001       1.846579163      2.126132337
  10.977907733       4.554302356      2.054872742
   9.449313715       7.307626991      1.998286690
   7.928091188       9.981939975      2.053377423
   6.312983106      12.746072255      2.149482128
   1.579065498       0.902500841      3.712919483
   0.001022290       3.667873164      3.713260959
  -1.628033597       6.392927575      3.724993515
  -3.281545884       9.097351604      3.717868965
  -4.791637165      11.782900261      3.705094683
   4.753925576       0.951703020      3.724824699
   3.222102382       3.735825132      3.685488316
   1.658194292       6.385135489      3.705323401
```

```
   -0.015185584      9.109862766      3.772511230
   -1.594451793     11.870108496      3.772530450
    7.923076194      1.031359374      3.717040261
    6.365720952      3.762268493      3.639667410
    3.228990742      9.035661101      3.716438353
    1.585552924     11.857176039      3.771399030
   11.003519806      0.995446026      3.703886383
    7.885770851      6.388285704      3.578519480
    6.372069974      9.017893388      3.638089124
    4.744062290     11.819260388      3.722454867
   14.183826498      0.918867716      3.711889609
   12.515218300      3.674037126      3.684822341
   10.920061207      6.383820651      3.639667090
    7.920615095     11.752692155      3.683848526
    1.579493187      0.919648223      0.582057095
   -0.012724055      3.660086908      0.582380272
   -1.584184339      6.400398994      0.591076861
   -3.162563573      9.145989710      0.565219735
   -4.718530305     11.841228603      0.559628945
    4.737987144      0.909943424      0.591067171
    3.170463846      3.654051037      0.540055532
    1.570719252      6.418064739      0.559778661
   -0.014013143      9.134286248      0.627163775
   -1.574687930     11.855192746      0.627453558
    7.905409507      0.904264673      0.564356836
    6.289080552      3.659809747      0.478999578
    4.717883122      6.395998220      0.469837543
    3.126628260      9.113149472      0.563598485
    1.561726900     11.846474010      0.625983144
   11.017583863      0.904310843      0.558159372
    9.463040097      3.640654289      0.470348356
    7.886556052      6.387616395      0.408016191
    6.322056499      9.134520057      0.477338446
    4.715796470     11.853509023      0.588969984
   14.199050639      0.911917567      0.580800064
   12.612011548      3.671559893      0.539261504
   11.047550417      6.368775983      0.479065687
    9.478244012      9.126853481      0.469964195
    7.876685216     11.838446340      0.537996745
```

POSCAR_4vac_top1x3_bottom2x3_top3x3_bottom4x3

```
4vac_top1x3_bottom2x3_top3x3_bottom4x3
1.0
     15.7725076675       0.0000000000       0.0000000000
     -7.8862911497      13.6403815124       0.0000000000
     -0.0160402711       0.0133623473      14.8789888786
   Mo   S
   25   46
Cartesian
    0.001456802      1.846103718      2.072461415
   -1.568970109      4.626634733      2.024256824
   -3.113036825      7.259604908      2.003274670
   -4.733991826      9.975885246      2.073486885
   -6.310466455     12.727023679      2.083312459
```

3.152295453	1.851420267	2.073981219
1.575878655	4.633752878	2.080007199
0.011701916	7.253880732	2.060719783
-1.572366822	9.950246847	2.032404094
-3.157193735	12.727116120	2.064029664
6.303087726	1.846321793	2.075380691
4.727860761	4.633938836	2.067986049
3.151958098	7.268923808	2.073832302
1.577050846	9.936134328	2.103082913
-0.001039399	12.718236576	2.070779687
9.453828620	1.843595914	2.075162622
7.872872095	4.626816653	2.123619173
6.292328652	7.253979449	2.086447236
4.727212795	9.936151241	2.044058944
3.152348069	12.716035879	2.073690746
12.623121503	1.843509094	2.072867084
11.038258616	4.587287248	2.073959309
9.417068471	7.259651472	2.143844592
7.876801264	9.950305947	2.114507884
6.305759101	12.718094586	2.076534023
1.575895167	0.933114987	3.650483307
-0.009736732	3.722507788	3.620227938
-3.110816079	9.021513729	3.611378879
-4.721661463	11.806102181	3.648737732
4.728279300	0.917786678	3.640017734
3.154664184	3.721339440	3.634687210
1.576999293	6.397787003	3.640631201
-0.069836055	8.989750181	3.654736154
-1.581228355	11.780434645	3.623193600
7.884730281	0.914973225	3.643227482
6.288235779	3.700972707	3.651068702
3.212456721	8.999127914	3.664966200
1.574128823	11.796243866	3.653807395
11.039336392	0.920247703	3.642940261
9.452006709	3.671666369	3.659377897
7.814704817	6.390228978	3.698463154
6.231838717	8.999554595	3.668409079
4.726688073	11.775905355	3.631453156
14.196288692	0.919368622	3.643853958
12.659868921	3.727329232	3.627665886
11.104227574	6.396586180	3.646540616
9.478476875	9.070055472	3.695329062
7.877559636	11.809640073	3.662761210
1.576524609	0.917916419	0.507714910
0.015670076	3.700702248	0.496828221
-1.510764947	6.389727811	0.448925870
-3.174091433	9.069901786	0.451578166
-4.745902319	11.806298283	0.498438915
4.728840047	0.933510882	0.497232050
3.149157949	3.721322133	0.513371552
0.072502133	8.999366405	0.478579582
-1.572729946	11.809847534	0.484371447
7.880890186	0.919833213	0.503838633
6.313605294	3.722854559	0.527704545

```
  4.726597380    6.397625695    0.506988130
  3.091726731    8.998884452    0.482394153
  1.578020432   11.776000831    0.515767353
 11.037701439    0.920545960    0.504884386
  9.416614100    3.727337532    0.520379140
  6.374032630    8.989704456    0.492270650
  4.730544824   11.796310541    0.493378281
 14.192288399    0.915079421    0.504511923
 12.624422862    3.671453017    0.488605595
 10.972186373    6.396183106    0.500798684
  9.415251335    9.021534442    0.535567803
  7.886072794   11.780625352    0.523921982
```

POSCAR_4vac_top1x3_top2x3_top3x3_top4x3

```
4vac_top1x3_top2x3_top3x3_top4x3
1.0
    15.8253650665    0.0000000000    0.0000000000
    -7.9127505285   13.4968077054    0.0000000000
    -0.2002041592    0.3110564240   14.8744044616
  Mo   S
  25  46
Cartesian
 -0.032202914    1.927831757    2.749415537
 -1.608601214    4.638951449    2.495709310
 -3.169384042    7.210041765    2.604906299
 -4.782522815    9.923619727    2.811174768
 -6.365032975   12.665664934    2.860575202
  3.128983478    1.945886294    2.749211852
  1.545690632    4.671358434    2.422369016
 -0.040183154    7.149814691    2.491179982
 -1.613029710    9.875472181    2.770403739
 -3.199701383   12.666089071    2.860814604
  6.290718116    1.928316090    2.751485204
  4.710402153    4.671779583    2.424946769
  3.127710061    7.141624947    2.474781924
  1.544915392    9.829018240    2.753877292
 -0.036448539   12.627913328    2.842603978
  9.452853988    1.867218767    2.742019179
  7.866057466    4.639695733    2.500082576
  6.297573165    7.149412448    2.495565154
  4.713520433    9.828746879    2.755442413
  3.130163998   12.605736395    2.834600428
 12.632205291    1.866664664    2.740527423
 11.041880620    4.585627967    2.585677249
  9.428883815    7.210004597    2.608014500
  7.872719315    9.874774188    2.773418142
  6.296847008   12.627315519    2.842369081
  1.549410017    1.085270482    4.369742321
 -0.002638582    4.017304529    4.130139402
 -3.154652453    8.898824513    4.313876750
 -4.782128424   11.733230585    4.433376866
  4.708670934    1.085547351    4.370642974
  3.126833740    4.048439179    4.101343290
 -0.032513470    8.739599008    4.248195706
```

```
  -1.608168656    11.686083492    4.404593303
   7.868671074     1.027230417    4.357711415
   6.258196119     4.017399987    4.133069178
   3.128347661     8.726779650    4.237689377
   1.548629193    11.641701802    4.388294352
  11.043170325     1.001567328    4.350632329
   9.397924048     3.816340429    4.170818081
   6.290539085     8.740304980    4.251294737
   4.711735734    11.641967078    4.388823353
  14.216706595     1.027216639    4.356663066
  12.686907712     3.815896671    4.168524779
  11.043913058     6.332008314    4.182327066
   9.415970270     8.899672732    4.316584179
   7.869059721    11.686687237    4.405304430
   1.550182300     0.945792015    1.240229353
  -0.059986311     3.600209570    0.980324582
  -1.649398320     6.411683934    0.926510981
  -3.221595319     9.095604776    1.161484982
  -4.781991336    11.778185574    1.283889132
   4.709434626     0.946635903    1.241277460
   3.128988210     3.598505592    0.955757017
   1.534371519     6.438739745    0.856833713
  -0.045306210     9.057704368    1.100621980
  -1.628894844    11.758719887    1.270085076
   7.859617120     0.916203417    1.226466967
   6.317244920     3.601631006    0.984168284
   4.723453400     6.438573259    0.859855436
   3.129614797     9.055654333    1.095224901
   1.540226746    11.723421623    1.259601030
  11.042270706     0.892652920    1.213225457
   9.458096173     3.593806126    1.035939086
   7.906702665     6.411827074    0.931316957
   6.304436713     9.056041704    1.104477507
   4.719910816    11.722244457    1.260052000
  14.225249865     0.914631023    1.224694863
  12.623540857     3.592876204    1.033383215
  11.041034312     6.416681956    1.016809225
   9.480481701     9.094601474    1.164094913
   7.889923708    11.756409427    1.271238403
```

POSCAR_4vac_top2x3_top3x3_top3x2_top4x2

```
4vac_top2x3_top3x3_top3x2_top4x2
1.0
    15.7668514252     0.0000000000     0.0000000000
    -7.8244437068    13.6199043247     0.0000000000
    -0.0219741373    -0.0122765240    14.8789822339
  Mo   S
  25  46
Cartesian
   0.007658629     1.826925943    1.888503481
  -1.541097346     4.556510335    1.819911055
  -3.118926840     7.269613177    1.891267107
  -4.692063264     9.992190348    1.949211246
  -6.256884470    12.716356403    1.947912846
```

3.151656277	1.880713173	1.934530556
1.600066280	4.643295532	1.731465135
0.117038032	7.225336439	1.736591406
-1.488644086	9.957817357	1.940552900
-3.097561435	12.719534157	1.984898399
6.292447315	1.846917788	1.931524845
4.712165395	4.659921052	1.747127480
3.203982033	7.279954242	1.599746340
1.699228434	9.904992032	1.756642248
0.064755933	12.687624668	1.936312976
9.450456931	1.813231222	1.907568329
7.830934771	4.595971663	1.839495382
6.243732791	7.322172004	1.647451400
4.770963363	9.887292740	1.652615526
3.214062293	12.632040659	1.846060398
12.612024922	1.798021516	1.880815270
11.029871375	4.552270121	1.880090063
9.421172433	7.297612573	1.851808190
7.875705814	9.965924507	1.813121310
6.348879370	12.645537919	1.853793345
1.560963046	0.955277036	3.505857971
0.071789986	3.739093366	3.381657120
-1.435823835	6.359198235	3.384523535
-3.091409325	9.049863804	3.509591283
-4.695009332	11.802538754	3.545962647
4.719930407	0.970665937	3.532748231
3.145961297	3.934632572	3.387735871
0.281452202	8.916509795	3.395065743
-1.486426538	11.772632082	3.536633257
7.888294296	0.917584387	3.503183967
6.230140120	3.777888757	3.412371062
1.700965624	11.662600372	3.420355670
11.046707519	0.846043402	3.446196497
9.416797662	3.680869379	3.443384843
7.671025820	6.496589302	3.350072690
4.777518630	11.537284037	3.355089440
14.181769306	0.867745720	3.444708080
12.645813992	3.649176334	3.424690453
11.014946131	6.376208105	3.444512078
9.391450408	9.069534842	3.453411760
7.866432409	11.727141720	3.453744514
1.606514699	0.909513680	0.363429852
0.022330364	3.611111788	0.240307273
-1.569156055	6.385607159	0.243479549
-3.104081226	9.113404349	0.366751897
-4.661378567	11.820668396	0.394147610
4.717147726	0.906713959	0.391585820
3.168133598	3.621404248	0.235962129
1.626625627	6.377963046	0.097015803
0.025405688	9.096067854	0.244130320
-1.539278669	11.799096810	0.395352572
7.859217454	0.896541203	0.361294404
6.290949489	3.619418579	0.271853831
4.741520445	6.371691656	0.077041655

```
   3.191319559       9.070722333       0.083772023
   1.595815076      11.791213764       0.279707016
  11.017521125       0.891179928       0.318720142
   9.445623389       3.629929798       0.316239633
   7.879469996       6.386801494       0.196545685
   6.391447665       9.115110735       0.123773509
   4.783485983      11.771515088       0.202119955
  14.195934466       0.890784540       0.314214455
  12.598739091       3.621801923       0.297099415
  11.046017211       6.378664515       0.314378364
   9.518358837       9.126978054       0.301512112
   7.976869668      11.808091696       0.301837872
```

POSCAR_4vac_top2x4_top3x3_top4x2_top5x1

```
4vac_top2x4_top3x3_top4x2_top5x1
1.0
    15.6452579498     0.0000000000     0.0000000000
    -7.6427282466    13.6514903654     0.0000000000
     0.0962310678     0.0107260207    14.8786884645
  Mo   S
  25  46
Cartesian
   0.067087087       1.805284315       2.443220902
  -1.474581118       4.551428896       2.597592627
  -3.038292841       7.295513956       2.697412669
  -4.615725212      10.047095090       2.627834247
  -6.127448890      12.737906689       2.463798100
   3.147146211       1.870042341       2.462865523
   1.719048975       4.516811331       2.355136940
   0.176400270       7.248047761       2.606738082
  -1.405686306      10.005237673       2.715493462
  -2.970876321      12.757235914       2.666919392
   6.233273368       1.864409905       2.627578375
   4.677536185       4.600107695       2.350497837
   3.341706648       7.222378034       2.282547962
   1.790689274       9.966395451       2.606271191
   0.195648220      12.735384162       2.715389006
   9.405346092       1.832184193       2.697501773
   7.790709299       4.612234840       2.611060205
   6.273093037       7.337361141       2.333697154
   4.942138238       9.952143986       2.282820842
   3.374619232      12.702139772       2.607910877
  12.562860541       1.808467715       2.598182336
  10.987573304       4.575268834       2.689550486
   9.392663719       7.345895121       2.611329623
   7.882309717      10.067193368       2.351408288
   6.510281597      12.690246847       2.356208290
   1.574105129       0.922696388       4.039561766
   0.234116459       3.613167023       4.027300698
  -1.399215511       6.343601693       4.213056886
  -3.054869827       9.127220102       4.259982239
  -4.677024465      11.870921831       4.171756494
   4.611778626       1.027382134       4.171576782
   1.993916176       6.217809733       3.989538259
```

```
   0.248177210     9.039623418     4.225740293
  -1.409378123    11.841926913     4.288280740
   7.798612833     0.951799118     4.259534012
   6.054009729     3.802134329     4.106167261
   3.603981171     8.902749062     3.960588038
   1.846250899    11.764634485     4.226474494
  11.036520323     0.867310172     4.213925043
   9.357136628     3.698028238     4.243493871
   7.641819300     6.535706523     4.095425770
   5.161038814    11.618917538     3.990394676
  14.216531729     0.776123264     4.027434053
  12.619115700     3.618311140     4.206704045
  10.955680509     6.426751517     4.243867444
   9.251605995     9.256689368     4.106828614
   1.646491770     0.879239566     0.873980081
   0.012005173     3.671947167     0.891481852
  -1.490002017     6.406503420     1.081509591
  -2.983648940     9.109646212     1.125852898
  -4.476386330    11.829103664     1.019128988
   4.746586827     0.872300381     1.018571865
   3.227200974     3.586036078     0.785046098
   1.606824356     6.378332384     0.839690873
   0.130185763     9.109272973     1.096847440
  -1.371378707    11.819713705     1.138840717
   7.848967564     0.898250854     1.125795628
   6.319486900     3.631226164     0.958951741
   4.859905231     6.318245959     0.717035868
   3.217127752     9.129420434     0.814150519
   1.728889381    11.834043731     1.097206413
  10.937353914     0.914694229     1.082309114
   9.421622024     3.650387485     1.115404229
   7.923534896     6.370656431     0.952580538
   6.472272096     9.068548579     0.717719497
   4.831592973    11.878375693     0.840387070
  14.056044978     0.941013978     0.892061928
  12.526888503     3.672086102     1.069532390
  11.028551750     6.393440237     1.115138951
   9.529641619     9.108710847     0.959330732
   8.059153622    11.828818347     0.787013224
```

POSCAR_4vac_top3x3_bottom2x3_top3x2_bottom4x2

```
4vac_top3x3_bottom2x3_top3x2_bottom4x2
1.0
    15.7298959862    -0.0561792837    -0.0114784088
    -7.9135946841    13.7067337982     0.0010296834
    -0.0144860762    -0.0069716207    14.8789948397
  Mo   S
  25  46
Cartesian
   0.014352769     1.838800887     2.914993407
  -1.526329443     4.601745060     2.941074157
  -3.148786515     7.317414405     2.915522390
  -4.794804360    10.032406946     2.915115132
  -6.370982929    12.762471384     2.915261489
```

3.138247807	1.823050347	2.890259414
1.637878293	4.649214748	2.947075837
0.096862557	7.318310600	2.947314869
-1.600500879	10.030691326	2.890919721
-3.207738094	12.768800214	2.885593309
6.275333667	1.803927230	2.849240407
4.724331623	4.595830153	2.811176217
3.230632486	7.348150370	2.794055246
1.593826405	10.017937425	2.811598370
-0.048532206	12.757112218	2.850065420
9.432540976	1.791041536	2.838461716
7.817067722	4.545627574	2.807202160
6.204681816	7.228211771	2.790734227
4.613782834	9.983798278	2.790889592
3.096788280	12.721402287	2.807761071
12.594311549	1.779631579	2.874133813
11.003540608	4.534815831	2.874384049
9.354199424	7.265813484	2.899533864
7.708899089	9.933610874	2.946542479
6.221206673	12.692515787	2.899848051
1.574636518	0.903646409	4.481828638
0.071557170	3.686258023	4.502388507
-1.520119186	6.443070855	4.502656612
-3.178401049	9.136091812	4.482544561
-4.780518627	11.860616286	4.479182601
4.720408211	0.898565646	4.450106918
3.203140763	3.699318118	4.455181018
1.794808887	6.519090108	4.442996721
0.056973445	9.148633631	4.455627993
-1.609937750	11.863016704	4.450941920
7.865226185	0.886044572	4.419678217
6.261789387	3.675677515	4.402618696
1.565822559	11.809341772	4.403198047
10.998044667	0.840442164	4.405655166
9.392172283	3.621827737	4.405919669
7.684712400	6.331056451	4.415619223
5.994652361	8.943885540	4.392495510
4.576994237	11.713968630	4.415938248
14.147610028	0.843270559	4.461301929
12.581600112	3.608947548	4.457692998
10.969353302	6.347964132	4.461802226
9.324452056	9.074052762	4.493719093
7.772389436	11.762407894	4.493857434
1.551546201	0.888731109	1.333121519
0.062611494	3.732816826	1.373331584
-1.484503277	6.412482181	1.373586084
-3.203074532	9.123825938	1.333804458
-4.802872644	11.847880017	1.332371311
4.685858045	0.885993441	1.298808872
3.107596785	3.690967733	1.313126774
0.001711444	9.070438845	1.313551534
-1.638326311	11.839665798	1.299647935
7.849598454	0.877276200	1.269961946
6.243596214	3.617550574	1.241153112

```
  4.638888700     6.375123830     1.216241079
  3.091936109     9.054465657     1.216470582
  1.506131901    11.822960577     1.241763707
 11.032620167     0.883966145     1.268808396
  9.446934617     3.630427996     1.269024112
  7.804236121     6.437222009     1.240789737
  4.728452197    11.764706696     1.241131568
 14.179003514     0.845746448     1.325986639
 12.628354473     3.636259443     1.331214631
 10.986990394     6.374360373     1.326498904
  9.287793267     9.083766829     1.350551731
  7.762278399    11.726055589     1.350705219
```

POSCAR_4vac_top3x3_bottom2x3_top3x2_top4x2

```
4vac_top3x3_bottom2x3_top3x2_top4x2
1.0
    15.7485189438     0.0000000000     0.0000000000
    -7.8880766799    13.6476550482     0.0000000000
     0.0146930155    -0.0042425894    14.8789956653
  Mo   S
  25  46
Cartesian
  0.011349853     1.834384818     2.589946671
 -1.536038579     4.577515178     2.621942933
 -3.140431030     7.287579276     2.586834707
 -4.754899896    10.004329577     2.570483129
 -6.328543254    12.726554111     2.572573420
  3.144158006     1.837159460     2.595228373
  1.631392967     4.648949253     2.621333842
  0.106993605     7.286667259     2.618651311
 -1.574293396    10.000896482     2.591431181
 -3.165717400    12.738884946     2.599863668
  6.284778491     1.838226697     2.590330664
  4.708680759     4.625167078     2.502072134
  3.254907891     7.347420828     2.404552344
  1.622355130     9.965525797     2.498942866
 -0.005924019    12.721852651     2.588431165
  9.442654406     1.820395974     2.579657118
  7.811214359     4.596997657     2.516297008
  6.181429006     7.323889681     2.302146882
  4.695431442     9.895071866     2.301555997
  3.146148211    12.668328878     2.515331008
 12.603329318     1.808602042     2.575130762
 11.009939138     4.565272297     2.574332081
  9.370744949     7.305340053     2.499754186
  7.797497849     9.973043924     2.438696526
  6.271403243    12.668028677     2.501182571
  1.560373970     0.900515889     4.165333508
  0.065706976     3.676514625     4.179955616
 -1.516991204     6.416428321     4.177808749
 -3.175429113     9.095569534     4.162242309
 -4.762754618    11.814738879     4.160849584
  4.717092177     0.923407063     4.174952580
  3.185955445     3.712076893     4.146873883
```

```
   1.845616611       6.533739144       4.096458924
   0.071000024       9.101483197       4.143035163
  -1.579724715      11.818518838       4.172983264
   7.878461299       0.914968943       4.163402111
   6.253019043       3.742535605       4.117666984
   1.627934710      11.743063813       4.115104827
  11.033550475       0.847775206       4.123331775
   9.394781989       3.684053242       4.123450945
   7.623509628       6.513713419       4.006160509
   4.712063158      11.548568265       4.005461439
  14.167152219       0.839865360       4.122193942
  12.590936836       3.640431824       4.155689460
  10.952089705       6.404503032       4.120444309
   9.316469303       9.089209704       4.082965321
   7.789701188      11.729069673       4.084161234
   1.576102928       0.922176528       1.014581802
   0.054950442       3.727721563       1.049611108
  -1.477875849       6.378807951       1.047204083
  -3.150351663       9.098985788       1.010858339
  -4.731456741      11.834861028       1.010191995
   4.718173875       0.916023790       1.022086884
   3.116159548       3.681819364       1.011353903
   0.010498317       9.055593357       1.007446193
  -1.587400722      11.826024919       1.019687758
   7.862431575       0.907537271       1.018015164
   6.254409799       3.634229317       0.967077933
   4.617600297       6.324601532       0.810589947
   3.050199838       9.037992012       0.809265007
   1.536031565      11.800395391       0.964533531
  11.021982507       0.908804921       0.995305065
   9.440326945       3.643898634       0.995319735
   7.825280926       6.376052269       0.856319807
   6.306498405       9.111128720       0.764249265
   4.695858053      11.793161306       0.855961018
  14.186151918       0.924272551       0.993786925
  12.629276185       3.661937902       1.031337762
  11.033033985       6.377228601       0.991607783
   9.441990286       9.122309234       0.928770171
   7.881253938      11.823561257       0.930127461
```

POSCAR_S8

```
S8
1.0
    20.0000000000    0.0000000000    0.0000000000
     0.0000000000   20.0000000000    0.0000000000
     0.0000000000    0.0000000000   20.0000000000
 S
 8
Direct
  0.5540716575911774  0.6216260561236701  0.5040910567390756
  0.4375836645629171  0.6221648970693689  0.3972706318529057
  0.6330651651237726  0.4167843545442039  0.5176113295664361
  0.3584342546116520  0.4170486829106210  0.3807866472190197
  0.6464037540040247  0.5316888892179601  0.4089894703930394
```

```
0.3449205903929306  0.5316317526622077  0.4907119866770283
0.5603311003920749  0.3291127747946049  0.4040617507088818
0.4309530549214592  0.3290629124773601  0.4937254642436098

0.00000000E+00  0.00000000E+00  0.00000000E+00
0.00000000E+00  0.00000000E+00  0.00000000E+00
0.00000000E+00  0.00000000E+00  0.00000000E+00
0.00000000E+00  0.00000000E+00  0.00000000E+00
0.00000000E+00  0.00000000E+00  0.00000000E+00
0.00000000E+00  0.00000000E+00  0.00000000E+00
0.00000000E+00  0.00000000E+00  0.00000000E+00
0.00000000E+00  0.00000000E+00  0.00000000E+00
```

References

(1) Hinuma, Y.; Pizzi, G.; Kumagai, Y.; Oba, F.; Tanaka, I. Band Structure Diagram Paths Based on Crystallography. *Computational Materials Science* **2017**, *128*, 140–184. https://doi.org/10.1016/j.commatsci.2016.10.015.
(2) Togo, A.; Shinohara, K.; Tanaka, I. Spglib : A Software Library for Crystal Symmetry Search. arXiv 2018. https://doi.org/10.48550/ARXIV.1808.01590.
(3) Yuval Elbaz; Maytal Caspary Toroker. From Density Functional Theory to Machine Learning Predictive Models for Electrical Properties of Spinel Oxides. *Scientific Reports* **2024**. https://doi.org/10.1038/s41598-024-62788-4.
