# Supplementary material for: The Effect of Sulfur Vacancy Distribution on Charge Transport across MoS2 Monolayers: A Quantum Mechanical Study
Source: ACS Mater Au. 2025 Jun 6;5(4):641–55. doi: 10.1021/acsmaterialsau.4c00171 (PMC12257400; doi:10.1021/acsmaterialsau.4c00171)
Supplement: Supplementary file 2 [file mg4c00171_si_002.zip › Full_factorial_-_Fit_Least_Squares.html]

Full factorial - Data sum for DOE - Oct2024 - Fit Least Squares 3
